# Analysis of factors influencing changes in medical behavior under the context of DRG payment method reform: a structural equation modeling approach

**DOI:** 10.3389/fpubh.2025.1524215

**Published:** 2025-09-12

**Authors:** Chen Jiang, Dawei Qin, JinPeng Zhang, Yanpeng Guan, Fengming Li, Liangmei Jiang

**Affiliations:** ^1^School of Management, Shandong Second Medical University, Weifang, China; ^2^Linyi People’s Hospital, Linyi, China

**Keywords:** DRG, healthcare professionals, medical behavior, structural equation model, cognitive evaluations

## Abstract

**Objectives:**

This study aims to assess the understanding of policies, cognitive awareness, and medical behavior patterns among healthcare workers about the reform of the diagnosis-related group (DRG) payment method. A questionnaire survey was conducted to examine how policy comprehension and cognition influence changes in medical behavior, targeting medical personnel in a northern Chinese city.

**Methods:**

An online survey was administered from November to December 2023, covering primary, secondary, and tertiary medical institutions in a city that had implemented DRG payments. The survey gathered demographic data and information on policy comprehension, medical behavior patterns, and policy cognition. Structural equation modeling was employed to analyze the relationships among these factors and their impact on shifts in medical behavior.

**Results:**

The findings revealed that policy comprehension did not significantly affect policy cognition. Nonetheless, a higher level of policy comprehension was linked to a negative influence on positive medical behavior (*t* = −0.115, *p* < 0.05), and a positive impact on negative medical behavior (*t* = 3.746, *p* < 0.001). Conversely, positive policy cognition was found to promote positive medical behavior (*t* = 10.756, *p* < 0.001), while negative policy cognition was associated with an increase in negative behavior (*t* = 12.282, *p* < 0.001).

**Conclusion:**

Behavioral adaptations to DRG reform are driven primarily by cognitive evaluations (positive/negative policy perceptions), not mere policy knowledge. Contrary to expectations, deeper policy understanding correlates with increased negative medical behavior and reduced positive behavior, suggesting that technical training alone may inadvertently incentivize gaming under financial pressures. Positive cognition strongly promotes desirable behaviors, while negative cognition exacerbates detrimental practices. To optimize reform success, China must prioritize value-oriented training (emphasizing DRG’s systemic goals over mechanics) and implement intelligent auditing mechanisms to curb high-risk behaviors during transition.

## 1 Introduction

The medical insurance payment system serves as a crucial mechanism for regulating medical behavior and guiding the distribution of healthcare resources (1). Different payment models impact medical behavior through distinct incentive and constraint structures (2). In a pay-per-item system, clearly defined service charges motivate medical professionals, encouraging healthcare institutions to develop medical technologies. This approach expands service offerings, leading to improvements in the quantity, quality, and efficiency of healthcare services. However, this payment model may also have negative effects, such as over-treatment, difficulties in controlling rising healthcare costs, and potential threats to the sustainability of health insurance funds (3).

In contrast, the diagnosis-related group (DRG) payment system focuses on resource conservation. This approach allows healthcare providers to enhance services by implementing hierarchical diagnosis and treatment methods (4), optimizing bed usage (5, 6), and reducing the average length of hospital stays (7). Although these strategies can boost service capacity and efficiency, they may also lead to unintended behaviors such as undertreatment, avoiding high-risk patients, lowering admission thresholds, cost-shifting, and manipulating hospitalization coding (8–10).

The DRG payment system is a key element in China’s ongoing healthcare payment reform, and its success depends on its ability to influence the behavior of healthcare providers (11). As central figures in the medical process, healthcare professionals play a pivotal role in the implementation of DRG reform. Their treatment decisions are shaped by both professional ethics and personal incentives (12, 13). This study aims to explore the main factors driving changes in medical behavior within the framework of DRG reform and to offer recommendations for regulating and overseeing medical practices. These efforts will support the successful execution of DRG payment reform and the advancement of high-quality public hospital development.

## 2 Materials and methods

### 2.1 Research objectives

This study focuses on a city in northern China as a case study to assess the implementation of the DRG payment system. The research sample includes healthcare organizations actively involved in this payment model. Following the full-scale rollout of the DRG system in July 2022, a total of 375 healthcare facilities participated, comprising 8 tertiary hospitals, 87 secondary hospitals, and 280 primary hospitals. Data collection took place in November 2023 via the Questionstar online platform, targeting professionals in medicine, nursing, medical technology, and hospital administration.

### 2.2 Research methods

#### 2.2.1 Questionnaire survey

The study utilized a questionnaire titled “Survey on medical behavior and its influencing factors in the context of DRG payment method reform,” distributed through Questionstar. The survey was divided into four sections: demographic characteristics of respondents, such as gender, age, position, job title, qualifications, and years of experience; cognitive understanding of the DRG payment method and hospital management practices; the perceived impact of DRG payment reform on medical behaviors; and policy knowledge related to the DRG payment reform.

Responses were collected using the 5-point Likert scale. In the first section, which assessed policy understanding, three items were measured, with 1 indicating no understanding and 5 indicating full understanding. The second section, focused on policy cognition, contained eight items, where positive cognition was rated from 1 (strongly disagree) to 5 (strongly agree), while negative cognition items were scored inversely. The third section evaluated 28 diagnostic and treatment behaviors, with 15 positive and 13 negative behaviors. Respondents rated the probability of these behaviors occurring under the DRG reform, using a scale of 1 (very unlikely) to 5 (very likely) for positive behaviors, with the reverse scoring for negative behaviors.

### 2.3 Statistical methods

Statistical analyses were conducted using SPSS 26.0 software, focusing on descriptive statistics and reliability analysis. Descriptive statistics were employed to interpret foundational research outcomes, providing an overview of the collected data, while reliability analysis was used to assess the internal consistency and validity of the questionnaire. A significance level of *p* < 0.05 was set to determine the statistical significance of the findings.

#### 2.3.1 Structural equation modeling

Amos 24.0 software was utilized to construct a structural equation model aimed at examining the factors that influence medical behavior patterns among healthcare professionals in the context of DRG payment system reform. The model underwent validation and adjustments, with evaluations of structural validity, convergent validity, and discriminant validity. A threshold of *p* < 0.05 was used as the reference standard for assessing model significance.

#### 2.3.2 Quality control

The questionnaire was refined through multiple iterations, guided by a thorough review of the relevant literature, expert consultations, and panel discussions, ensuring its scientific rigor. The questionnaire’s reliability was validated through testing. Out of the 695 questionnaires collected online, invalid responses—identified by factors such as excessively short completion times or repetitive answers—were excluded. This process resulted in 675 valid responses, yielding a validity rate of 97.1%.

### 2.4 Definition of concepts and development of theoretical models

#### 2.4.1 Introduction of the theoretical model

Cognitive behavioral theory seeks to correct maladaptive thoughts and behaviors in individuals by merging aspects of cognitive and behavioral theories. Aaron Beck’s cognitive therapy and Albert Ellis’s rational emotive therapy are central to this approach, particularly through the use of the “ABC framework” of emotional theory. According to this framework:

A (Activating event): This represents events or attitudes that occur, which may be subject to change.

B (Belief): These are the cognitive responses individuals have toward the activating events, which can be either positive or negative.

C (Consequence): The resulting emotional and behavioral responses stemming from the beliefs about the event.

The ABC theory suggests that emotional and behavioral consequences (C) are influenced not solely by the events (A), but by the individual’s interpretations and beliefs (B). Cognitive behavioral therapy emphasizes the role of cognition in addressing psychological and behavioral challenges. It utilizes cognitive restructuring and behavioral interventions to correct distorted thinking patterns, foster accurate perception, and achieved positive emotional and behavioral outcomes.

#### 2.4.2 Theoretical model construction

This study examines the impact of healthcare professionals’ understanding and cognition of policy (both positive and negative) on their medical behavior tendencies. Based on this investigation, the following hypotheses are put forward:

*H1*: A higher degree of policy understanding positively influences healthcare professionals’ perceptions of the DRG policy.

The DRG system includes various components such as grouping rules, payment regulations, and guidelines for weighted rate calculation, all designed to manage the DRG payment framework. According to cognitive behavioral theory, healthcare professionals’ attitudes toward DRG payment reform reflect their policy cognition, which can be either positive or negative. The literature shows that healthcare staff with a more in-depth understanding of DRG policies are more inclined to recognize their value (14).

*H2*: A greater understanding of the policy positively influences positive medical behaviors and negatively influences negative medical behavior.

One of the main goals of the DRG payment reform is to standardize healthcare practices and ensure efficient allocation of medical resources (15). Research indicates that when clinicians have a solid grasp of DRG payment policies, they are more likely to follow standardized practices, such as clinical pathways (16).

*H3*: The direction of policy cognition aligns with medical behavior tendencies; specifically, positive cognition fosters positive medical behavior, while negative cognition promotes negative medical behavior.

Various payment systems affect medical behaviors through different incentives and constraints. The DRG reform aims to curb excessive medical practices and control rising healthcare costs, creating a favorable environment for healthcare professionals, insurers, and patients. However, it may unintentionally trigger new irregular medical practices. The degree to which healthcare providers acknowledge the scientific and standardized nature of DRG payments significantly influences their behavioral adaptations. Related research shows that an appreciation for the value of DRG payments is associated with stronger support for the reform and adherence to standardized clinical practices (17, 18).

#### 2.4.3 Selection and formulation of measurement questions

The selection of quantifiable observational variables, representing latent variables, was based on a thorough review of the literature, professional research, and expert consultations. In total, 23 observational variables were identified, which included questions on policy understanding, policy cognition (with positive and negative aspects), and medical behavior tendencies. Specifically, the study incorporated three questions on policy understanding, eight questions on policy cognition (five addressing positive cognition and three addressing negative cognition), and 12 questions related to medical behavior tendencies, with eight addressing positive behaviors and four addressing negative behaviors (see Table 1).

**Table 1:** Description of measurement question options.

Measurement dimensions	Latent variable	Observational variables
	Level of policy understanding	Do you understand the concept of DRG?
		Do you understand the rationale and mechanics of DRG payments?
		Are you aware of the City DRG payment overall settlement program?
Policy awareness	Positive policy perceptions	DRG payment methods can reduce healthcare costs
		DRG payment methods can improve healthcare delivery efficiency
		DRG payment methods can improve health care quality
		DRG payment methods help improve patient satisfaction
		DRG payment methods can regulate medical practices
	Negative policy perceptions	DRG payment methods increase workload for medical professionals
		DRG payment methods limit physicians from taking the most appropriate care of patients
		DRG payment methods can lead to physicians picking patients
Medical behavioral tendencies	Positive medical behavior	In the context of DRG reform, the consultation process will actively reduce the length of hospitalization
		In the context of DRG reform, the types of medicines used in the consultation process will be actively reduced
		In the context of DRG reform, the number of days of medication use will be proactively reduced during the consultation process
		In the context of DRG reform, the use of complementary medicines will be actively reduced during the consultation process
		In the context of DRG reform, proactive reduction in the use of prophylactic antimicrobials will occur during the consultation process
		In the context of DRG reform, the use of proprietary Chinese medicines or Chinese medicine injections will be reduced voluntarily in the course of consultation and treatment
		In the context of DRG reform, the use of essential medicines will be increased proactively in the diagnosis and treatment process
		In the context of DRG reform, there will be a proactive reduction in the use of high-value consumables in the diagnosis and treatment process
	Negative medical behavior	In the context of DRG reform, the consultation process will shirk patients who may be overspent
		DRG reform context, the diagnostic process reduces discharge criteria (inappropriately early discharge)
		In the context of DRG reform, the diagnosis and treatment process breaks down hospitalization
		DRG reform context will increase patient referrals during the consultation process

### 2.5 Theoretical framework

**Figure 1:**
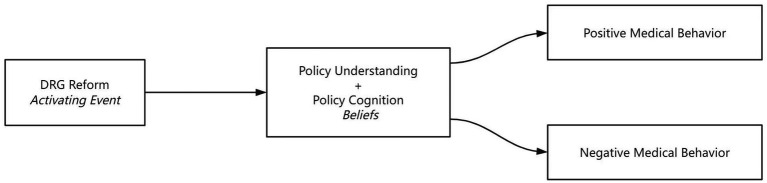
Theoretical model diagram.

Grounded in Beck’s cognitive therapy model (19), this study conceptualizes Diagnosis-Related Group (DRG) payment reform as an activating event (Event A) triggering cognitive reappraisal among healthcare professionals. Integrating Ellis’ ABC framework (20), we posit that:

1. Policy understanding (knowledge of DRG mechanics) and policy cognition (attitudinal evaluations) constitute belief systems (Beliefs B);
2. These cognitive mediators directly shape medical behavior tendencies (Consequences C), consistent with empirically validated cognition-behavior pathways (21). Contextualized by Rogers’ Diffusion of Innovations Theory (22), this framework elucidates how policy comprehension interacts with perceived incentives (e.g., cost-saving rewards) and implementation complexity (e.g., coding challenges) to drive behavioral adaptations—a mechanism observed cross-nationally (23). This synthesized model addresses China-specific implementation barriers while maintaining cross-contextual explanatory power (see Figure 1).

## 3 Results

### 3.1 Summary of fundamental conditions

In a survey conducted with 675 medical personnel, 293 (42.2%) were male, while 402 (57.8%) were female. Age distribution showed that 311 participants (44.7%) were aged between 30 and 40 years. Regarding educational qualifications, 446 respondents (64.2%) held undergraduate degrees, and 124 (17.8%) had specialized education. Professionally, 364 respondents (52.4%) were physicians, and 184 (26.5%) were nurses. A total of 235 respondents (33.8%) held junior or intermediate professional titles, with an equal number having 11 to 20 years of work experience. Of the total, 144 (20.7%) worked in primary hospitals, 300 (43.2%) in secondary hospitals, and 251 (36.1%) in tertiary hospitals. Furthermore, 527 respondents (75.8%) were employed in public hospitals, while 168 (24.2%) worked in private hospitals, as presented in Table 2.

**Table 2:** Demographics of the respondents.

Items		Number/person	Percentage/%
Distinguishing between the sexes	Male	293	42.2
	Female	402	57.8
Age group	20–30	129	18.6
	30–40	311	44.7
	40–50	198	28.5
	50–60	56	8.1
Education attainment	60 or more	1	0.1
	Vocational secondary school	22	3.2
	Specialized training school	124	17.8
	Undergraduate	446	64.2
	Master’s degree student	94	13.5
	PhD student	8	1.2
	Other	1	0.1
Job	Surgeon	364	52.4
	Physiotherapists	184	26.5
	Medical technologist	66	9.5
	Hospital administrators	52	7.5
	Other	29	4.2
Title	Junior ranking	235	33.8
	Middle level (in a hierarchy)	235	33.8
	Deputy high ranking	160	23
	High ranking	31	4.5
	Other	34	4.9
Duties	Heads of clinical departments	118	17
	Head nurse	52	7.5
	Director of administration	25	3.6
	Hospital faculty leadership	24	3.5
	General staff	476	68.5
Years of experience	1–5 years	156	22.4
	6–10 years	166	23.9
	11–20 years	239	34.4
	More than 20 years	134	19.3
Hospital level	Level 1	144	20.7
	Level 2	300	43.2
	Level 3	251	36.1
Nature of hospital	Public hospitals	527	75.8
	Private hospital	168	24.2

### 3.2 Reliability assessment of the questionnaire

The reliability of the scale was evaluated using Cronbach’s Alpha method, which revealed that the value for each dimension exceeded 0.7, indicating strong internal consistency and good reliability. The validity of the scale was examined through exploratory factor analysis and confirmatory factor analysis. The results of the exploratory factor analysis showed that the KMO value (0.684) for the policy understanding scale was acceptable (24, 25). The KMO values for both the medical behavior tendency and policy awareness scales were greater than acceptable thresholds(0.8), and the *p*-values for Bartlett’s test of sphericity were significant (*P* < 0.01), confirming that factor analysis was suitable. Further analysis shows that the policy understanding scale extracted one factor with a cumulative variance explained rate of 85.36%. The medical behavior tendency scale extracted two factors, with a cumulative variance explained rate of 67.56%, and the policy awareness scale also extracted two factors, demonstrating a cumulative variance rate of 72.33%. All of the factor loadings exceeded 0.6, indicating a strong extraction effect. For structural validity, the factor loadings for all of the measurement questions were above 0.5, suggesting that these questions effectively captured the dimensional information they were intended to measure. Regarding convergent validity, the CR values for the five latent variables exceeded 0.6, demonstrating acceptable internal consistency. As for discriminant validity, aside from a less distinct separation between negative policy perceptions and negative medical behaviors, the square root of the AVE for the remaining four latent variables was greater than their correlation coefficients with other variables, suggesting that the scales had acceptable discriminant validity.

### 3.3 Results of structural equation modeling analysis

#### 3.3.1 Model fit

As indicated in Table 3, the *X*^2^/*df* value was within the acceptable range, indicating an ideal fit for the model. The RMSEA was approximately equal to 0.05, further confirming a good model fit. The GFI exceeded 0.9, reflecting a well-fitting result, while the NFI and AGFI both demonstrated good fit as well, as they exceeded acceptable thresholds. Finally, the CFI also indicated good adaptability. Collectively, these indices suggest that the overall model fits well.

**Table 3:** Model fitting effects.

Fitness index	Metric	Fit
Chi-square value (CMIN)	637.638	
Degrees of freedom (DF)	215	
Absolute fit index (P)	0.000	
CMIN/DF	2.966	<3, acceptable
Goodness of fit index (GFI)	0.924	>0.9, good fit
Relative fit index (NFI)	0.940	>0.9, good fit
Amended goodness of fit index (AGFI)	0.902	>0.9, good fit
Comparative fit index (CFI)	0.959	>0.9, good fit
Root mean square error of approximation (RMSEA)	0.054	<0.08, acceptable

#### 3.3.2 Pathway analysis

**Figure 2:**
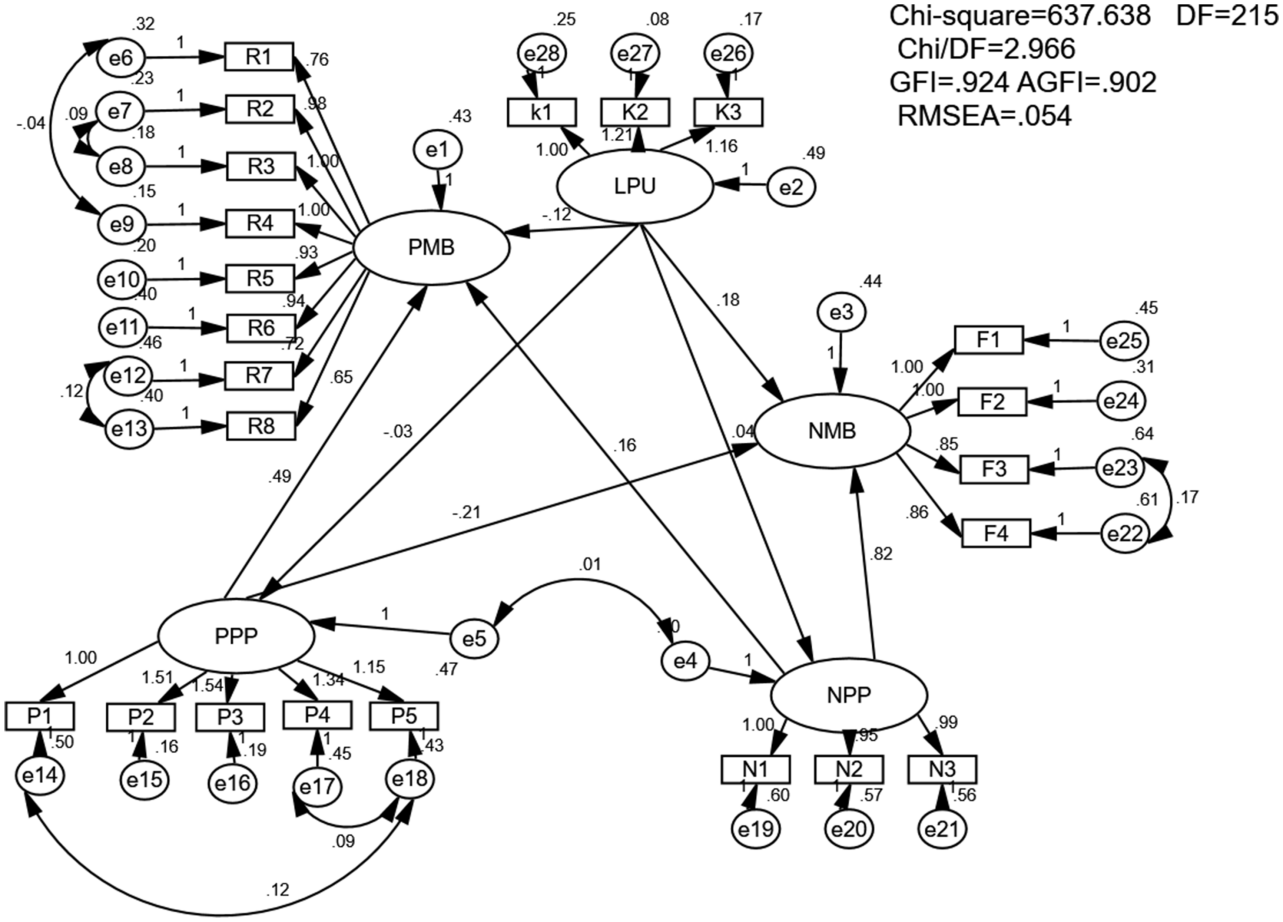
Structural equation model of medical behavior change of medical personnel.

The study explored the relationships among policy understanding, policy cognition, and medical behavior tendencies among healthcare professionals in the context of DRG payment reform. A structural equation model was developed to assess these relationships (see Figure 2). The analysis of standardized factor loadings for the five dimensions—policy understanding, positive medical behavior, negative medical behavior, positive policy cognition, and negative policy cognition—revealed values exceeding 0.6 for all dimensions, indicating strong reliability. The subject matter reliability values were also above 0.36, demonstrating that the model effectively explained the variables used in the study.

Direct effects were tested using structural equations, with the significance level set at *p* < 0.05. Path coefficients were estimated for the relationships influencing the behavioral tendencies of medical staff. Specifically, the standardized path coefficients for the following relationships were calculated: level of policy understanding (LPU) → positive medical behavior (PMB): −0.115, level of policy understanding (LPU) → negative medical behavior (NMB): 0.136, positive policy perceptions (PPP) → positive medical behavior (PMB): 0.444, positive policy perceptions (PPP) → negative medical behavior (NMB): −0.154, negative policy perceptions (NPP) → positive medical behavior (PMB): 0.159, negative policy perceptions (NPP) → negative medical behavior (NMB): 0.674. The level of policy understanding did not significantly affect policy cognition. However, it had a minor negative effect on positive medical behavior and a slight positive effect on negative medical behavior. Positive policy cognition had a notable positive impact on positive medical behavior and a small significant negative effect on negative medical behavior. Conversely, negative policy cognition had a minor positive influence on positive medical behavior and a substantial positive influence on negative medical behavior (see Table 4). In summary, hypotheses H1 and H2 were not supported, while hypothesis H3 was confirmed, leading to the conclusions that follow.

**Table 4:** Path coefficients of structural equation modeling of medical behavioral tendencies.

Variable relationship	Path factor	*t*-value	*p*-value
Level of policy understanding → Positive policy perceptions	−0.028	−0.689	0.491
Level of policy understanding → Negative policy perceptions	0.037	0.799	0.424
Level of policy understanding → Positive medical behavior	−0.115	−3.111	0.002
Level of policy understanding → Negative medical behavior	0.136	3.746	0
Positive policy perceptions → Positive medical behavior	0.444	10.756	0
Positive policy perceptions → Negative medical behavior	−0.154	−4.146	0
Negative policy perceptions → Positive medical behavior	0.159	3.857	0
Negative policy perceptions → Negative medical behavior	0.674	12.282	0

Policy understanding and policy cognition: The level of policy understanding did not notably affect policy cognition. This could be due to current DRG payment training, which focuses on theory and practices but lacks sufficient emphasis on the policy’s background, significance, and broader implications. Therefore, it is important to improve training on the ideological and political aspects of payment reform to enhance medical personnel’s understanding of the DRG payment system.

Influence of policy understanding on medical behavior: Greater policy understanding might unintentionally lead to an increase in negative medical behaviors. In the early stages of DRG bundled payment reform, changes in the profit mechanisms of healthcare institutions may have prompted some medical staff to exploit policy loopholes for financial gain. Additionally, weaknesses in the regulatory system and insufficient internal controls likely contributed to the rise of negative behaviors.

Policy cognition and medical behavior: Positive policy cognition encourages positive medical behaviors, while negative policy cognition fosters negative behaviors. Importantly, even though negative policy perceptions may increase negative behaviors, healthcare professionals may still maintain high standards due to ethical training, professional integrity, and robust medical quality and safety systems.

## 4 Discussion

### 4.1 The necessity for transforming health care policy training

The finding that policy knowledge did not translate into favorable policy perceptions highlights a critical gap in current training approaches. This gap, observed in our Chinese context, was also reported during early DRG implementation phases internationally (26). Consistent with our results, studies from Germany (27) and the US (28) indicate that initial training often overemphasizes technical components—such as coding, grouping rules, financial implications, and “gaming” strategies to minimize losses—while neglecting broader policy objectives like efficiency, equity, and quality. This narrow focus can inadvertently cultivate negative perceptions and opportunistic behaviors, as staff primarily view DRG as a financial constraint rather than a tool for systemic improvement (29).

This points to a universal challenge: Effective payment reform requires training that extends beyond technical mechanics to address the fundamental purpose of the reform, aligning professional values with policy goals. Programs emphasizing how DRG advances professional objectives—such as efficient, high-quality care—have proven more successful in fostering positive engagement and desired behavioral change. Examples include implementations integrating DRG with clinical pathways and quality metrics (4, 23). China’s experience underscores the urgent need to shift toward value-oriented training, a lesson critical for any system adopting complex payment reforms.

#### 4.1.1 Behavioral economics perspective

Our findings align with prospect theory (27), wherein individuals overweight potential losses relative to equivalent gains. Under DRG cost-containment pressures:

Loss aversion drives physicians to avoid high-cost patients. A German study supports this by demonstrating that doctors adjust care delivery to mitigate financial risk under DRG systems, highlighting a conflict between cost-efficiency objectives and loss-averse clinical decision-making (28).

The framing effect prioritizes coding optimization over clinical needs. In practice, both coders and physicians responsible for filling in key clinical information tend to target high-profit DRG categories (30). Some respondents also noted that the DRG policy has significantly increased administrative burdens (26).

This behavioral distortion escalates when providers frame DRG payments as financial penalties rather than efficiency incentives (29).

#### 4.1.2 Analysis of factors contributing to negative medical behavior despite increased policy understanding

The counterintuitive association between deeper policy understanding and increased negative behaviors aligns with experiences from other health systems implementing DRGs. The ‘fraud triangle’ framework effectively explains the mechanisms—pressure, opportunity, and rationalization—underlying this phenomenon. Pressure arising from linking income to cost-control targets is notably exacerbated in contexts like China, where physician compensation is often directly tied to departmental or hospital financial performance. This contrasts with some European systems, where stronger salary-based compensation models reduce this linkage (31). Opportunity, stemming from regulatory gaps, complex rules, and challenges in detecting sophisticated gaming (e.g., upcoding, patient selection, inappropriate transfers), presents a near-universal challenge during early DRG adoption, as documented in the US (28), France (32), and Australia. Rationalization (e.g., “everyone does it, ““necessary to survive,” “the system is flawed”) is also widely reported (26, 29). These findings indicate that the emergence of negative behaviors alongside increased system knowledge reflects a broadly relevant phenomenon, though its intensity and specific manifestations are influenced by contextual factors such as compensation structures and pre-existing regulatory maturity. Addressing this challenge requires adaptive, proactive auditing mechanisms that evolve alongside payment reforms, leveraging data analytics—as highlighted in our study and increasingly utilized in mature DRG systems (33).

### 4.2 Enhanced optimization and improvement of the DRG payment policy

Given the paramount influence of policy cognition on behavior, fostering positive perceptions through continuous refinement is essential. Our recommendations align with international lessons: First, refining grouping rules and pricing—fundamental to DRG success—requires China to implement continuous, data-driven iteration akin to annual MS-DRG (US) or AR-DRG (Australia) updates. Critically, this must include robust risk-adjustment methodologies (e.g., G-DRG’s complexity models) to mitigate incentives for patient selection. Second, standardizing care processes through clinical pathways and hierarchical diagnosis/treatment systems (4, 34) is vital, yet must address the universal challenge of ensuring these standards are evidence-based and clinically relevant, avoiding perceptions of restrictive “cookbook medicine” that undermine professional judgment. Success hinges on clinician buy-in and flexibility, as demonstrated in systems where pathways are co-developed with practitioners. Finally, strengthening oversight via targeted supervision and intelligent auditing using big data (33) must accelerate. Mirroring mature systems (e.g., US CMS Program Integrity contractors using predictive analytics), China should focus not only on punitive measures but also on education and systemic feedback to close loopholes—effectiveness internationally depends on adequate resources, technical expertise, and legal frameworks. Collectively, these steps position DRG not as a rigid constraint but as a dynamic tool for system improvement, balancing standardization with clinical autonomy and data-driven accountability.

### 4.3 International perspectives on aligning payment reform and clinical practice

A critical challenge underscored by our findings and international evidence is ensuring that payment system design and implementation align with clinical realities to support, rather than hinder, appropriate care. Globally, common pitfalls include DRG weights failing to accurately reflect the complexity or cost of specific conditions (leading to under- or overpayment), inadequate risk adjustment incentivizing avoidance of high-cost or complex patients (8, 9), and excessive administrative burdens—such as complex coding requirements—diverting time from patient care and generating resentment (26); our finding that negative policy perceptions strongly drive negative behavior highlights the detrimental impact of such misalignment. China’s strategy of refining grouping rules and standardizing clinical pathways (4, 34) parallels approaches elsewhere, exemplified by the US Medicare system’s continuous updates to MS-DRG definitions incorporating technological advances, and Germany’s G-DRG system utilizing sophisticated morbidity-oriented risk adjustment to better account for patient severity. Nevertheless, achieving true alignment remains an ongoing global challenge, necessitating proactive dialogue and collaboration among payers, providers, and clinicians to collaboratively design and refine payment models and supporting infrastructure (e.g., IT systems, clinical documentation standards). Essential mechanisms include technical expert panels (used in US DRG updates) and pilot programs incorporating provider feedback (demonstrated in Chinese local pilots). Crucially, China’s experience affirms that successful DRG implementation extends beyond technical grouping and pricing; it is fundamentally a complex socio-technical process demanding careful attention to integrating payment rules with clinical workflow, preserving professional autonomy, and responding to patient needs (35, 36).

### 4.4 Strengthening the integration of health insurance policy and medical behavior to promote positive outcomes

Medical insurance payment policies are designed to cover all cases based on standardized payment rules, while medical behaviors exhibit clear individual differences, particularly in personalized diagnostic and treatment plans and associated costs. As a result, discrepancies often arise between medical behaviors and the cost structures defined by insurance payment policies. These differences need to be addressed using big data and statistical theories, particularly for special cases that may not be covered by payment policies. Timely communication and negotiation between medical institutions and insurers are necessary to resolve issues related to specific disease-based payment policies, high-cost multiplier payments, unstable disease group payments, and the reimbursement of new technologies and drugs. Addressing these discrepancies is crucial to ensuring a positive cycle of integration and advancements between disease-based payment policies and the development of medical technologies (33).

## 5 Conclusion

Behavioral adaptations to DRG reform among healthcare professionals are driven primarily by cognitive evaluations of the policy, not merely knowledge acquisition. This core finding reveals a fundamental psychological mechanism likely transferable across diverse systems implementing similar payment reforms. While manifestations of negative behaviors and the intensity of specific drivers (e.g., compensation linkage) reflect China’s unique context, the underlying dynamics—the critical role of cognition aligned with professional values; the counterproductive potential of purely technical training in incentivizing gaming; the necessity of robust auditing; and the imperative to align payment rules with clinical practice—exhibit broad cross-jurisdictional relevance. China’s experience reinforces that successful DRG implementation requires addressing human and systemic factors beyond technical mechanics. Consequently, key recommendations include: emphasizing value-oriented training to foster policy buy-in (4, 23); continuously refining grouping and pricing through data and clinician input, as seen in MS-DRG/AR-DRG updates; strengthening intelligent auditing using big data analytics (33); and fostering structured collaboration between payers and providers to harmonize policy with practice (37). Systematically implementing these strategies offers critical insights not only for optimizing China’s reform but also for guiding the global transition toward value-based payment.

## Data Availability

The original contributions presented in the study are included in the article/Supplementary material, further inquiries can be directed to the corresponding authors.

## References

[1] General Office of the State Council. Guiding opinions on further deepening the reform of basic medical insurance payment methods [EB/OL] (2017). 1. Available at: http://www.gov.cn/zhengce/content/2017-06/28/content_5206315.htm (Accessed September 25, 2024).

[2] Lele L Mengyi T Hongwu T. Analysis on the operation logic, influence mechanism and lmplementation effect of payment mode reform of medical insurance in China. China Health Econ. (2022) 41:10–21.

[3] Xinyuan Z Yuli H Yunjie B Miaojie Q Xing L. The experimental research on the influence of the transform from retrospective reimbursement to prospective reimbursement on physicians' behaviour. China Health Econ. (2020) 39:28–32.

[4] Bingfeng S Qiang F Shifeng F Shunshun C Jianwei Y Tao L. Utility analysis of DRGs reform in the clinical pathway. China Health Std Manag. (2020) 11:6–8.

[5] Gao C Xu F Liu GG. Payment reform and changes in health care in China. Soc Sci Med. (2014) 111:10–6. doi: 10.1016/j.socscimed.2014.03.035, PMID: 24735721

[6] Pan Z Y J G J Xiang B J F Z H. Practice on intracranial vascular surgery cost and efficiency management under DRGs payment system. Chin Hosp. (2019) 23:60–2. doi: 10.19660/j.issn.1671-0592.2019.06.20

[7] Yiting W Guangbo Y. An empirical analysis of cost reduction and efficiency increase in DRG reform based on factor analysis. China Hosp. (2022) 26:29–31. doi: 10.19660/j.issn.1671-0592.2022.9.08

[8] Chao C. A Study on Factors Influencing Clinical Physicians’ Diagnostic and Therapeutic Practices [D]. Shandong: Shandong University (2007). doi: 10.7666/d.Y1066003

[9] Jian W Lu M Chan KY Poon AN Han W Hu M. Payment reform pilot in Beijing hospitals reduced expenditures and out-of-pocket payments per admission. Health Aff (Millwood). (2015) 34:1745–52. doi: 10.1377/hlthaff.2015.0074, PMID: 26438752

[10] Wang H Zhang L Yip W Hsiao W. An experiment in payment reform for doctors in rural China reduced some unnecessary care but did not lower total costs. Health Aff (Millwood). (2011) 30:2427–36. doi: 10.1377/hlthaff.2009.0022, PMID: 22147872

[11] Xinyuan Z Yuli H. Research progress on the impact of mixed payment on medical service behavior. China Health Econ. (2019) 38:23–6.

[12] Bauchner H Simpson L Chessare J. Changing physician behaviour. Arch Dis Child. (2001) 84:459–62. doi: 10.1136/adc.84.6.459, PMID: 11369556 PMC1718805

[13] Rodwin MA. Financial incentives for doctors. BMJ. (2004) 328:1328–9. doi: 10.1136/bmj.328.7452.1328, PMID: 15178588 PMC420273

[14] Song Y Yanhong Z Xu W Kaiquan Z Wu J Chen J. Study on the influencing factors of medical service behavior norms in the context of DRG payment-based on structural equation model. Health Econ Res. (2023) 40:50–5. doi: 10.14055/j.cnki.33-1056/f.2023.07.006

[15] Qin J. The experience and enlightenment of DRG pricing and payment policy design and implementation. China Health Econ. (2022) 41:6–11.

[16] Yan M. Study on the impact of DRG payment mode reform on medical behavior of doctors. Chin Contemp Med. (2021) 28:205–13.

[17] Leung CK Liyan J Yong W Xiaohui X. Investigation on clinicians' cognition and demand for DRG payment reform. China Med Insur. (2021) 1:66–8. doi: 10.19546/j.issn.1674-3830.2021.1.015

[18] Ye L Qiuyan a. Survey and analysis of DRG implementation in Lijiang M Hospital. World Lab Secur. (2020) 18:38–42.

[19] Beck AT Haigh EA. Advances in cognitive theory and therapy: the generic cognitive model. Annu Rev Clin Psychol. (2014) 10:1–24. doi: 10.1146/annurev-clinpsy-032813-153734, PMID: 24387236

[20] Ellis A. Rational psychotherapy and individual psychology. J Individ Psychol. (1957) 13:38–44.

[21] Birken SA Powell BJ Presseau J Kirk MA Lorencatto F Gould NJ. Combined use of the consolidated framework for implementation research (CFIR) and the theoretical domains framework (TDF): a systematic review. Implement Sci. (2017) 12:2. doi: 10.1186/s13012-016-0534-z, PMID: 28057049 PMC5217749

[22] Rogers EM Singhal A Quinlan MM. Diffusion of innovations[M]//An integrated approach to communication theory and research. Routledge, (2014):432–448.

[23] Fisher ES McClellan MB Bertko J Lieberman SM Lee JJ Lewis JL. Fostering accountable health care: moving forward in medicare. Health Aff (Millwood). (2009) 28:w219–31. doi: 10.1377/hlthaff.28.2.w219, PMID: 19174383 PMC2656392

[24] Leixue Z Jianchao Y Zhichao L Hongsheng M. Influencing factor analysis of day surgery developing towards same-day surgery based on structural equation modeling. West China Univ Med. (2022) 37:182–8.

[25] Xinming Z Dei B Binglei X Hang M. A study of the effectiveness of epidemic prevention policies on public transit usage based on the theory of planned behaviors. Transp Inf Saf. (2021) 39:117–25. doi: 10.3963/j.jssn.1674-4861.2021.06.014

[26] Tummers L. Policy alienation: Analyzing the experiences of public professionals with new policies. Rotterdam: Erasmus University Rotterdam (2012) (Forthcoming).

[27] Kahneman D Tversky A. Prospect theory: An analysis of decision under risk. In: MacLean LC Ziemba WT (Eds.), Handbook of the fundamentals of financial decision making Part I: World Scientific. (2013). 99–127.

[28] Hensen P Furstenberg T Luger TA Steinhoff M Roeder N. Case mix measures and diagnosis-related groups: opportunities and threats for inpatient dermatology. J Eur Acad Dermatol Venereol. (2005) 19:582–8. doi: 10.1111/j.1468-3083.2005.01258.x, PMID: 16164713

[29] Frank RG Zeckhauser RJ. Custom-made versus ready-to-wear treatments: behavioral propensities in physicians' choices. J Health Econ. (2009) 28:1121–33. doi: 10.1016/j.jhealeco.2009.07.005 18031852

[30] Milcent C. From downcoding to upcoding: DRG based payment in hospitals. Int J Health Econ Manag. (2021) 21:1–26. doi: 10.1007/s10754-020-09287-x, PMID: 33128657

[31] Shuanghui C Xinyi X Haibo W. Cause analysis and governance strategy of negative medical behaviors under the background of DRG reform. Med Philos. (2022) 43:37–40. doi: 10.12014/j.issn.1002-0772.2022.20.08

[32] Xiang C Ying H Jingyuan X Jinghua Z. Research and review on positive incentive mechanism of medical insurance payment system reform to general practitioners. China Health Econ. (2021) 40:67–70.

[33] Bin C Zhaofang Z Bin C Jiyuan S Qian S Lin Y. Discussion on the supervisory mechanism of health insurance fund under DRG payment model. China Health Econ. (2022) 41:33–6.

[34] Zhang J Xiao L Huang M. Anatomy and implications of the US MS-DRG grouping rule. Chin J Hosp Manag. (2022) 38:631–6. doi: 10.3760/cma.j.cn111325-20220233-00147

[35] Yong C Xiaoqian J Yuting N Zhaoyang Z Yunwu Z Honing W. Research on the current situation and strategies of the hierarchical medical system under the DRG payment mode. China Hosp. (2023) 27:44–8. doi: 10.19660/j.issn.1671-0592.2023.10.12

[36] Qihui W Tianqiu H Wu L. Research on the legalization of the formulation of diagnostic and treatment standards in China. Med Soc. (2023) 36:110–5. doi: 10.13723/j.yxysh.2023.08.020

[37] Ling Z Zikun D. Innovative practice of big data medical insurance fund supervision under DRG payment. Health Econ Res. (2021) 38:37–40. doi: 10.14055/j.cnki.33-1056/f.2021.12.010

