# Supplementary material for: Analysis of factors influencing changes in medical behavior under the context of DRG payment method reform: a structural equation modeling approach
Source: Front Public Health. 2025 Sep 12;13:1524215. doi: 10.3389/fpubh.2025.1524215 (PMC12463830; doi:10.3389/fpubh.2025.1524215)
Supplement: Supplementary file 1 [file Data_Sheet_1.ZIP › SEM模型数据/LetPub - Certificate 2024.pdf]

CERTIFICATE

OF ENGLISH LANGUAGE EDITING

Analysis of Factors Influencing Changes in Medical Behavior under the Context of DRG Payment Method Reform: A Structural Equation Modeling Approach

Objectives: This study aims to assess the understanding of policies, cognitive awareness, and medical behavior patterns among healthcare workers about the reform of the diagnosis-related group (DRG) payment method. A questionnaire survey was conducted to examine how policy comprehension and cognition influence changes in medical behavior, targeting medical personnel in a northern Chinese city.
Methods: An online survey was administered from November to December 2023, covering primary, secondary, and tertiary medical institutions in a city that had implemented DRG payments. The survey gathered demographic data and information on policy comprehension, medical behavior patterns, and policy cognition. Structural equation modeling was employed to analyze the relationships among these factors and their impact on shifts in medical ...

This document certifies that the manuscript listed above was copy edited for English language by LetPub, with regard to grammar, punctuation, spelling, and clarity. Documents receiving this certification should be regarded as having undergone professional editorial revision for English language before submission. However, the authors may accept or reject LetPub's suggestions and changes at their own discretion and LetPub does not have editorial control over the submitted documents. Submitted documents may have new text that was not provided to LetPub for review. Please use the verification link below to determine the validity of the submitted version.

November 1, 2024

Date of Revision

LetPub

LetPub is an author service brand owned and operated by Accdon LLC.
Tel: 1-781-202-9968 Email: info@accdon.com
Address: 400 Fifth Ave, Suite 530, Waltham, MA 02451, United States

This manuscript has been individually edited for grammar, punctuation, spelling, and clarity. You may verify the authenticity of this certificate on our website (https://www.letpub.com/editorial-certificate) at any time using this manuscript's unique code: PR_241024H468Y.

© 2024 Accdon, LLC.
