# Supplementary material for: Analysis of factors influencing changes in medical behavior under the context of DRG payment method reform: a structural equation modeling approach
Source: Front Public Health. 2025 Sep 12;13:1524215. doi: 10.3389/fpubh.2025.1524215 (PMC12463830; doi:10.3389/fpubh.2025.1524215)
Supplement: Supplementary file 1 [file Data_Sheet_1.ZIP › SEM模型数据/S1Questionnaire Information/Questionnaire questions on structural equation modeling.docx]

政策了解程度

K1、K2、K3

1、您是否了解DRG的概念

完全不了解 听说 大致了解 比较了解 完全了解

2、您是否了解DRG付费的原理和机制

完全不了解 听说 大致了解 比较了解 完全了解

3、您是否了解临沂市DRG付费整体结算方案

完全不了解 听说 大致了解 比较了解 完全了解

正向医疗行为

R1-R8

1. 缩短住院时间

非常同意 同意 一般 不同意 非常不同意

1. 减少药品使用种类

非常同意 同意 一般 不同意 非常不同意

1. 减少药物使用天数

非常同意 同意 一般 不同意 非常不同意

1. 减少使用辅助药物

非常同意 同意 一般 不同意 非常不同意

1. 减少使用预防性抗菌药

非常同意 同意 一般 不同意 非常不同意

1. 减少使用中成药或中药注射剂

非常同意 同意 一般 不同意 非常不同意

1. 增加使用基本药物

非常同意 同意 一般 不同意 非常不同意

1. 减少高值耗材使用

非常同意 同意 一般 不同意 非常不同意

负向医疗行为

F1-F4

1. 推诿可能超支的患者

非常同意 同意 一般 不同意 非常不同意

1. 降低出院标准（不恰当的提前出院）

非常同意 同意 一般 不同意 非常不同意

1. 分解住院

非常同意 同意 一般 不同意 非常不同意

1. 增加患者转诊

非常同意 同意 一般 不同意 非常不同意

政策正向认知

P1-P5

1. DRG支付方式能够降低医疗费用

非常同意 同意 一般 不同意 非常不同意

1. DRG支付方式能够提高医疗服务效率

非常同意 同意 一般 不同意 非常不同意

1. DRG支付方式能够提高医疗服务质量

非常同意 同意 一般 不同意 非常不同意

1. DRG支付方式有助于提高患者满意度

非常同意 同意 一般 不同意 非常不同意

1. DRG支付方式能规范医疗行为

非常同意 同意 一般 不同意 非常不同意

政策负向认知

N1-N3

1. DRG支付方式增加了医务人员的工作量

非常同意 同意 一般 不同意 非常不同意

1. DRG支付方式限制了医生对患者采取最合适的诊疗措施

非常同意 同意 一般 不同意 非常不同意

1. DRG支付方式会导致医生挑选患者

非常同意 同意 一般 不同意 非常不同意
