# Supplementary material for: Analysis of factors influencing changes in medical behavior under the context of DRG payment method reform: a structural equation modeling approach
Source: Front Public Health. 2025 Sep 12;13:1524215. doi: 10.3389/fpubh.2025.1524215 (PMC12463830; doi:10.3389/fpubh.2025.1524215)
Supplement: Supplementary file 1 [file Data_Sheet_1.ZIP › SEM模型数据/S2Model data/结构方程模型英文.AmosOutput]

结构方程模型英文.amw


#### C:\Users\jiang\Desktop\实验数据\模型修正数据\结构方程模型英文.amw

##### Analysis Summary

##### Date and Time

Date: 2024年11月2日

Time: 9:30:50

##### Title

结构方程模型英文: 2024年11月2日 9:30

##### Groups

##### Group number 1 (Group number 1)

##### Notes for Group (Group number 1)

The model is recursive.

Sample size = 675

##### Variable Summary (Group number 1)

##### Your model contains the following variables (Group number 1)

Observed, endogenous variables

F1

F2

F3

F4

P4

P1

P2

P5

P3

R8

R6

R5

R4

R3

R2

R1

k1

K3

K2

N1

N2

N3

R7

Unobserved, endogenous variables

PPP

PMB

LPU

NPP

NMB

Unobserved, exogenous variables

e25

e24

e23

e22

e17

e14

e15

e16

e18

e13

e11

e10

e9

e8

e7

e6

e1

e28

e27

e26

e2

e19

e20

e21

e4

e5

e12

e3

##### Variable counts (Group number 1)

|  |  |
| --- | --- |
| Number of variables in your model: | 56 |
| Number of observed variables: | 23 |
| Number of unobserved variables: | 33 |
| Number of exogenous variables: | 28 |
| Number of endogenous variables: | 28 |

##### Parameter Summary (Group number 1)

|  | Weights | Covariances | Variances | Means | Intercepts | Total |
| --- | --- | --- | --- | --- | --- | --- |
| Fixed | 33 | 0 | 0 | 0 | 0 | 33 |
| Labeled | 0 | 0 | 0 | 0 | 0 | 0 |
| Unlabeled | 26 | 7 | 28 | 0 | 0 | 61 |
| Total | 59 | 7 | 28 | 0 | 0 | 94 |

##### Assessment of normality (Group number 1)

| Variable | min | max | skew | c.r. | kurtosis | c.r. |
| --- | --- | --- | --- | --- | --- | --- |
| R7 | 1.000 | 5.000 | .919 | 9.752 | 1.063 | 5.638 |
| N3 | 1.000 | 5.000 | .395 | 4.187 | -.566 | -3.001 |
| N2 | 1.000 | 5.000 | .371 | 3.931 | -.546 | -2.895 |
| N1 | 1.000 | 5.000 | .151 | 1.601 | -.847 | -4.493 |
| K2 | 1.000 | 5.000 | .038 | .408 | -.477 | -2.532 |
| K3 | 1.000 | 5.000 | -.030 | -.321 | -.354 | -1.877 |
| k1 | 1.000 | 5.000 | -.013 | -.141 | -.610 | -3.233 |
| R1 | 1.000 | 5.000 | .860 | 9.116 | 1.524 | 8.082 |
| R2 | 1.000 | 5.000 | .827 | 8.767 | .794 | 4.211 |
| R3 | 1.000 | 5.000 | .855 | 9.066 | 1.077 | 5.711 |
| R4 | 1.000 | 5.000 | .979 | 10.387 | 1.524 | 8.081 |
| R5 | 1.000 | 5.000 | .910 | 9.656 | 1.356 | 7.190 |
| R6 | 1.000 | 5.000 | .712 | 7.548 | .319 | 1.693 |
| R8 | 1.000 | 5.000 | .995 | 10.553 | 1.997 | 10.590 |
| P3 | 1.000 | 5.000 | .337 | 3.570 | -.686 | -3.638 |
| P5 | 1.000 | 5.000 | .638 | 6.767 | .109 | .577 |
| P2 | 1.000 | 5.000 | .420 | 4.453 | -.558 | -2.962 |
| P1 | 1.000 | 5.000 | .775 | 8.220 | .346 | 1.835 |
| P4 | 1.000 | 5.000 | .097 | 1.028 | -.796 | -4.220 |
| F4 | 1.000 | 5.000 | -.084 | -.896 | -.982 | -5.206 |
| F3 | 1.000 | 5.000 | -.385 | -4.085 | -.810 | -4.297 |
| F2 | 1.000 | 5.000 | -.338 | -3.584 | -.830 | -4.403 |
| F1 | 1.000 | 5.000 | -.428 | -4.538 | -.779 | -4.132 |
| Multivariate |  |  |  |  | 266.600 | 102.125 |

##### Observations farthest from the centroid (Mahalanobis distance) (Group number 1)

| Observation number | Mahalanobis d-squared | p1 | p2 |
| --- | --- | --- | --- |
| 398 | 131.937 | .000 | .000 |
| 11 | 103.109 | .000 | .000 |
| 637 | 92.120 | .000 | .000 |
| 532 | 91.211 | .000 | .000 |
| 243 | 89.769 | .000 | .000 |
| 345 | 85.060 | .000 | .000 |
| 395 | 82.868 | .000 | .000 |
| 528 | 81.873 | .000 | .000 |
| 653 | 81.704 | .000 | .000 |
| 349 | 80.750 | .000 | .000 |
| 452 | 79.566 | .000 | .000 |
| 207 | 78.214 | .000 | .000 |
| 150 | 77.357 | .000 | .000 |
| 402 | 73.851 | .000 | .000 |
| 160 | 73.311 | .000 | .000 |
| 111 | 71.772 | .000 | .000 |
| 435 | 71.268 | .000 | .000 |
| 495 | 71.231 | .000 | .000 |
| 315 | 70.107 | .000 | .000 |
| 358 | 69.485 | .000 | .000 |
| 614 | 68.513 | .000 | .000 |
| 213 | 68.298 | .000 | .000 |
| 663 | 67.872 | .000 | .000 |
| 224 | 67.575 | .000 | .000 |
| 475 | 66.923 | .000 | .000 |
| 428 | 66.827 | .000 | .000 |
| 297 | 65.371 | .000 | .000 |
| 162 | 64.601 | .000 | .000 |
| 486 | 64.190 | .000 | .000 |
| 468 | 61.922 | .000 | .000 |
| 330 | 61.839 | .000 | .000 |
| 523 | 61.364 | .000 | .000 |
| 620 | 59.582 | .000 | .000 |
| 616 | 59.227 | .000 | .000 |
| 543 | 58.983 | .000 | .000 |
| 69 | 58.832 | .000 | .000 |
| 423 | 58.738 | .000 | .000 |
| 195 | 58.514 | .000 | .000 |
| 539 | 58.426 | .000 | .000 |
| 107 | 58.225 | .000 | .000 |
| 265 | 57.896 | .000 | .000 |
| 161 | 55.450 | .000 | .000 |
| 356 | 54.910 | .000 | .000 |
| 436 | 54.277 | .000 | .000 |
| 426 | 53.836 | .000 | .000 |
| 665 | 53.070 | .000 | .000 |
| 514 | 52.486 | .000 | .000 |
| 269 | 52.139 | .000 | .000 |
| 88 | 52.111 | .000 | .000 |
| 310 | 51.305 | .001 | .000 |
| 639 | 51.111 | .001 | .000 |
| 672 | 50.098 | .001 | .000 |
| 619 | 49.980 | .001 | .000 |
| 526 | 49.921 | .001 | .000 |
| 112 | 49.753 | .001 | .000 |
| 143 | 49.529 | .001 | .000 |
| 227 | 49.331 | .001 | .000 |
| 326 | 49.203 | .001 | .000 |
| 337 | 49.165 | .001 | .000 |
| 490 | 48.829 | .001 | .000 |
| 21 | 47.523 | .002 | .000 |
| 325 | 46.917 | .002 | .000 |
| 284 | 46.807 | .002 | .000 |
| 152 | 46.427 | .003 | .000 |
| 89 | 46.277 | .003 | .000 |
| 38 | 45.771 | .003 | .000 |
| 448 | 45.539 | .003 | .000 |
| 286 | 45.498 | .003 | .000 |
| 513 | 45.406 | .004 | .000 |
| 460 | 45.325 | .004 | .000 |
| 491 | 45.265 | .004 | .000 |
| 657 | 44.993 | .004 | .000 |
| 323 | 44.973 | .004 | .000 |
| 542 | 44.894 | .004 | .000 |
| 67 | 44.791 | .004 | .000 |
| 305 | 44.590 | .004 | .000 |
| 485 | 44.251 | .005 | .000 |
| 566 | 44.205 | .005 | .000 |
| 23 | 43.953 | .005 | .000 |
| 575 | 43.881 | .005 | .000 |
| 178 | 43.736 | .006 | .000 |
| 443 | 43.558 | .006 | .000 |
| 628 | 43.178 | .007 | .000 |
| 613 | 42.249 | .008 | .000 |
| 461 | 41.630 | .010 | .000 |
| 584 | 41.485 | .010 | .000 |
| 595 | 41.410 | .011 | .000 |
| 384 | 41.367 | .011 | .000 |
| 81 | 41.075 | .012 | .000 |
| 536 | 41.019 | .012 | .000 |
| 170 | 40.764 | .013 | .000 |
| 174 | 40.342 | .014 | .000 |
| 594 | 40.306 | .014 | .000 |
| 320 | 40.106 | .015 | .000 |
| 631 | 40.047 | .015 | .000 |
| 430 | 39.522 | .017 | .000 |
| 79 | 39.273 | .019 | .000 |
| 205 | 39.273 | .019 | .000 |
| 634 | 39.059 | .020 | .000 |
| 133 | 38.504 | .022 | .000 |

##### Sample Moments (Group number 1)

##### Sample Covariances (Group number 1)

|  | R7 | N3 | N2 | N1 | K2 | K3 | k1 | R1 | R2 | R3 | R4 | R5 | R6 | R8 | P3 | P5 | P2 | P1 | P4 | F4 | F3 | F2 | F1 |
| --- | --- | --- | --- | --- | --- | --- | --- | --- | --- | --- | --- | --- | --- | --- | --- | --- | --- | --- | --- | --- | --- | --- | --- |
| R7 | .753 |
| N3 | .152 | 1.149 |
| N2 | .111 | .551 | 1.113 |
| N1 | .078 | .535 | .630 | 1.202 |
| K2 | -.103 | -.006 | .013 | .040 | .798 |
| K3 | -.109 | .022 | .034 | .093 | .689 | .834 |
| k1 | -.082 | -.040 | .028 | .043 | .597 | .571 | .748 |
| R1 | .319 | .144 | .144 | .147 | -.062 | -.071 | -.047 | .646 |
| R2 | .390 | .108 | .084 | .091 | -.065 | -.078 | -.064 | .438 | .769 |
| R3 | .379 | .127 | .088 | .106 | -.052 | -.060 | -.041 | .431 | .649 | .743 |
| R4 | .404 | .123 | .064 | .050 | -.090 | -.084 | -.075 | .398 | .554 | .577 | .718 |
| R5 | .413 | .115 | .126 | .061 | -.048 | -.057 | -.035 | .375 | .511 | .532 | .532 | .694 |
| R6 | .373 | .224 | .193 | .181 | -.050 | -.069 | -.061 | .421 | .529 | .523 | .531 | .520 | .908 |
| R8 | .390 | .143 | .126 | .134 | -.095 | -.084 | -.098 | .300 | .357 | .342 | .379 | .339 | .320 | .637 |
| P3 | .293 | -.018 | -.016 | .057 | -.011 | -.030 | .012 | .287 | .346 | .363 | .296 | .283 | .309 | .292 | 1.300 |
| P5 | .307 | .007 | .007 | .006 | -.034 | -.068 | -.013 | .256 | .369 | .363 | .336 | .333 | .346 | .281 | .816 | 1.048 |
| P2 | .315 | -.012 | .016 | .035 | -.028 | -.053 | -.003 | .266 | .352 | .360 | .305 | .309 | .313 | .258 | 1.096 | .810 | 1.238 |
| P1 | .319 | .022 | .050 | .062 | -.065 | -.066 | -.055 | .311 | .336 | .336 | .321 | .315 | .294 | .249 | .697 | .661 | .716 | .973 |
| P4 | .243 | -.024 | .005 | .056 | .016 | -.016 | .014 | .224 | .307 | .314 | .242 | .262 | .320 | .224 | .981 | .809 | .937 | .630 | 1.292 |
| F4 | -.034 | .604 | .447 | .416 | .094 | .111 | .020 | .074 | -.003 | -.002 | .002 | -.012 | .105 | .037 | -.063 | -.048 | -.035 | -.034 | -.005 | 1.265 |
| F3 | -.121 | .427 | .364 | .446 | .172 | .208 | .129 | -.018 | -.035 | -.054 | -.089 | -.070 | -.026 | -.051 | .030 | -.021 | -.008 | -.030 | .123 | .813 | 1.285 |
| F2 | -.120 | .534 | .387 | .480 | .108 | .141 | .045 | -.011 | -.077 | -.078 | -.140 | -.093 | .011 | -.044 | -.120 | -.117 | -.158 | -.116 | -.045 | .753 | .790 | 1.210 |
| F1 | -.132 | .564 | .394 | .514 | .138 | .160 | .073 | -.013 | -.105 | -.113 | -.141 | -.155 | -.031 | -.076 | -.248 | -.257 | -.237 | -.184 | -.185 | .750 | .717 | .899 | 1.336 |

Condition number = 65.746

Eigenvalues

6.268 4.785 2.502 1.850 1.128 .678 .635 .529 .500 .460 .439 .405 .346 .333 .318 .284 .273 .212 .195 .161 .147 .115 .095

Determinant of sample covariance matrix = .000

##### Sample Correlations (Group number 1)

|  | R7 | N3 | N2 | N1 | K2 | K3 | k1 | R1 | R2 | R3 | R4 | R5 | R6 | R8 | P3 | P5 | P2 | P1 | P4 | F4 | F3 | F2 | F1 |
| --- | --- | --- | --- | --- | --- | --- | --- | --- | --- | --- | --- | --- | --- | --- | --- | --- | --- | --- | --- | --- | --- | --- | --- |
| R7 | 1.000 |
| N3 | .163 | 1.000 |
| N2 | .121 | .487 | 1.000 |
| N1 | .082 | .455 | .544 | 1.000 |
| K2 | -.133 | -.007 | .014 | .041 | 1.000 |
| K3 | -.138 | .022 | .035 | .093 | .844 | 1.000 |
| k1 | -.109 | -.043 | .030 | .045 | .772 | .723 | 1.000 |
| R1 | .457 | .167 | .170 | .167 | -.087 | -.096 | -.068 | 1.000 |
| R2 | .513 | .114 | .090 | .095 | -.083 | -.097 | -.084 | .621 | 1.000 |
| R3 | .507 | .137 | .096 | .112 | -.068 | -.076 | -.055 | .622 | .858 | 1.000 |
| R4 | .550 | .135 | .072 | .053 | -.119 | -.108 | -.102 | .584 | .746 | .790 | 1.000 |
| R5 | .572 | .128 | .143 | .066 | -.065 | -.075 | -.049 | .560 | .699 | .741 | .754 | 1.000 |
| R6 | .451 | .219 | .192 | .174 | -.058 | -.079 | -.074 | .549 | .634 | .637 | .658 | .655 | 1.000 |
| R8 | .563 | .167 | .150 | .153 | -.134 | -.116 | -.142 | .467 | .511 | .497 | .561 | .510 | .421 | 1.000 |
| P3 | .297 | -.015 | -.014 | .046 | -.011 | -.029 | .012 | .313 | .346 | .370 | .306 | .298 | .284 | .321 | 1.000 |
| P5 | .346 | .007 | .007 | .005 | -.037 | -.073 | -.015 | .311 | .411 | .411 | .388 | .390 | .354 | .344 | .699 | 1.000 |
| P2 | .327 | -.010 | .014 | .029 | -.028 | -.052 | -.003 | .297 | .361 | .375 | .323 | .333 | .295 | .291 | .864 | .711 | 1.000 |
| P1 | .372 | .020 | .048 | .057 | -.074 | -.073 | -.064 | .392 | .389 | .396 | .384 | .384 | .313 | .316 | .620 | .654 | .653 | 1.000 |
| P4 | .247 | -.019 | .005 | .045 | .016 | -.015 | .014 | .245 | .308 | .321 | .251 | .277 | .295 | .247 | .757 | .695 | .741 | .562 | 1.000 |
| F4 | -.035 | .501 | .377 | .337 | .093 | .108 | .021 | .082 | -.003 | -.002 | .002 | -.013 | .098 | .041 | -.049 | -.042 | -.028 | -.031 | -.004 | 1.000 |
| F3 | -.123 | .351 | .304 | .359 | .170 | .201 | .131 | -.020 | -.035 | -.056 | -.093 | -.074 | -.024 | -.056 | .023 | -.018 | -.006 | -.027 | .096 | .638 | 1.000 |
| F2 | -.126 | .453 | .334 | .398 | .110 | .140 | .048 | -.013 | -.079 | -.082 | -.150 | -.101 | .011 | -.051 | -.096 | -.104 | -.129 | -.107 | -.036 | .609 | .633 | 1.000 |
| F1 | -.132 | .455 | .323 | .405 | .134 | .152 | .073 | -.014 | -.104 | -.113 | -.144 | -.161 | -.028 | -.082 | -.188 | -.217 | -.184 | -.162 | -.140 | .577 | .547 | .707 | 1.000 |

Condition number = 57.797

Eigenvalues

6.870 3.973 2.661 2.147 1.000 .720 .585 .534 .511 .461 .441 .411 .393 .341 .303 .288 .274 .257 .233 .207 .142 .131 .119

##### Models

##### Default model (Default model)

##### Notes for Model (Default model)

##### Computation of degrees of freedom (Default model)

|  |  |
| --- | --- |
| Number of distinct sample moments: | 276 |
| Number of distinct parameters to be estimated: | 61 |
| Degrees of freedom (276 - 61): | 215 |

##### Result (Default model)

Minimum was achieved

Chi-square = 637.638

Degrees of freedom = 215

Probability level = .000

##### Group number 1 (Group number 1 - Default model)

##### Estimates (Group number 1 - Default model)

##### Scalar Estimates (Group number 1 - Default model)

##### Maximum Likelihood Estimates

##### Regression Weights: (Group number 1 - Default model)

|  |  |  | Estimate | S.E. | C.R. | P | Label |
| --- | --- | --- | --- | --- | --- | --- | --- |
| PPP | <--- | LPU | -.028 | .040 | -.689 | .491 | par\_19 |
| NPP | <--- | LPU | .041 | .051 | .799 | .424 | par\_20 |
| PMB | <--- | LPU | -.124 | .040 | -3.111 | .002 | par\_17 |
| NMB | <--- | LPU | .182 | .049 | 3.746 | \*\*\* | par\_18 |
| PMB | <--- | PPP | .490 | .046 | 10.756 | \*\*\* | par\_21 |
| NMB | <--- | PPP | -.212 | .051 | -4.146 | \*\*\* | par\_22 |
| PMB | <--- | NPP | .155 | .040 | 3.857 | \*\*\* | par\_23 |
| NMB | <--- | NPP | .821 | .067 | 12.282 | \*\*\* | par\_33 |
| P4 | <--- | PPP | 1.337 | .068 | 19.623 | \*\*\* | par\_2 |
| P1 | <--- | PPP | 1.000 |  |
| P2 | <--- | PPP | 1.514 | .068 | 22.424 | \*\*\* | par\_3 |
| P5 | <--- | PPP | 1.146 | .053 | 21.769 | \*\*\* | par\_4 |
| R8 | <--- | PMB | .648 | .036 | 17.957 | \*\*\* | par\_7 |
| R6 | <--- | PMB | .945 | .039 | 24.000 | \*\*\* | par\_8 |
| R5 | <--- | PMB | .929 | .031 | 29.528 | \*\*\* | par\_9 |
| R4 | <--- | PMB | 1.000 |  |
| R3 | <--- | PMB | .996 | .031 | 31.905 | \*\*\* | par\_10 |
| R2 | <--- | PMB | .977 | .033 | 29.379 | \*\*\* | par\_11 |
| R1 | <--- | PMB | .760 | .036 | 20.882 | \*\*\* | par\_12 |
| k1 | <--- | LPU | 1.000 |  |
| K3 | <--- | LPU | 1.156 | .041 | 27.952 | \*\*\* | par\_15 |
| N1 | <--- | NPP | 1.000 |  |
| N2 | <--- | NPP | .952 | .063 | 15.039 | \*\*\* | par\_16 |
| F1 | <--- | NMB | 1.000 |  |
| N3 | <--- | NPP | .990 | .073 | 13.511 | \*\*\* | par\_24 |
| R7 | <--- | PMB | .719 | .039 | 18.390 | \*\*\* | par\_26 |
| F4 | <--- | NMB | .857 | .045 | 19.012 | \*\*\* | par\_28 |
| F3 | <--- | NMB | .850 | .046 | 18.415 | \*\*\* | par\_29 |
| F2 | <--- | NMB | 1.005 | .043 | 23.484 | \*\*\* | par\_30 |
| K2 | <--- | LPU | 1.206 | .041 | 29.359 | \*\*\* | par\_31 |
| P3 | <--- | PPP | 1.540 | .070 | 22.082 | \*\*\* | par\_32 |

##### Standardized Regression Weights: (Group number 1 - Default model)

|  |  |  | Estimate |
| --- | --- | --- | --- |
| PPP | <--- | LPU | -.028 |
| NPP | <--- | LPU | .037 |
| PMB | <--- | LPU | -.115 |
| NMB | <--- | LPU | .136 |
| PMB | <--- | PPP | .444 |
| NMB | <--- | PPP | -.154 |
| PMB | <--- | NPP | .159 |
| NMB | <--- | NPP | .674 |
| P4 | <--- | PPP | .805 |
| P1 | <--- | PPP | .694 |
| P2 | <--- | PPP | .931 |
| P5 | <--- | PPP | .766 |
| R8 | <--- | PMB | .613 |
| R6 | <--- | PMB | .748 |
| R5 | <--- | PMB | .842 |
| R4 | <--- | PMB | .891 |
| R3 | <--- | PMB | .872 |
| R2 | <--- | PMB | .841 |
| R1 | <--- | PMB | .714 |
| k1 | <--- | LPU | .813 |
| K3 | <--- | LPU | .890 |
| N1 | <--- | NPP | .706 |
| N2 | <--- | NPP | .698 |
| F1 | <--- | NMB | .815 |
| N3 | <--- | NPP | .715 |
| R7 | <--- | PMB | .626 |
| F4 | <--- | NMB | .718 |
| F3 | <--- | NMB | .706 |
| F2 | <--- | NMB | .861 |
| K2 | <--- | LPU | .949 |
| P3 | <--- | PPP | .924 |

##### Covariances: (Group number 1 - Default model)

|  |  |  | Estimate | S.E. | C.R. | P | Label |
| --- | --- | --- | --- | --- | --- | --- | --- |
| e4 | <--> | e5 | .010 | .024 | .414 | .679 | par\_25 |
| e23 | <--> | e22 | .166 | .032 | 5.282 | \*\*\* | par\_1 |
| e14 | <--> | e18 | .123 | .020 | 6.229 | \*\*\* | par\_5 |
| e17 | <--> | e18 | .090 | .019 | 4.717 | \*\*\* | par\_6 |
| e8 | <--> | e7 | .094 | .011 | 8.232 | \*\*\* | par\_13 |
| e9 | <--> | e6 | -.035 | .011 | -3.255 | .001 | par\_14 |
| e13 | <--> | e12 | .125 | .018 | 6.872 | \*\*\* | par\_27 |

##### Correlations: (Group number 1 - Default model)

|  |  |  | Estimate |
| --- | --- | --- | --- |
| e4 | <--> | e5 | .019 |
| e23 | <--> | e22 | .265 |
| e14 | <--> | e18 | .264 |
| e17 | <--> | e18 | .203 |
| e8 | <--> | e7 | .471 |
| e9 | <--> | e6 | -.162 |
| e13 | <--> | e12 | .292 |

##### Variances: (Group number 1 - Default model)

|  |  |  | Estimate | S.E. | C.R. | P | Label |
| --- | --- | --- | --- | --- | --- | --- | --- |
| e2 |  |  | .494 | .039 | 12.562 | \*\*\* | par\_34 |
| e4 |  |  | .598 | .066 | 9.068 | \*\*\* | par\_35 |
| e5 |  |  | .468 | .046 | 10.080 | \*\*\* | par\_36 |
| e1 |  |  | .433 | .031 | 13.899 | \*\*\* | par\_37 |
| e3 |  |  | .443 | .046 | 9.524 | \*\*\* | par\_38 |
| e25 |  |  | .449 | .035 | 12.657 | \*\*\* | par\_39 |
| e24 |  |  | .314 | .030 | 10.420 | \*\*\* | par\_40 |
| e23 |  |  | .644 | .042 | 15.316 | \*\*\* | par\_41 |
| e22 |  |  | .613 | .041 | 15.057 | \*\*\* | par\_42 |
| e17 |  |  | .454 | .028 | 16.014 | \*\*\* | par\_43 |
| e14 |  |  | .504 | .029 | 17.187 | \*\*\* | par\_44 |
| e15 |  |  | .164 | .016 | 10.015 | \*\*\* | par\_45 |
| e16 |  |  | .189 | .018 | 10.764 | \*\*\* | par\_46 |
| e18 |  |  | .433 | .026 | 16.580 | \*\*\* | par\_47 |
| e13 |  |  | .398 | .023 | 17.552 | \*\*\* | par\_48 |
| e11 |  |  | .400 | .024 | 16.679 | \*\*\* | par\_49 |
| e10 |  |  | .203 | .014 | 14.957 | \*\*\* | par\_50 |
| e9 |  |  | .148 | .012 | 12.505 | \*\*\* | par\_51 |
| e8 |  |  | .178 | .013 | 13.940 | \*\*\* | par\_52 |
| e7 |  |  | .225 | .015 | 14.796 | \*\*\* | par\_53 |
| e6 |  |  | .317 | .019 | 16.329 | \*\*\* | par\_54 |
| e28 |  |  | .254 | .016 | 15.489 | \*\*\* | par\_55 |
| e27 |  |  | .079 | .014 | 5.800 | \*\*\* | par\_56 |
| e26 |  |  | .173 | .015 | 11.375 | \*\*\* | par\_57 |
| e19 |  |  | .603 | .047 | 12.721 | \*\*\* | par\_58 |
| e20 |  |  | .571 | .043 | 13.127 | \*\*\* | par\_59 |
| e21 |  |  | .562 | .046 | 12.273 | \*\*\* | par\_60 |
| e12 |  |  | .458 | .026 | 17.485 | \*\*\* | par\_61 |

##### Squared Multiple Correlations: (Group number 1 - Default model)

|  |  |  | Estimate |
| --- | --- | --- | --- |
| LPU |  |  | .000 |
| NPP |  |  | .001 |
| PPP |  |  | .001 |
| NMB |  |  | .501 |
| PMB |  |  | .240 |
| R7 |  |  | .392 |
| N3 |  |  | .511 |
| N2 |  |  | .488 |
| N1 |  |  | .499 |
| K2 |  |  | .901 |
| K3 |  |  | .792 |
| k1 |  |  | .660 |
| R1 |  |  | .509 |
| R2 |  |  | .707 |
| R3 |  |  | .761 |
| R4 |  |  | .794 |
| R5 |  |  | .708 |
| R6 |  |  | .560 |
| R8 |  |  | .375 |
| P3 |  |  | .855 |
| P5 |  |  | .587 |
| P2 |  |  | .867 |
| P1 |  |  | .481 |
| P4 |  |  | .648 |
| F4 |  |  | .516 |
| F3 |  |  | .499 |
| F2 |  |  | .741 |
| F1 |  |  | .664 |

##### Matrices (Group number 1 - Default model)

##### Implied (for all variables) Covariances (Group number 1 - Default model)

|  | LPU | NPP | PPP | NMB | PMB | R7 | N3 | N2 | N1 | K2 | K3 | k1 | R1 | R2 | R3 | R4 | R5 | R6 | R8 | P3 | P5 | P2 | P1 | P4 | F4 | F3 | F2 | F1 |
| --- | --- | --- | --- | --- | --- | --- | --- | --- | --- | --- | --- | --- | --- | --- | --- | --- | --- | --- | --- | --- | --- | --- | --- | --- | --- | --- | --- | --- |
| LPU | .494 |
| NPP | .020 | .599 |
| PPP | -.014 | .010 | .468 |
| NMB | .110 | .494 | -.094 | .888 |
| PMB | -.065 | .095 | .232 | .017 | .570 |
| R7 | -.047 | .068 | .167 | .012 | .410 | .753 |
| N3 | .020 | .593 | .009 | .489 | .094 | .068 | 1.149 |
| N2 | .019 | .570 | .009 | .470 | .091 | .065 | .565 | 1.113 |
| N1 | .020 | .599 | .010 | .494 | .095 | .068 | .593 | .570 | 1.202 |
| K2 | .596 | .024 | -.016 | .132 | -.078 | -.056 | .024 | .023 | .024 | .798 |
| K3 | .571 | .023 | -.016 | .127 | -.075 | -.054 | .023 | .022 | .023 | .689 | .834 |
| k1 | .494 | .020 | -.014 | .110 | -.065 | -.047 | .020 | .019 | .020 | .596 | .571 | .748 |
| R1 | -.049 | .072 | .177 | .013 | .433 | .312 | .072 | .069 | .072 | -.059 | -.057 | -.049 | .646 |
| R2 | -.063 | .093 | .227 | .017 | .556 | .400 | .092 | .088 | .093 | -.076 | -.073 | -.063 | .423 | .769 |
| R3 | -.064 | .095 | .232 | .017 | .568 | .408 | .094 | .090 | .095 | -.078 | -.075 | -.064 | .432 | .649 | .743 |
| R4 | -.065 | .095 | .232 | .017 | .570 | .410 | .094 | .091 | .095 | -.078 | -.075 | -.065 | .398 | .556 | .568 | .718 |
| R5 | -.060 | .088 | .216 | .016 | .529 | .381 | .087 | .084 | .088 | -.072 | -.069 | -.060 | .402 | .517 | .527 | .529 | .694 |
| R6 | -.061 | .090 | .220 | .016 | .538 | .387 | .089 | .086 | .090 | -.074 | -.071 | -.061 | .409 | .526 | .536 | .538 | .500 | .908 |
| R8 | -.042 | .062 | .151 | .011 | .369 | .390 | .061 | .059 | .062 | -.051 | -.048 | -.042 | .281 | .360 | .368 | .369 | .343 | .349 | .637 |
| P3 | -.021 | .015 | .721 | -.144 | .358 | .257 | .015 | .014 | .015 | -.025 | -.024 | -.021 | .272 | .350 | .357 | .358 | .332 | .338 | .232 | 1.300 |
| P5 | -.016 | .011 | .537 | -.107 | .266 | .192 | .011 | .010 | .011 | -.019 | -.018 | -.016 | .202 | .260 | .265 | .266 | .247 | .252 | .172 | .826 | 1.048 |
| P2 | -.021 | .014 | .709 | -.142 | .352 | .253 | .014 | .014 | .014 | -.025 | -.024 | -.021 | .267 | .344 | .351 | .352 | .327 | .332 | .228 | 1.092 | .813 | 1.238 |
| P1 | -.014 | .010 | .468 | -.094 | .232 | .167 | .009 | .009 | .010 | -.016 | -.016 | -.014 | .177 | .227 | .232 | .232 | .216 | .220 | .151 | .721 | .660 | .709 | .973 |
| P4 | -.018 | .013 | .626 | -.125 | .311 | .224 | .013 | .012 | .013 | -.022 | -.021 | -.018 | .236 | .304 | .310 | .311 | .289 | .294 | .201 | .965 | .808 | .948 | .626 | 1.292 |
| F4 | .094 | .423 | -.080 | .761 | .015 | .011 | .419 | .403 | .423 | .113 | .109 | .094 | .011 | .014 | .015 | .015 | .014 | .014 | .009 | -.124 | -.092 | -.122 | -.080 | -.108 | 1.265 |
| F3 | .093 | .419 | -.080 | .754 | .015 | .010 | .415 | .399 | .419 | .112 | .108 | .093 | .011 | .014 | .014 | .015 | .014 | .014 | .009 | -.123 | -.091 | -.121 | -.080 | -.107 | .813 | 1.285 |
| F2 | .110 | .496 | -.094 | .892 | .017 | .012 | .491 | .472 | .496 | .133 | .127 | .110 | .013 | .017 | .017 | .017 | .016 | .016 | .011 | -.145 | -.108 | -.143 | -.094 | -.126 | .764 | .758 | 1.210 |
| F1 | .110 | .494 | -.094 | .888 | .017 | .012 | .489 | .470 | .494 | .132 | .127 | .110 | .013 | .017 | .017 | .017 | .016 | .016 | .011 | -.144 | -.107 | -.142 | -.094 | -.125 | .761 | .754 | .892 | 1.336 |

##### Implied (for all variables) Correlations (Group number 1 - Default model)

|  | LPU | NPP | PPP | NMB | PMB | R7 | N3 | N2 | N1 | K2 | K3 | k1 | R1 | R2 | R3 | R4 | R5 | R6 | R8 | P3 | P5 | P2 | P1 | P4 | F4 | F3 | F2 | F1 |
| --- | --- | --- | --- | --- | --- | --- | --- | --- | --- | --- | --- | --- | --- | --- | --- | --- | --- | --- | --- | --- | --- | --- | --- | --- | --- | --- | --- | --- |
| LPU | 1.000 |
| NPP | .037 | 1.000 |
| PPP | -.028 | .018 | 1.000 |
| NMB | .165 | .677 | -.145 | 1.000 |
| PMB | -.122 | .163 | .450 | .024 | 1.000 |
| R7 | -.076 | .102 | .282 | .015 | .626 | 1.000 |
| N3 | .026 | .715 | .013 | .484 | .116 | .073 | 1.000 |
| N2 | .026 | .698 | .013 | .472 | .114 | .071 | .499 | 1.000 |
| N1 | .026 | .706 | .013 | .478 | .115 | .072 | .505 | .493 | 1.000 |
| K2 | .949 | .035 | -.027 | .157 | -.116 | -.072 | .025 | .024 | .025 | 1.000 |
| K3 | .890 | .033 | -.025 | .147 | -.108 | -.068 | .023 | .023 | .023 | .845 | 1.000 |
| k1 | .813 | .030 | -.023 | .134 | -.099 | -.062 | .021 | .021 | .021 | .771 | .723 | 1.000 |
| R1 | -.087 | .116 | .321 | .017 | .714 | .447 | .083 | .081 | .082 | -.083 | -.077 | -.071 | 1.000 |
| R2 | -.102 | .137 | .378 | .020 | .841 | .526 | .098 | .096 | .097 | -.097 | -.091 | -.083 | .600 | 1.000 |
| R3 | -.106 | .142 | .393 | .021 | .872 | .546 | .102 | .099 | .100 | -.101 | -.095 | -.086 | .623 | .858 | 1.000 |
| R4 | -.109 | .145 | .401 | .021 | .891 | .558 | .104 | .101 | .102 | -.103 | -.097 | -.088 | .584 | .749 | .777 | 1.000 |
| R5 | -.103 | .137 | .379 | .020 | .842 | .527 | .098 | .096 | .097 | -.097 | -.091 | -.083 | .601 | .708 | .734 | .750 | 1.000 |
| R6 | -.091 | .122 | .337 | .018 | .748 | .468 | .087 | .085 | .086 | -.087 | -.081 | -.074 | .534 | .629 | .653 | .667 | .630 | 1.000 |
| R8 | -.075 | .100 | .276 | .015 | .613 | .563 | .071 | .070 | .070 | -.071 | -.066 | -.061 | .437 | .515 | .534 | .546 | .516 | .458 | 1.000 |
| P3 | -.026 | .017 | .924 | -.134 | .416 | .260 | .012 | .012 | .012 | -.025 | -.023 | -.021 | .297 | .350 | .363 | .371 | .350 | .311 | .255 | 1.000 |
| P5 | -.022 | .014 | .766 | -.111 | .345 | .216 | .010 | .010 | .010 | -.021 | -.019 | -.018 | .246 | .290 | .301 | .307 | .290 | .258 | .211 | .708 | 1.000 |
| P2 | -.026 | .017 | .931 | -.135 | .419 | .262 | .012 | .012 | .012 | -.025 | -.023 | -.021 | .299 | .352 | .366 | .373 | .353 | .313 | .257 | .861 | .713 | 1.000 |
| P1 | -.020 | .012 | .694 | -.101 | .312 | .195 | .009 | .009 | .009 | -.019 | -.017 | -.016 | .223 | .263 | .272 | .278 | .263 | .234 | .191 | .641 | .654 | .646 | 1.000 |
| P4 | -.023 | .014 | .805 | -.117 | .362 | .227 | .010 | .010 | .010 | -.022 | -.020 | -.019 | .259 | .305 | .316 | .323 | .305 | .271 | .222 | .744 | .694 | .750 | .559 | 1.000 |
| F4 | .119 | .486 | -.104 | .718 | .017 | .011 | .347 | .339 | .343 | .113 | .106 | .097 | .012 | .015 | .015 | .015 | .015 | .013 | .011 | -.097 | -.080 | -.097 | -.072 | -.084 | 1.000 |
| F3 | .117 | .478 | -.103 | .706 | .017 | .011 | .342 | .334 | .337 | .111 | .104 | .095 | .012 | .014 | .015 | .015 | .014 | .013 | .010 | -.095 | -.079 | -.096 | -.071 | -.083 | .638 | 1.000 |
| F2 | .142 | .582 | -.125 | .861 | .021 | .013 | .416 | .407 | .411 | .135 | .127 | .116 | .015 | .017 | .018 | .018 | .017 | .015 | .013 | -.116 | -.096 | -.117 | -.087 | -.101 | .618 | .608 | 1.000 |
| F1 | .135 | .552 | -.119 | .815 | .020 | .012 | .394 | .385 | .389 | .128 | .120 | .110 | .014 | .016 | .017 | .017 | .016 | .015 | .012 | -.110 | -.091 | -.110 | -.082 | -.095 | .585 | .576 | .701 | 1.000 |

##### Implied Covariances (Group number 1 - Default model)

|  | R7 | N3 | N2 | N1 | K2 | K3 | k1 | R1 | R2 | R3 | R4 | R5 | R6 | R8 | P3 | P5 | P2 | P1 | P4 | F4 | F3 | F2 | F1 |
| --- | --- | --- | --- | --- | --- | --- | --- | --- | --- | --- | --- | --- | --- | --- | --- | --- | --- | --- | --- | --- | --- | --- | --- |
| R7 | .753 |
| N3 | .068 | 1.149 |
| N2 | .065 | .565 | 1.113 |
| N1 | .068 | .593 | .570 | 1.202 |
| K2 | -.056 | .024 | .023 | .024 | .798 |
| K3 | -.054 | .023 | .022 | .023 | .689 | .834 |
| k1 | -.047 | .020 | .019 | .020 | .596 | .571 | .748 |
| R1 | .312 | .072 | .069 | .072 | -.059 | -.057 | -.049 | .646 |
| R2 | .400 | .092 | .088 | .093 | -.076 | -.073 | -.063 | .423 | .769 |
| R3 | .408 | .094 | .090 | .095 | -.078 | -.075 | -.064 | .432 | .649 | .743 |
| R4 | .410 | .094 | .091 | .095 | -.078 | -.075 | -.065 | .398 | .556 | .568 | .718 |
| R5 | .381 | .087 | .084 | .088 | -.072 | -.069 | -.060 | .402 | .517 | .527 | .529 | .694 |
| R6 | .387 | .089 | .086 | .090 | -.074 | -.071 | -.061 | .409 | .526 | .536 | .538 | .500 | .908 |
| R8 | .390 | .061 | .059 | .062 | -.051 | -.048 | -.042 | .281 | .360 | .368 | .369 | .343 | .349 | .637 |
| P3 | .257 | .015 | .014 | .015 | -.025 | -.024 | -.021 | .272 | .350 | .357 | .358 | .332 | .338 | .232 | 1.300 |
| P5 | .192 | .011 | .010 | .011 | -.019 | -.018 | -.016 | .202 | .260 | .265 | .266 | .247 | .252 | .172 | .826 | 1.048 |
| P2 | .253 | .014 | .014 | .014 | -.025 | -.024 | -.021 | .267 | .344 | .351 | .352 | .327 | .332 | .228 | 1.092 | .813 | 1.238 |
| P1 | .167 | .009 | .009 | .010 | -.016 | -.016 | -.014 | .177 | .227 | .232 | .232 | .216 | .220 | .151 | .721 | .660 | .709 | .973 |
| P4 | .224 | .013 | .012 | .013 | -.022 | -.021 | -.018 | .236 | .304 | .310 | .311 | .289 | .294 | .201 | .965 | .808 | .948 | .626 | 1.292 |
| F4 | .011 | .419 | .403 | .423 | .113 | .109 | .094 | .011 | .014 | .015 | .015 | .014 | .014 | .009 | -.124 | -.092 | -.122 | -.080 | -.108 | 1.265 |
| F3 | .010 | .415 | .399 | .419 | .112 | .108 | .093 | .011 | .014 | .014 | .015 | .014 | .014 | .009 | -.123 | -.091 | -.121 | -.080 | -.107 | .813 | 1.285 |
| F2 | .012 | .491 | .472 | .496 | .133 | .127 | .110 | .013 | .017 | .017 | .017 | .016 | .016 | .011 | -.145 | -.108 | -.143 | -.094 | -.126 | .764 | .758 | 1.210 |
| F1 | .012 | .489 | .470 | .494 | .132 | .127 | .110 | .013 | .017 | .017 | .017 | .016 | .016 | .011 | -.144 | -.107 | -.142 | -.094 | -.125 | .761 | .754 | .892 | 1.336 |

##### Implied Correlations (Group number 1 - Default model)

|  | R7 | N3 | N2 | N1 | K2 | K3 | k1 | R1 | R2 | R3 | R4 | R5 | R6 | R8 | P3 | P5 | P2 | P1 | P4 | F4 | F3 | F2 | F1 |
| --- | --- | --- | --- | --- | --- | --- | --- | --- | --- | --- | --- | --- | --- | --- | --- | --- | --- | --- | --- | --- | --- | --- | --- |
| R7 | 1.000 |
| N3 | .073 | 1.000 |
| N2 | .071 | .499 | 1.000 |
| N1 | .072 | .505 | .493 | 1.000 |
| K2 | -.072 | .025 | .024 | .025 | 1.000 |
| K3 | -.068 | .023 | .023 | .023 | .845 | 1.000 |
| k1 | -.062 | .021 | .021 | .021 | .771 | .723 | 1.000 |
| R1 | .447 | .083 | .081 | .082 | -.083 | -.077 | -.071 | 1.000 |
| R2 | .526 | .098 | .096 | .097 | -.097 | -.091 | -.083 | .600 | 1.000 |
| R3 | .546 | .102 | .099 | .100 | -.101 | -.095 | -.086 | .623 | .858 | 1.000 |
| R4 | .558 | .104 | .101 | .102 | -.103 | -.097 | -.088 | .584 | .749 | .777 | 1.000 |
| R5 | .527 | .098 | .096 | .097 | -.097 | -.091 | -.083 | .601 | .708 | .734 | .750 | 1.000 |
| R6 | .468 | .087 | .085 | .086 | -.087 | -.081 | -.074 | .534 | .629 | .653 | .667 | .630 | 1.000 |
| R8 | .563 | .071 | .070 | .070 | -.071 | -.066 | -.061 | .437 | .515 | .534 | .546 | .516 | .458 | 1.000 |
| P3 | .260 | .012 | .012 | .012 | -.025 | -.023 | -.021 | .297 | .350 | .363 | .371 | .350 | .311 | .255 | 1.000 |
| P5 | .216 | .010 | .010 | .010 | -.021 | -.019 | -.018 | .246 | .290 | .301 | .307 | .290 | .258 | .211 | .708 | 1.000 |
| P2 | .262 | .012 | .012 | .012 | -.025 | -.023 | -.021 | .299 | .352 | .366 | .373 | .353 | .313 | .257 | .861 | .713 | 1.000 |
| P1 | .195 | .009 | .009 | .009 | -.019 | -.017 | -.016 | .223 | .263 | .272 | .278 | .263 | .234 | .191 | .641 | .654 | .646 | 1.000 |
| P4 | .227 | .010 | .010 | .010 | -.022 | -.020 | -.019 | .259 | .305 | .316 | .323 | .305 | .271 | .222 | .744 | .694 | .750 | .559 | 1.000 |
| F4 | .011 | .347 | .339 | .343 | .113 | .106 | .097 | .012 | .015 | .015 | .015 | .015 | .013 | .011 | -.097 | -.080 | -.097 | -.072 | -.084 | 1.000 |
| F3 | .011 | .342 | .334 | .337 | .111 | .104 | .095 | .012 | .014 | .015 | .015 | .014 | .013 | .010 | -.095 | -.079 | -.096 | -.071 | -.083 | .638 | 1.000 |
| F2 | .013 | .416 | .407 | .411 | .135 | .127 | .116 | .015 | .017 | .018 | .018 | .017 | .015 | .013 | -.116 | -.096 | -.117 | -.087 | -.101 | .618 | .608 | 1.000 |
| F1 | .012 | .394 | .385 | .389 | .128 | .120 | .110 | .014 | .016 | .017 | .017 | .016 | .015 | .012 | -.110 | -.091 | -.110 | -.082 | -.095 | .585 | .576 | .701 | 1.000 |

##### Residual Covariances (Group number 1 - Default model)

|  | R7 | N3 | N2 | N1 | K2 | K3 | k1 | R1 | R2 | R3 | R4 | R5 | R6 | R8 | P3 | P5 | P2 | P1 | P4 | F4 | F3 | F2 | F1 |
| --- | --- | --- | --- | --- | --- | --- | --- | --- | --- | --- | --- | --- | --- | --- | --- | --- | --- | --- | --- | --- | --- | --- | --- |
| R7 | .000 |
| N3 | .084 | .000 |
| N2 | .046 | -.013 | .000 |
| N1 | .009 | -.059 | .060 | .000 |
| K2 | -.047 | -.030 | -.010 | .016 | .000 |
| K3 | -.055 | -.001 | .012 | .069 | .000 | .000 |
| k1 | -.036 | -.060 | .009 | .023 | .001 | .000 | .000 |
| R1 | .007 | .072 | .075 | .075 | -.003 | -.014 | .002 | .000 |
| R2 | -.010 | .016 | -.005 | -.002 | .012 | -.005 | .000 | .015 | .000 |
| R3 | -.029 | .033 | -.002 | .011 | .026 | .015 | .023 | -.001 | .000 | .000 |
| R4 | -.006 | .028 | -.026 | -.045 | -.012 | -.009 | -.010 | .000 | -.003 | .009 | .000 |
| R5 | .032 | .027 | .042 | -.028 | .024 | .012 | .025 | -.028 | -.006 | .005 | .003 | .000 |
| R6 | -.014 | .135 | .107 | .091 | .024 | .002 | .000 | .012 | .004 | -.013 | -.007 | .020 | .000 |
| R8 | .000 | .082 | .068 | .072 | -.045 | -.036 | -.056 | .019 | -.003 | -.026 | .010 | -.004 | -.028 | .000 |
| P3 | .036 | -.032 | -.030 | .042 | .014 | -.006 | .033 | .015 | -.004 | .007 | -.062 | -.050 | -.029 | .060 | .000 |
| P5 | .116 | -.003 | -.003 | -.005 | -.015 | -.050 | .002 | .053 | .109 | .098 | .070 | .086 | .094 | .108 | -.010 | .000 |
| P2 | .062 | -.026 | .003 | .020 | -.003 | -.029 | .017 | -.002 | .008 | .009 | -.047 | -.018 | -.020 | .030 | .004 | -.002 | .000 |
| P1 | .151 | .012 | .041 | .053 | -.049 | -.050 | -.041 | .134 | .109 | .105 | .089 | .099 | .075 | .098 | -.024 | .001 | .007 | .000 |
| P4 | .020 | -.036 | -.007 | .044 | .038 | .005 | .032 | -.012 | .004 | .004 | -.069 | -.027 | .026 | .022 | .017 | .001 | -.011 | .003 | .000 |
| F4 | -.045 | .185 | .045 | -.008 | -.019 | .003 | -.074 | .063 | -.017 | -.017 | -.013 | -.026 | .091 | .027 | .060 | .044 | .087 | .047 | .103 | .000 |
| F3 | -.132 | .012 | -.036 | .027 | .060 | .100 | .036 | -.029 | -.049 | -.069 | -.104 | -.083 | -.040 | -.060 | .153 | .070 | .113 | .049 | .230 | .000 | .000 |
| F2 | -.133 | .043 | -.085 | -.016 | -.024 | .014 | -.065 | -.025 | -.093 | -.095 | -.157 | -.109 | -.005 | -.056 | .025 | -.009 | -.016 | -.022 | .081 | -.011 | .032 | .000 |
| F1 | -.145 | .075 | -.076 | .020 | .006 | .033 | -.036 | -.026 | -.122 | -.130 | -.158 | -.171 | -.048 | -.087 | -.104 | -.149 | -.095 | -.090 | -.059 | -.011 | -.037 | .007 | .000 |

##### Standardized Residual Covariances (Group number 1 - Default model)

|  | R7 | N3 | N2 | N1 | K2 | K3 | k1 | R1 | R2 | R3 | R4 | R5 | R6 | R8 | P3 | P5 | P2 | P1 | P4 | F4 | F3 | F2 | F1 |
| --- | --- | --- | --- | --- | --- | --- | --- | --- | --- | --- | --- | --- | --- | --- | --- | --- | --- | --- | --- | --- | --- | --- | --- |
| R7 | .000 |
| N3 | 2.344 | .000 |
| N2 | 1.293 | -.272 | .000 |
| N1 | .251 | -1.157 | 1.198 | .000 |
| K2 | -1.560 | -.821 | -.266 | .431 | .000 |
| K3 | -1.813 | -.027 | .314 | 1.799 | -.008 | .000 |
| k1 | -1.228 | -1.684 | .242 | .631 | .021 | -.005 | .000 |
| R1 | .245 | 2.160 | 2.293 | 2.190 | -.115 | -.484 | .070 | .000 |
| R2 | -.306 | .429 | -.133 | -.052 | .382 | -.152 | -.016 | .469 | .000 |
| R3 | -.899 | .918 | -.070 | .303 | .863 | .480 | .815 | -.016 | .000 | .000 |
| R4 | -.184 | .810 | -.757 | -1.265 | -.411 | -.300 | -.363 | .000 | -.074 | .262 | .000 |
| R5 | 1.033 | .784 | 1.222 | -.788 | .845 | .418 | .893 | -.914 | -.180 | .142 | .078 | .000 |
| R6 | -.406 | 3.408 | 2.759 | 2.264 | .730 | .055 | .003 | .355 | .100 | -.349 | -.182 | .554 | .000 |
| R8 | .000 | 2.491 | 2.082 | 2.133 | -1.632 | -1.271 | -2.100 | .711 | -.101 | -.856 | .353 | -.136 | -.877 | .000 |
| P3 | .914 | -.689 | -.656 | .879 | .368 | -.153 | .856 | .405 | -.088 | .162 | -1.563 | -1.287 | -.667 | 1.653 | .000 |
| P5 | 3.306 | -.080 | -.080 | -.113 | -.425 | -1.394 | .066 | 1.629 | 3.030 | 2.751 | 2.005 | 2.505 | 2.423 | 3.372 | -.190 | .005 |
| P2 | 1.623 | -.563 | .060 | .435 | -.086 | -.739 | .470 | -.047 | .208 | .236 | -1.222 | -.474 | -.460 | .853 | .064 | -.045 | .000 |
| P1 | 4.511 | .298 | 1.019 | 1.261 | -1.439 | -1.439 | -1.250 | 4.295 | 3.175 | 3.085 | 2.654 | 3.039 | 2.013 | 3.189 | -.467 | .014 | .138 | .000 |
| P4 | .511 | -.771 | -.145 | .907 | .969 | .128 | .850 | -.329 | .092 | .113 | -1.771 | -.702 | .600 | .626 | .271 | .014 | -.182 | .065 | .000 |
| F4 | -1.185 | 3.757 | .924 | -.151 | -.499 | .066 | -1.964 | 1.813 | -.455 | -.446 | -.355 | -.721 | 2.201 | .790 | 1.217 | .988 | 1.800 | 1.086 | 2.084 | .000 |
| F3 | -3.472 | .238 | -.734 | .534 | 1.529 | 2.498 | .937 | -.823 | -1.279 | -1.826 | -2.809 | -2.293 | -.951 | -1.734 | 3.056 | 1.565 | 2.308 | 1.141 | 4.617 | .000 | .000 |
| F2 | -3.614 | .881 | -1.756 | -.316 | -.636 | .348 | -1.756 | -.720 | -2.515 | -2.594 | -4.367 | -3.074 | -.122 | -1.643 | .512 | -.201 | -.333 | -.530 | 1.672 | -.202 | .567 | .000 |
| F1 | -3.747 | 1.467 | -1.507 | .383 | .153 | .815 | -.933 | -.724 | -3.121 | -3.378 | -4.192 | -4.619 | -1.120 | -2.448 | -2.034 | -3.265 | -1.900 | -2.052 | -1.163 | -.193 | -.635 | .113 | .000 |

##### Factor Score Weights (Group number 1 - Default model)

|  | R7 | N3 | N2 | N1 | K2 | K3 | k1 | R1 | R2 | R3 | R4 | R5 | R6 | R8 | P3 | P5 | P2 | P1 | P4 | F4 | F3 | F2 | F1 |
| --- | --- | --- | --- | --- | --- | --- | --- | --- | --- | --- | --- | --- | --- | --- | --- | --- | --- | --- | --- | --- | --- | --- | --- |
| LPU | .000 | -.001 | -.001 | -.001 | .475 | .207 | .122 | -.001 | -.001 | -.001 | -.002 | -.002 | -.001 | .000 | .001 | .000 | .001 | .000 | .000 | .001 | .001 | .004 | .003 |
| NPP | .002 | .224 | .212 | .211 | -.009 | -.004 | -.002 | .005 | .004 | .007 | .013 | .008 | .004 | .002 | .005 | .001 | .006 | .001 | .002 | .028 | .025 | .079 | .055 |
| PPP | .001 | .001 | .001 | .001 | .002 | .001 | .000 | .004 | .003 | .005 | .009 | .005 | .003 | .001 | .225 | .046 | .255 | .044 | .072 | -.001 | -.001 | -.004 | -.003 |
| NMB | .000 | .043 | .041 | .041 | .019 | .008 | .005 | .001 | .001 | .001 | .002 | .001 | .001 | .000 | -.010 | -.002 | -.012 | -.002 | -.003 | .123 | .113 | .351 | .245 |
| PMB | .045 | .003 | .003 | .003 | -.005 | -.002 | -.001 | .119 | .094 | .157 | .277 | .169 | .087 | .046 | .009 | .002 | .011 | .002 | .003 | .000 | .000 | .001 | .001 |

##### Total Effects (Group number 1 - Default model)

|  | LPU | NPP | PPP | NMB | PMB |
| --- | --- | --- | --- | --- | --- |
| NPP | .041 | .000 | .000 | .000 | .000 |
| PPP | -.028 | .000 | .000 | .000 | .000 |
| NMB | .222 | .821 | -.212 | .000 | .000 |
| PMB | -.131 | .155 | .490 | .000 | .000 |
| R7 | -.094 | .112 | .352 | .000 | .719 |
| N3 | .040 | .990 | .000 | .000 | .000 |
| N2 | .039 | .952 | .000 | .000 | .000 |
| N1 | .041 | 1.000 | .000 | .000 | .000 |
| K2 | 1.206 | .000 | .000 | .000 | .000 |
| K3 | 1.156 | .000 | .000 | .000 | .000 |
| k1 | 1.000 | .000 | .000 | .000 | .000 |
| R1 | -.099 | .118 | .372 | .000 | .760 |
| R2 | -.128 | .151 | .478 | .000 | .977 |
| R3 | -.130 | .155 | .488 | .000 | .996 |
| R4 | -.131 | .155 | .490 | .000 | 1.000 |
| R5 | -.122 | .144 | .455 | .000 | .929 |
| R6 | -.124 | .147 | .462 | .000 | .945 |
| R8 | -.085 | .100 | .317 | .000 | .648 |
| P3 | -.042 | .000 | 1.540 | .000 | .000 |
| P5 | -.032 | .000 | 1.146 | .000 | .000 |
| P2 | -.042 | .000 | 1.514 | .000 | .000 |
| P1 | -.028 | .000 | 1.000 | .000 | .000 |
| P4 | -.037 | .000 | 1.337 | .000 | .000 |
| F4 | .190 | .704 | -.181 | .857 | .000 |
| F3 | .188 | .698 | -.180 | .850 | .000 |
| F2 | .223 | .825 | -.213 | 1.005 | .000 |
| F1 | .222 | .821 | -.212 | 1.000 | .000 |

##### Standardized Total Effects (Group number 1 - Default model)

|  | LPU | NPP | PPP | NMB | PMB |
| --- | --- | --- | --- | --- | --- |
| NPP | .037 | .000 | .000 | .000 | .000 |
| PPP | -.028 | .000 | .000 | .000 | .000 |
| NMB | .165 | .674 | -.154 | .000 | .000 |
| PMB | -.122 | .159 | .444 | .000 | .000 |
| R7 | -.076 | .100 | .278 | .000 | .626 |
| N3 | .026 | .715 | .000 | .000 | .000 |
| N2 | .026 | .698 | .000 | .000 | .000 |
| N1 | .026 | .706 | .000 | .000 | .000 |
| K2 | .949 | .000 | .000 | .000 | .000 |
| K3 | .890 | .000 | .000 | .000 | .000 |
| k1 | .813 | .000 | .000 | .000 | .000 |
| R1 | -.087 | .114 | .317 | .000 | .714 |
| R2 | -.102 | .134 | .373 | .000 | .841 |
| R3 | -.106 | .139 | .387 | .000 | .872 |
| R4 | -.109 | .142 | .395 | .000 | .891 |
| R5 | -.103 | .134 | .373 | .000 | .842 |
| R6 | -.091 | .119 | .332 | .000 | .748 |
| R8 | -.075 | .097 | .272 | .000 | .613 |
| P3 | -.026 | .000 | .924 | .000 | .000 |
| P5 | -.022 | .000 | .766 | .000 | .000 |
| P2 | -.026 | .000 | .931 | .000 | .000 |
| P1 | -.020 | .000 | .694 | .000 | .000 |
| P4 | -.023 | .000 | .805 | .000 | .000 |
| F4 | .119 | .484 | -.110 | .718 | .000 |
| F3 | .117 | .476 | -.109 | .706 | .000 |
| F2 | .142 | .580 | -.132 | .861 | .000 |
| F1 | .135 | .550 | -.125 | .815 | .000 |

##### Direct Effects (Group number 1 - Default model)

|  | LPU | NPP | PPP | NMB | PMB |
| --- | --- | --- | --- | --- | --- |
| NPP | .041 | .000 | .000 | .000 | .000 |
| PPP | -.028 | .000 | .000 | .000 | .000 |
| NMB | .182 | .821 | -.212 | .000 | .000 |
| PMB | -.124 | .155 | .490 | .000 | .000 |
| R7 | .000 | .000 | .000 | .000 | .719 |
| N3 | .000 | .990 | .000 | .000 | .000 |
| N2 | .000 | .952 | .000 | .000 | .000 |
| N1 | .000 | 1.000 | .000 | .000 | .000 |
| K2 | 1.206 | .000 | .000 | .000 | .000 |
| K3 | 1.156 | .000 | .000 | .000 | .000 |
| k1 | 1.000 | .000 | .000 | .000 | .000 |
| R1 | .000 | .000 | .000 | .000 | .760 |
| R2 | .000 | .000 | .000 | .000 | .977 |
| R3 | .000 | .000 | .000 | .000 | .996 |
| R4 | .000 | .000 | .000 | .000 | 1.000 |
| R5 | .000 | .000 | .000 | .000 | .929 |
| R6 | .000 | .000 | .000 | .000 | .945 |
| R8 | .000 | .000 | .000 | .000 | .648 |
| P3 | .000 | .000 | 1.540 | .000 | .000 |
| P5 | .000 | .000 | 1.146 | .000 | .000 |
| P2 | .000 | .000 | 1.514 | .000 | .000 |
| P1 | .000 | .000 | 1.000 | .000 | .000 |
| P4 | .000 | .000 | 1.337 | .000 | .000 |
| F4 | .000 | .000 | .000 | .857 | .000 |
| F3 | .000 | .000 | .000 | .850 | .000 |
| F2 | .000 | .000 | .000 | 1.005 | .000 |
| F1 | .000 | .000 | .000 | 1.000 | .000 |

##### Standardized Direct Effects (Group number 1 - Default model)

|  | LPU | NPP | PPP | NMB | PMB |
| --- | --- | --- | --- | --- | --- |
| NPP | .037 | .000 | .000 | .000 | .000 |
| PPP | -.028 | .000 | .000 | .000 | .000 |
| NMB | .136 | .674 | -.154 | .000 | .000 |
| PMB | -.115 | .159 | .444 | .000 | .000 |
| R7 | .000 | .000 | .000 | .000 | .626 |
| N3 | .000 | .715 | .000 | .000 | .000 |
| N2 | .000 | .698 | .000 | .000 | .000 |
| N1 | .000 | .706 | .000 | .000 | .000 |
| K2 | .949 | .000 | .000 | .000 | .000 |
| K3 | .890 | .000 | .000 | .000 | .000 |
| k1 | .813 | .000 | .000 | .000 | .000 |
| R1 | .000 | .000 | .000 | .000 | .714 |
| R2 | .000 | .000 | .000 | .000 | .841 |
| R3 | .000 | .000 | .000 | .000 | .872 |
| R4 | .000 | .000 | .000 | .000 | .891 |
| R5 | .000 | .000 | .000 | .000 | .842 |
| R6 | .000 | .000 | .000 | .000 | .748 |
| R8 | .000 | .000 | .000 | .000 | .613 |
| P3 | .000 | .000 | .924 | .000 | .000 |
| P5 | .000 | .000 | .766 | .000 | .000 |
| P2 | .000 | .000 | .931 | .000 | .000 |
| P1 | .000 | .000 | .694 | .000 | .000 |
| P4 | .000 | .000 | .805 | .000 | .000 |
| F4 | .000 | .000 | .000 | .718 | .000 |
| F3 | .000 | .000 | .000 | .706 | .000 |
| F2 | .000 | .000 | .000 | .861 | .000 |
| F1 | .000 | .000 | .000 | .815 | .000 |

##### Indirect Effects (Group number 1 - Default model)

|  | LPU | NPP | PPP | NMB | PMB |
| --- | --- | --- | --- | --- | --- |
| NPP | .000 | .000 | .000 | .000 | .000 |
| PPP | .000 | .000 | .000 | .000 | .000 |
| NMB | .039 | .000 | .000 | .000 | .000 |
| PMB | -.007 | .000 | .000 | .000 | .000 |
| R7 | -.094 | .112 | .352 | .000 | .000 |
| N3 | .040 | .000 | .000 | .000 | .000 |
| N2 | .039 | .000 | .000 | .000 | .000 |
| N1 | .041 | .000 | .000 | .000 | .000 |
| K2 | .000 | .000 | .000 | .000 | .000 |
| K3 | .000 | .000 | .000 | .000 | .000 |
| k1 | .000 | .000 | .000 | .000 | .000 |
| R1 | -.099 | .118 | .372 | .000 | .000 |
| R2 | -.128 | .151 | .478 | .000 | .000 |
| R3 | -.130 | .155 | .488 | .000 | .000 |
| R4 | -.131 | .155 | .490 | .000 | .000 |
| R5 | -.122 | .144 | .455 | .000 | .000 |
| R6 | -.124 | .147 | .462 | .000 | .000 |
| R8 | -.085 | .100 | .317 | .000 | .000 |
| P3 | -.042 | .000 | .000 | .000 | .000 |
| P5 | -.032 | .000 | .000 | .000 | .000 |
| P2 | -.042 | .000 | .000 | .000 | .000 |
| P1 | -.028 | .000 | .000 | .000 | .000 |
| P4 | -.037 | .000 | .000 | .000 | .000 |
| F4 | .190 | .704 | -.181 | .000 | .000 |
| F3 | .188 | .698 | -.180 | .000 | .000 |
| F2 | .223 | .825 | -.213 | .000 | .000 |
| F1 | .222 | .821 | -.212 | .000 | .000 |

##### Standardized Indirect Effects (Group number 1 - Default model)

|  | LPU | NPP | PPP | NMB | PMB |
| --- | --- | --- | --- | --- | --- |
| NPP | .000 | .000 | .000 | .000 | .000 |
| PPP | .000 | .000 | .000 | .000 | .000 |
| NMB | .029 | .000 | .000 | .000 | .000 |
| PMB | -.007 | .000 | .000 | .000 | .000 |
| R7 | -.076 | .100 | .278 | .000 | .000 |
| N3 | .026 | .000 | .000 | .000 | .000 |
| N2 | .026 | .000 | .000 | .000 | .000 |
| N1 | .026 | .000 | .000 | .000 | .000 |
| K2 | .000 | .000 | .000 | .000 | .000 |
| K3 | .000 | .000 | .000 | .000 | .000 |
| k1 | .000 | .000 | .000 | .000 | .000 |
| R1 | -.087 | .114 | .317 | .000 | .000 |
| R2 | -.102 | .134 | .373 | .000 | .000 |
| R3 | -.106 | .139 | .387 | .000 | .000 |
| R4 | -.109 | .142 | .395 | .000 | .000 |
| R5 | -.103 | .134 | .373 | .000 | .000 |
| R6 | -.091 | .119 | .332 | .000 | .000 |
| R8 | -.075 | .097 | .272 | .000 | .000 |
| P3 | -.026 | .000 | .000 | .000 | .000 |
| P5 | -.022 | .000 | .000 | .000 | .000 |
| P2 | -.026 | .000 | .000 | .000 | .000 |
| P1 | -.020 | .000 | .000 | .000 | .000 |
| P4 | -.023 | .000 | .000 | .000 | .000 |
| F4 | .119 | .484 | -.110 | .000 | .000 |
| F3 | .117 | .476 | -.109 | .000 | .000 |
| F2 | .142 | .580 | -.132 | .000 | .000 |
| F1 | .135 | .550 | -.125 | .000 | .000 |

##### Modification Indices (Group number 1 - Default model)

##### Covariances: (Group number 1 - Default model)

|  |  |  | M.I. | Par Change |
| --- | --- | --- | --- | --- |
| e1 | <--> | e3 | 28.664 | -.119 |
| e12 | <--> | e3 | 8.508 | -.061 |
| e21 | <--> | e3 | 15.833 | .105 |
| e21 | <--> | e1 | 10.436 | .073 |
| e21 | <--> | e12 | 5.379 | .050 |
| e20 | <--> | e3 | 10.064 | -.084 |
| e19 | <--> | e21 | 10.363 | -.089 |
| e19 | <--> | e20 | 10.325 | .088 |
| e26 | <--> | e4 | 5.345 | .037 |
| e28 | <--> | e3 | 5.933 | -.042 |
| e6 | <--> | e4 | 7.574 | .055 |
| e8 | <--> | e12 | 4.066 | -.020 |
| e9 | <--> | e4 | 10.482 | -.049 |
| e9 | <--> | e5 | 6.963 | -.033 |
| e9 | <--> | e3 | 4.089 | -.029 |
| e9 | <--> | e8 | 4.820 | .015 |
| e10 | <--> | e3 | 5.761 | -.037 |
| e10 | <--> | e12 | 10.920 | .041 |
| e10 | <--> | e20 | 9.252 | .049 |
| e10 | <--> | e6 | 10.235 | -.035 |
| e11 | <--> | e4 | 19.031 | .097 |
| e11 | <--> | e10 | 4.237 | .025 |
| e13 | <--> | e4 | 5.624 | .049 |
| e13 | <--> | e8 | 4.642 | -.020 |
| e16 | <--> | e1 | 5.360 | -.033 |
| e16 | <--> | e20 | 5.161 | -.040 |
| e16 | <--> | e10 | 9.436 | -.031 |
| e16 | <--> | e13 | 10.108 | .040 |
| e18 | <--> | e1 | 18.597 | .073 |
| e18 | <--> | e6 | 5.500 | -.034 |
| e15 | <--> | e12 | 4.261 | .027 |
| e15 | <--> | e13 | 4.242 | -.025 |
| e14 | <--> | e1 | 17.876 | .079 |
| e14 | <--> | e12 | 4.946 | .039 |
| e14 | <--> | e6 | 15.393 | .062 |
| e14 | <--> | e16 | 4.713 | -.031 |
| e17 | <--> | e3 | 11.185 | .073 |
| e17 | <--> | e1 | 4.426 | -.039 |
| e17 | <--> | e9 | 7.583 | -.033 |
| e17 | <--> | e11 | 8.232 | .050 |
| e17 | <--> | e16 | 4.171 | .028 |
| e22 | <--> | e4 | 6.877 | .069 |
| e22 | <--> | e1 | 6.108 | .052 |
| e22 | <--> | e21 | 27.951 | .136 |
| e22 | <--> | e19 | 11.386 | -.089 |
| e22 | <--> | e28 | 4.073 | -.033 |
| e22 | <--> | e16 | 6.018 | -.040 |
| e22 | <--> | e15 | 6.224 | .039 |
| e23 | <--> | e2 | 9.234 | .068 |
| e23 | <--> | e5 | 14.214 | .082 |
| e23 | <--> | e1 | 9.259 | -.065 |
| e23 | <--> | e21 | 9.224 | -.080 |
| e23 | <--> | e19 | 4.651 | .058 |
| e23 | <--> | e11 | 5.518 | -.048 |
| e23 | <--> | e16 | 5.706 | .040 |
| e23 | <--> | e17 | 17.258 | .088 |
| e24 | <--> | e1 | 7.177 | -.050 |
| e24 | <--> | e20 | 4.814 | -.050 |
| e24 | <--> | e9 | 13.780 | -.045 |
| e24 | <--> | e16 | 4.668 | .031 |
| e24 | <--> | e15 | 6.752 | -.036 |
| e24 | <--> | e23 | 6.977 | .056 |
| e25 | <--> | e5 | 19.731 | -.093 |
| e25 | <--> | e10 | 10.089 | -.046 |
| e25 | <--> | e18 | 8.431 | -.054 |
| e25 | <--> | e23 | 4.012 | -.047 |

##### Variances: (Group number 1 - Default model)

|  |  |  | M.I. | Par Change |
| --- | --- | --- | --- | --- |

##### Regression Weights: (Group number 1 - Default model)

|  |  |  | M.I. | Par Change |
| --- | --- | --- | --- | --- |
| NMB | <--- | PMB | 20.878 | -.199 |
| PMB | <--- | NMB | 10.579 | -.099 |
| R7 | <--- | NMB | 5.798 | -.069 |
| R7 | <--- | P2 | 4.622 | .049 |
| R7 | <--- | P1 | 7.771 | .071 |
| R7 | <--- | F4 | 4.049 | -.045 |
| R7 | <--- | F3 | 7.407 | -.061 |
| R7 | <--- | F2 | 5.808 | -.055 |
| N3 | <--- | NMB | 5.966 | .091 |
| N3 | <--- | PMB | 6.230 | .112 |
| N3 | <--- | R7 | 11.625 | .129 |
| N3 | <--- | N1 | 4.412 | -.063 |
| N3 | <--- | k1 | 4.282 | -.078 |
| N3 | <--- | R4 | 8.054 | .110 |
| N3 | <--- | R6 | 7.166 | .092 |
| N3 | <--- | R8 | 4.559 | .088 |
| N3 | <--- | F4 | 22.543 | .138 |
| N3 | <--- | F2 | 4.283 | .062 |
| N3 | <--- | F1 | 7.036 | .075 |
| N2 | <--- | N1 | 4.361 | .062 |
| N2 | <--- | R5 | 6.049 | .096 |
| N2 | <--- | F2 | 6.110 | -.073 |
| N2 | <--- | F1 | 5.929 | -.069 |
| N1 | <--- | N3 | 4.244 | -.065 |
| N1 | <--- | N2 | 4.509 | .068 |
| K3 | <--- | NPP | 5.197 | .061 |
| K3 | <--- | NMB | 5.301 | .048 |
| K3 | <--- | N1 | 6.987 | .044 |
| K3 | <--- | P5 | 4.119 | -.036 |
| K3 | <--- | F3 | 5.459 | .038 |
| K3 | <--- | F2 | 4.238 | .034 |
| k1 | <--- | NMB | 5.766 | -.056 |
| k1 | <--- | F4 | 7.515 | -.051 |
| k1 | <--- | F2 | 6.033 | -.046 |
| k1 | <--- | F1 | 4.053 | -.036 |
| R1 | <--- | NPP | 7.490 | .091 |
| R1 | <--- | NMB | 5.394 | .060 |
| R1 | <--- | N2 | 4.877 | .048 |
| R1 | <--- | N1 | 7.044 | .055 |
| R1 | <--- | P1 | 7.211 | .062 |
| R1 | <--- | F4 | 5.898 | .049 |
| R1 | <--- | F1 | 6.940 | .052 |
| R3 | <--- | R7 | 4.556 | -.037 |
| R3 | <--- | R8 | 5.038 | -.042 |
| R4 | <--- | NPP | 11.162 | -.085 |
| R4 | <--- | PPP | 7.067 | -.070 |
| R4 | <--- | NMB | 9.738 | -.062 |
| R4 | <--- | N2 | 10.563 | -.054 |
| R4 | <--- | N1 | 10.309 | -.051 |
| R4 | <--- | P3 | 7.089 | -.041 |
| R4 | <--- | P2 | 6.616 | -.040 |
| R4 | <--- | P4 | 12.315 | -.054 |
| R4 | <--- | F3 | 4.775 | -.034 |
| R4 | <--- | F2 | 16.282 | -.064 |
| R5 | <--- | R7 | 5.537 | .051 |
| R5 | <--- | R1 | 4.625 | -.050 |
| R5 | <--- | F1 | 9.038 | -.049 |
| R6 | <--- | NPP | 19.221 | .162 |
| R6 | <--- | NMB | 12.537 | .102 |
| R6 | <--- | N3 | 13.159 | .086 |
| R6 | <--- | N2 | 12.286 | .084 |
| R6 | <--- | N1 | 11.408 | .078 |
| R6 | <--- | F4 | 10.729 | .074 |
| R6 | <--- | F2 | 11.424 | .078 |
| R6 | <--- | F1 | 6.827 | .057 |
| R8 | <--- | NPP | 5.439 | .080 |
| R8 | <--- | N1 | 7.094 | .057 |
| R8 | <--- | k1 | 5.558 | -.064 |
| R8 | <--- | P3 | 5.185 | .047 |
| P3 | <--- | PMB | 4.713 | -.062 |
| P3 | <--- | R4 | 5.534 | -.058 |
| P3 | <--- | R5 | 10.829 | -.082 |
| P3 | <--- | R6 | 4.256 | -.045 |
| P5 | <--- | PMB | 13.446 | .122 |
| P5 | <--- | R7 | 6.056 | .069 |
| P5 | <--- | R2 | 12.801 | .099 |
| P5 | <--- | R3 | 9.379 | .086 |
| P5 | <--- | R4 | 14.719 | .110 |
| P5 | <--- | R5 | 13.446 | .107 |
| P5 | <--- | R6 | 10.204 | .081 |
| P5 | <--- | R8 | 9.939 | .096 |
| P5 | <--- | F1 | 7.280 | -.057 |
| P1 | <--- | PMB | 16.260 | .148 |
| P1 | <--- | R7 | 15.984 | .123 |
| P1 | <--- | k1 | 4.842 | -.068 |
| P1 | <--- | R1 | 27.886 | .176 |
| P1 | <--- | R2 | 9.003 | .092 |
| P1 | <--- | R3 | 8.601 | .091 |
| P1 | <--- | R4 | 13.461 | .116 |
| P1 | <--- | R5 | 13.233 | .117 |
| P1 | <--- | R6 | 4.048 | .057 |
| P1 | <--- | R8 | 4.424 | .071 |
| P4 | <--- | NMB | 7.418 | .082 |
| P4 | <--- | R4 | 6.874 | -.082 |
| P4 | <--- | F3 | 19.824 | .104 |
| P4 | <--- | F2 | 8.605 | .071 |
| F4 | <--- | NPP | 6.656 | .114 |
| F4 | <--- | PMB | 10.943 | .137 |
| F4 | <--- | R7 | 4.802 | .076 |
| F4 | <--- | N3 | 24.026 | .138 |
| F4 | <--- | N2 | 7.813 | .080 |
| F4 | <--- | k1 | 5.885 | -.085 |
| F4 | <--- | R1 | 10.240 | .121 |
| F4 | <--- | R4 | 12.241 | .125 |
| F4 | <--- | R5 | 5.156 | .083 |
| F4 | <--- | R6 | 13.050 | .115 |
| F4 | <--- | R8 | 9.066 | .114 |
| F3 | <--- | LPU | 9.234 | .138 |
| F3 | <--- | PPP | 13.445 | .171 |
| F3 | <--- | R7 | 5.395 | -.083 |
| F3 | <--- | N3 | 5.501 | -.068 |
| F3 | <--- | K2 | 6.927 | .091 |
| F3 | <--- | K3 | 9.741 | .106 |
| F3 | <--- | k1 | 10.195 | .114 |
| F3 | <--- | R6 | 6.364 | -.082 |
| F3 | <--- | P3 | 16.195 | .109 |
| F3 | <--- | P5 | 6.449 | .077 |
| F3 | <--- | P2 | 8.361 | .080 |
| F3 | <--- | P4 | 26.775 | .141 |
| F2 | <--- | PMB | 4.859 | -.081 |
| F2 | <--- | R7 | 4.704 | -.067 |
| F2 | <--- | N2 | 4.736 | -.055 |
| F2 | <--- | R4 | 10.752 | -.104 |
| F1 | <--- | PPP | 19.838 | -.199 |
| F1 | <--- | PMB | 11.759 | -.139 |
| F1 | <--- | R7 | 5.360 | -.079 |
| F1 | <--- | R2 | 6.893 | -.089 |
| F1 | <--- | R3 | 8.618 | -.101 |
| F1 | <--- | R4 | 6.954 | -.092 |
| F1 | <--- | R5 | 19.067 | -.156 |
| F1 | <--- | R6 | 6.724 | -.081 |
| F1 | <--- | R8 | 7.541 | -.102 |
| F1 | <--- | P3 | 18.909 | -.113 |
| F1 | <--- | P5 | 28.587 | -.155 |
| F1 | <--- | P2 | 12.705 | -.095 |
| F1 | <--- | P1 | 11.652 | -.103 |
| F1 | <--- | P4 | 19.442 | -.115 |

##### Minimization History (Default model)

| Iteration |  | Negative eigenvalues | Condition # | Smallest eigenvalue | Diameter | F | NTries | Ratio |
| --- | --- | --- | --- | --- | --- | --- | --- | --- |
| 0 | e | 14 |  | -1.378 | 9999.000 | 10449.511 | 0 | 9999.000 |
| 1 | e | 16 |  | -.944 | 2.157 | 6423.552 | 19 | .499 |
| 2 | e | 13 |  | -.249 | .601 | 5088.257 | 6 | 1.005 |
| 3 | e | 11 |  | -.252 | .201 | 4673.888 | 6 | .902 |
| 4 | e | 4 |  | -.284 | 1.345 | 2423.140 | 8 | .927 |
| 5 | e | 1 |  | -.214 | .527 | 1601.655 | 5 | .937 |
| 6 | e | 0 | 370.268 |  | .475 | 1111.159 | 5 | .883 |
| 7 | e | 0 | 160.417 |  | 1.292 | 912.656 | 2 | .000 |
| 8 | e | 0 | 216.690 |  | .609 | 680.296 | 1 | 1.024 |
| 9 | e | 0 | 280.145 |  | .294 | 639.600 | 1 | 1.110 |
| 10 | e | 0 | 335.024 |  | .079 | 637.649 | 1 | 1.046 |
| 11 | e | 0 | 329.272 |  | .008 | 637.638 | 1 | 1.005 |
| 12 | e | 0 | 329.371 |  | .000 | 637.638 | 1 | 1.000 |

##### Pairwise Parameter Comparisons (Default model)

##### Variance-covariance Matrix of Estimates (Default model)

|  | par\_1 | par\_2 | par\_3 | par\_4 | par\_5 | par\_6 | par\_7 | par\_8 | par\_9 | par\_10 | par\_11 | par\_12 | par\_13 | par\_14 | par\_15 | par\_16 | par\_17 | par\_18 | par\_19 | par\_20 | par\_21 | par\_22 | par\_23 | par\_24 | par\_25 | par\_26 | par\_27 | par\_28 | par\_29 | par\_30 | par\_31 | par\_32 | par\_33 | par\_34 | par\_35 | par\_36 | par\_37 | par\_38 | par\_39 | par\_40 | par\_41 | par\_42 | par\_43 | par\_44 | par\_45 | par\_46 | par\_47 | par\_48 | par\_49 | par\_50 | par\_51 | par\_52 | par\_53 | par\_54 | par\_55 | par\_56 | par\_57 | par\_58 | par\_59 | par\_60 | par\_61 |
| --- | --- | --- | --- | --- | --- | --- | --- | --- | --- | --- | --- | --- | --- | --- | --- | --- | --- | --- | --- | --- | --- | --- | --- | --- | --- | --- | --- | --- | --- | --- | --- | --- | --- | --- | --- | --- | --- | --- | --- | --- | --- | --- | --- | --- | --- | --- | --- | --- | --- | --- | --- | --- | --- | --- | --- | --- | --- | --- | --- | --- | --- |
| par\_1 | .001 |
| par\_2 | .000 | .005 |
| par\_3 | .000 | .003 | .005 |
| par\_4 | .000 | .002 | .002 | .003 |
| par\_5 | .000 | .000 | .000 | .000 | .000 |
| par\_6 | .000 | .000 | .000 | .000 | .000 | .000 |
| par\_7 | .000 | .000 | .000 | .000 | .000 | .000 | .001 |
| par\_8 | .000 | .000 | .000 | .000 | .000 | .000 | .000 | .002 |
| par\_9 | .000 | .000 | .000 | .000 | .000 | .000 | .000 | .000 | .001 |
| par\_10 | .000 | .000 | .000 | .000 | .000 | .000 | .000 | .000 | .000 | .001 |
| par\_11 | .000 | .000 | .000 | .000 | .000 | .000 | .000 | .000 | .000 | .001 | .001 |
| par\_12 | .000 | .000 | .000 | .000 | .000 | .000 | .000 | .000 | .000 | .000 | .000 | .001 |
| par\_13 | .000 | .000 | .000 | .000 | .000 | .000 | .000 | .000 | .000 | .000 | .000 | .000 | .000 |
| par\_14 | .000 | .000 | .000 | .000 | .000 | .000 | .000 | .000 | .000 | .000 | .000 | .000 | .000 | .000 |
| par\_15 | .000 | .000 | .000 | .000 | .000 | .000 | .000 | .000 | .000 | .000 | .000 | .000 | .000 | .000 | .002 |
| par\_16 | .000 | .000 | .000 | .000 | .000 | .000 | .000 | .000 | .000 | .000 | .000 | .000 | .000 | .000 | .000 | .004 |
| par\_17 | .000 | .000 | .000 | .000 | .000 | .000 | .000 | .000 | .000 | .000 | .000 | .000 | .000 | .000 | .000 | .000 | .002 |
| par\_18 | .000 | .000 | .000 | .000 | .000 | .000 | .000 | .000 | .000 | .000 | .000 | .000 | .000 | .000 | .000 | .000 | .000 | .002 |
| par\_19 | .000 | .000 | .000 | .000 | .000 | .000 | .000 | .000 | .000 | .000 | .000 | .000 | .000 | .000 | .000 | .000 | .000 | .000 | .002 |
| par\_20 | .000 | .000 | .000 | .000 | .000 | .000 | .000 | .000 | .000 | .000 | .000 | .000 | .000 | .000 | .000 | .000 | .000 | -.001 | .000 | .003 |
| par\_21 | .000 | .001 | .001 | .001 | .000 | .000 | .000 | .000 | .000 | .000 | .000 | .000 | .000 | .000 | .000 | .000 | .000 | .000 | .000 | .000 | .002 |
| par\_22 | .000 | .000 | -.001 | .000 | .000 | .000 | .000 | .000 | .000 | .000 | .000 | .000 | .000 | .000 | .000 | .000 | .000 | .000 | .000 | .000 | .000 | .003 |
| par\_23 | .000 | .000 | .000 | .000 | .000 | .000 | .000 | .000 | .000 | .000 | .000 | .000 | .000 | .000 | .000 | .000 | .000 | .000 | .000 | .000 | .000 | .000 | .002 |
| par\_24 | .000 | .000 | .000 | .000 | .000 | .000 | .000 | .000 | .000 | .000 | .000 | .000 | .000 | .000 | .000 | .002 | .000 | .000 | .000 | .000 | .000 | .000 | .001 | .005 |
| par\_25 | .000 | .000 | .000 | .000 | .000 | .000 | .000 | .000 | .000 | .000 | .000 | .000 | .000 | .000 | .000 | .000 | .000 | .000 | .000 | .000 | .000 | .000 | .000 | .000 | .001 |
| par\_26 | .000 | .000 | .000 | .000 | .000 | .000 | .001 | .000 | .000 | .000 | .000 | .000 | .000 | .000 | .000 | .000 | .000 | .000 | .000 | .000 | .000 | .000 | .000 | .000 | .000 | .002 |
| par\_27 | .000 | .000 | .000 | .000 | .000 | .000 | .000 | .000 | .000 | .000 | .000 | .000 | .000 | .000 | .000 | .000 | .000 | .000 | .000 | .000 | .000 | .000 | .000 | .000 | .000 | .000 | .000 |
| par\_28 | .000 | .000 | .000 | .000 | .000 | .000 | .000 | .000 | .000 | .000 | .000 | .000 | .000 | .000 | .000 | .000 | .000 | .000 | .000 | .000 | .000 | .000 | .000 | .000 | .000 | .000 | .000 | .002 |
| par\_29 | .000 | .000 | .000 | .000 | .000 | .000 | .000 | .000 | .000 | .000 | .000 | .000 | .000 | .000 | .000 | .000 | .000 | .000 | .000 | .000 | .000 | .000 | .000 | .000 | .000 | .000 | .000 | .001 | .002 |
| par\_30 | .000 | .000 | .000 | .000 | .000 | .000 | .000 | .000 | .000 | .000 | .000 | .000 | .000 | .000 | .000 | .000 | .000 | .000 | .000 | .000 | .000 | .000 | .000 | .000 | .000 | .000 | .000 | .001 | .001 | .002 |
| par\_31 | .000 | .000 | .000 | .000 | .000 | .000 | .000 | .000 | .000 | .000 | .000 | .000 | .000 | .000 | .001 | .000 | .000 | .000 | .000 | .000 | .000 | .000 | .000 | .000 | .000 | .000 | .000 | .000 | .000 | .000 | .002 |
| par\_32 | .000 | .004 | .004 | .002 | .000 | .000 | .000 | .000 | .000 | .000 | .000 | .000 | .000 | .000 | .000 | .000 | .000 | .000 | .000 | .000 | .001 | -.001 | .000 | .000 | .000 | .000 | .000 | .000 | .000 | .000 | .000 | .005 |
| par\_33 | .000 | .000 | .000 | .000 | .000 | .000 | .000 | .000 | .000 | .000 | .000 | .000 | .000 | .000 | .000 | .002 | .000 | .000 | .000 | .000 | .000 | .000 | .000 | .003 | .000 | .000 | .000 | -.001 | -.001 | -.001 | .000 | .000 | .004 |
| par\_34 | .000 | .000 | .000 | .000 | .000 | .000 | .000 | .000 | .000 | .000 | .000 | .000 | .000 | .000 | -.001 | .000 | .000 | .000 | .000 | .000 | .000 | .000 | .000 | .000 | .000 | .000 | .000 | .000 | .000 | .000 | -.001 | .000 | .000 | .002 |
| par\_35 | .000 | .000 | .000 | .000 | .000 | .000 | .000 | .000 | .000 | .000 | .000 | .000 | .000 | .000 | .000 | -.002 | .000 | .000 | .000 | .000 | .000 | .000 | .000 | -.003 | .000 | .000 | .000 | .000 | .000 | .000 | .000 | .000 | -.002 | .000 | .004 |
| par\_36 | .000 | -.002 | -.002 | -.001 | .000 | .000 | .000 | .000 | .000 | .000 | .000 | .000 | .000 | .000 | .000 | .000 | .000 | .000 | .000 | .000 | -.001 | .000 | .000 | .000 | .000 | .000 | .000 | .000 | .000 | .000 | .000 | -.003 | .000 | .000 | .000 | .002 |
| par\_37 | .000 | .000 | .000 | .000 | .000 | .000 | .000 | .000 | .000 | .000 | .000 | .000 | .000 | .000 | .000 | .000 | .000 | .000 | .000 | .000 | .000 | .000 | .000 | .000 | .000 | .000 | .000 | .000 | .000 | .000 | .000 | .000 | .000 | .000 | .000 | .000 | .001 |
| par\_38 | .000 | .000 | .000 | .000 | .000 | .000 | .000 | .000 | .000 | .000 | .000 | .000 | .000 | .000 | .000 | .000 | .000 | .000 | .000 | .000 | .000 | .000 | .000 | .000 | .000 | .000 | .000 | -.001 | -.001 | -.001 | .000 | .000 | .000 | .000 | .000 | .000 | .000 | .002 |
| par\_39 | .000 | .000 | .000 | .000 | .000 | .000 | .000 | .000 | .000 | .000 | .000 | .000 | .000 | .000 | .000 | .000 | .000 | .000 | .000 | .000 | .000 | .000 | .000 | .000 | .000 | .000 | .000 | .000 | .000 | .001 | .000 | .000 | .000 | .000 | .000 | .000 | .000 | .000 | .001 |
| par\_40 | .000 | .000 | .000 | .000 | .000 | .000 | .000 | .000 | .000 | .000 | .000 | .000 | .000 | .000 | .000 | .000 | .000 | .000 | .000 | .000 | .000 | .000 | .000 | .000 | .000 | .000 | .000 | .000 | .000 | .000 | .000 | .000 | .000 | .000 | .000 | .000 | .000 | .000 | .000 | .001 |
| par\_41 | .001 | .000 | .000 | .000 | .000 | .000 | .000 | .000 | .000 | .000 | .000 | .000 | .000 | .000 | .000 | .000 | .000 | .000 | .000 | .000 | .000 | .000 | .000 | .000 | .000 | .000 | .000 | .000 | .000 | .000 | .000 | .000 | .000 | .000 | .000 | .000 | .000 | .000 | .000 | .000 | .002 |
| par\_42 | .001 | .000 | .000 | .000 | .000 | .000 | .000 | .000 | .000 | .000 | .000 | .000 | .000 | .000 | .000 | .000 | .000 | .000 | .000 | .000 | .000 | .000 | .000 | .000 | .000 | .000 | .000 | .000 | .000 | .000 | .000 | .000 | .000 | .000 | .000 | .000 | .000 | .000 | .000 | .000 | .000 | .002 |
| par\_43 | .000 | .000 | .000 | .000 | .000 | .000 | .000 | .000 | .000 | .000 | .000 | .000 | .000 | .000 | .000 | .000 | .000 | .000 | .000 | .000 | .000 | .000 | .000 | .000 | .000 | .000 | .000 | .000 | .000 | .000 | .000 | .000 | .000 | .000 | .000 | .000 | .000 | .000 | .000 | .000 | .000 | .000 | .001 |
| par\_44 | .000 | .000 | .000 | .000 | .000 | .000 | .000 | .000 | .000 | .000 | .000 | .000 | .000 | .000 | .000 | .000 | .000 | .000 | .000 | .000 | .000 | .000 | .000 | .000 | .000 | .000 | .000 | .000 | .000 | .000 | .000 | .000 | .000 | .000 | .000 | .000 | .000 | .000 | .000 | .000 | .000 | .000 | .000 | .001 |
| par\_45 | .000 | .000 | .000 | .000 | .000 | .000 | .000 | .000 | .000 | .000 | .000 | .000 | .000 | .000 | .000 | .000 | .000 | .000 | .000 | .000 | .000 | .000 | .000 | .000 | .000 | .000 | .000 | .000 | .000 | .000 | .000 | .000 | .000 | .000 | .000 | .000 | .000 | .000 | .000 | .000 | .000 | .000 | .000 | .000 | .000 |
| par\_46 | .000 | .000 | .000 | .000 | .000 | .000 | .000 | .000 | .000 | .000 | .000 | .000 | .000 | .000 | .000 | .000 | .000 | .000 | .000 | .000 | .000 | .000 | .000 | .000 | .000 | .000 | .000 | .000 | .000 | .000 | .000 | .000 | .000 | .000 | .000 | .000 | .000 | .000 | .000 | .000 | .000 | .000 | .000 | .000 | .000 | .000 |
| par\_47 | .000 | .000 | .000 | .000 | .000 | .000 | .000 | .000 | .000 | .000 | .000 | .000 | .000 | .000 | .000 | .000 | .000 | .000 | .000 | .000 | .000 | .000 | .000 | .000 | .000 | .000 | .000 | .000 | .000 | .000 | .000 | .000 | .000 | .000 | .000 | .000 | .000 | .000 | .000 | .000 | .000 | .000 | .000 | .000 | .000 | .000 | .001 |
| par\_48 | .000 | .000 | .000 | .000 | .000 | .000 | .000 | .000 | .000 | .000 | .000 | .000 | .000 | .000 | .000 | .000 | .000 | .000 | .000 | .000 | .000 | .000 | .000 | .000 | .000 | .000 | .000 | .000 | .000 | .000 | .000 | .000 | .000 | .000 | .000 | .000 | .000 | .000 | .000 | .000 | .000 | .000 | .000 | .000 | .000 | .000 | .000 | .001 |
| par\_49 | .000 | .000 | .000 | .000 | .000 | .000 | .000 | .000 | .000 | .000 | .000 | .000 | .000 | .000 | .000 | .000 | .000 | .000 | .000 | .000 | .000 | .000 | .000 | .000 | .000 | .000 | .000 | .000 | .000 | .000 | .000 | .000 | .000 | .000 | .000 | .000 | .000 | .000 | .000 | .000 | .000 | .000 | .000 | .000 | .000 | .000 | .000 | .000 | .001 |
| par\_50 | .000 | .000 | .000 | .000 | .000 | .000 | .000 | .000 | .000 | .000 | .000 | .000 | .000 | .000 | .000 | .000 | .000 | .000 | .000 | .000 | .000 | .000 | .000 | .000 | .000 | .000 | .000 | .000 | .000 | .000 | .000 | .000 | .000 | .000 | .000 | .000 | .000 | .000 | .000 | .000 | .000 | .000 | .000 | .000 | .000 | .000 | .000 | .000 | .000 | .000 |
| par\_51 | .000 | .000 | .000 | .000 | .000 | .000 | .000 | .000 | .000 | .000 | .000 | .000 | .000 | .000 | .000 | .000 | .000 | .000 | .000 | .000 | .000 | .000 | .000 | .000 | .000 | .000 | .000 | .000 | .000 | .000 | .000 | .000 | .000 | .000 | .000 | .000 | .000 | .000 | .000 | .000 | .000 | .000 | .000 | .000 | .000 | .000 | .000 | .000 | .000 | .000 | .000 |
| par\_52 | .000 | .000 | .000 | .000 | .000 | .000 | .000 | .000 | .000 | .000 | .000 | .000 | .000 | .000 | .000 | .000 | .000 | .000 | .000 | .000 | .000 | .000 | .000 | .000 | .000 | .000 | .000 | .000 | .000 | .000 | .000 | .000 | .000 | .000 | .000 | .000 | .000 | .000 | .000 | .000 | .000 | .000 | .000 | .000 | .000 | .000 | .000 | .000 | .000 | .000 | .000 | .000 |
| par\_53 | .000 | .000 | .000 | .000 | .000 | .000 | .000 | .000 | .000 | .000 | .000 | .000 | .000 | .000 | .000 | .000 | .000 | .000 | .000 | .000 | .000 | .000 | .000 | .000 | .000 | .000 | .000 | .000 | .000 | .000 | .000 | .000 | .000 | .000 | .000 | .000 | .000 | .000 | .000 | .000 | .000 | .000 | .000 | .000 | .000 | .000 | .000 | .000 | .000 | .000 | .000 | .000 | .000 |
| par\_54 | .000 | .000 | .000 | .000 | .000 | .000 | .000 | .000 | .000 | .000 | .000 | .000 | .000 | .000 | .000 | .000 | .000 | .000 | .000 | .000 | .000 | .000 | .000 | .000 | .000 | .000 | .000 | .000 | .000 | .000 | .000 | .000 | .000 | .000 | .000 | .000 | .000 | .000 | .000 | .000 | .000 | .000 | .000 | .000 | .000 | .000 | .000 | .000 | .000 | .000 | .000 | .000 | .000 | .000 |
| par\_55 | .000 | .000 | .000 | .000 | .000 | .000 | .000 | .000 | .000 | .000 | .000 | .000 | .000 | .000 | .000 | .000 | .000 | .000 | .000 | .000 | .000 | .000 | .000 | .000 | .000 | .000 | .000 | .000 | .000 | .000 | .000 | .000 | .000 | .000 | .000 | .000 | .000 | .000 | .000 | .000 | .000 | .000 | .000 | .000 | .000 | .000 | .000 | .000 | .000 | .000 | .000 | .000 | .000 | .000 | .000 |
| par\_56 | .000 | .000 | .000 | .000 | .000 | .000 | .000 | .000 | .000 | .000 | .000 | .000 | .000 | .000 | .000 | .000 | .000 | .000 | .000 | .000 | .000 | .000 | .000 | .000 | .000 | .000 | .000 | .000 | .000 | .000 | .000 | .000 | .000 | .000 | .000 | .000 | .000 | .000 | .000 | .000 | .000 | .000 | .000 | .000 | .000 | .000 | .000 | .000 | .000 | .000 | .000 | .000 | .000 | .000 | .000 | .000 |
| par\_57 | .000 | .000 | .000 | .000 | .000 | .000 | .000 | .000 | .000 | .000 | .000 | .000 | .000 | .000 | .000 | .000 | .000 | .000 | .000 | .000 | .000 | .000 | .000 | .000 | .000 | .000 | .000 | .000 | .000 | .000 | .000 | .000 | .000 | .000 | .000 | .000 | .000 | .000 | .000 | .000 | .000 | .000 | .000 | .000 | .000 | .000 | .000 | .000 | .000 | .000 | .000 | .000 | .000 | .000 | .000 | .000 | .000 |
| par\_58 | .000 | .000 | .000 | .000 | .000 | .000 | .000 | .000 | .000 | .000 | .000 | .000 | .000 | .000 | .000 | .001 | .000 | .000 | .000 | .000 | .000 | .000 | .000 | .002 | .000 | .000 | .000 | .000 | .000 | .000 | .000 | .000 | .001 | .000 | -.001 | .000 | .000 | .000 | .000 | .000 | .000 | .000 | .000 | .000 | .000 | .000 | .000 | .000 | .000 | .000 | .000 | .000 | .000 | .000 | .000 | .000 | .000 | .002 |
| par\_59 | .000 | .000 | .000 | .000 | .000 | .000 | .000 | .000 | .000 | .000 | .000 | .000 | .000 | .000 | .000 | -.001 | .000 | .000 | .000 | .000 | .000 | .000 | .000 | .000 | .000 | .000 | .000 | .000 | .000 | .000 | .000 | .000 | .000 | .000 | .000 | .000 | .000 | .000 | .000 | .000 | .000 | .000 | .000 | .000 | .000 | .000 | .000 | .000 | .000 | .000 | .000 | .000 | .000 | .000 | .000 | .000 | .000 | .000 | .002 |
| par\_60 | .000 | .000 | .000 | .000 | .000 | .000 | .000 | .000 | .000 | .000 | .000 | .000 | .000 | .000 | .000 | .000 | .000 | .000 | .000 | .000 | .000 | .000 | .000 | -.002 | .000 | .000 | .000 | .000 | .000 | .000 | .000 | .000 | -.001 | .000 | .001 | .000 | .000 | .000 | .000 | .000 | .000 | .000 | .000 | .000 | .000 | .000 | .000 | .000 | .000 | .000 | .000 | .000 | .000 | .000 | .000 | .000 | .000 | -.001 | .000 | .002 |
| par\_61 | .000 | .000 | .000 | .000 | .000 | .000 | .000 | .000 | .000 | .000 | .000 | .000 | .000 | .000 | .000 | .000 | .000 | .000 | .000 | .000 | .000 | .000 | .000 | .000 | .000 | .000 | .000 | .000 | .000 | .000 | .000 | .000 | .000 | .000 | .000 | .000 | .000 | .000 | .000 | .000 | .000 | .000 | .000 | .000 | .000 | .000 | .000 | .000 | .000 | .000 | .000 | .000 | .000 | .000 | .000 | .000 | .000 | .000 | .000 | .000 | .001 |

##### Correlations of Estimates (Default model)

|  | par\_1 | par\_2 | par\_3 | par\_4 | par\_5 | par\_6 | par\_7 | par\_8 | par\_9 | par\_10 | par\_11 | par\_12 | par\_13 | par\_14 | par\_15 | par\_16 | par\_17 | par\_18 | par\_19 | par\_20 | par\_21 | par\_22 | par\_23 | par\_24 | par\_25 | par\_26 | par\_27 | par\_28 | par\_29 | par\_30 | par\_31 | par\_32 | par\_33 | par\_34 | par\_35 | par\_36 | par\_37 | par\_38 | par\_39 | par\_40 | par\_41 | par\_42 | par\_43 | par\_44 | par\_45 | par\_46 | par\_47 | par\_48 | par\_49 | par\_50 | par\_51 | par\_52 | par\_53 | par\_54 | par\_55 | par\_56 | par\_57 | par\_58 | par\_59 | par\_60 | par\_61 |
| --- | --- | --- | --- | --- | --- | --- | --- | --- | --- | --- | --- | --- | --- | --- | --- | --- | --- | --- | --- | --- | --- | --- | --- | --- | --- | --- | --- | --- | --- | --- | --- | --- | --- | --- | --- | --- | --- | --- | --- | --- | --- | --- | --- | --- | --- | --- | --- | --- | --- | --- | --- | --- | --- | --- | --- | --- | --- | --- | --- | --- | --- |
| par\_1 | 1.000 |
| par\_2 | -.001 | 1.000 |
| par\_3 | .000 | .750 | 1.000 |
| par\_4 | .000 | .664 | .653 | 1.000 |
| par\_5 | -.001 | .065 | .080 | .009 | 1.000 |
| par\_6 | .000 | -.052 | .022 | -.079 | .080 | 1.000 |
| par\_7 | .000 | .000 | .000 | .000 | -.001 | -.001 | 1.000 |
| par\_8 | .000 | .000 | .000 | .000 | .000 | -.001 | .189 | 1.000 |
| par\_9 | .000 | .000 | .000 | .000 | -.001 | -.001 | .237 | .338 | 1.000 |
| par\_10 | .000 | .000 | .000 | .000 | .000 | -.001 | .245 | .349 | .436 | 1.000 |
| par\_11 | .000 | .000 | .000 | .000 | -.001 | -.001 | .234 | .331 | .404 | .707 | 1.000 |
| par\_12 | .000 | .000 | -.001 | -.001 | .000 | .000 | .215 | .287 | .334 | .372 | .352 | 1.000 |
| par\_13 | .000 | .000 | .000 | -.001 | .000 | .001 | .007 | -.013 | -.020 | -.177 | -.184 | -.007 | 1.000 |
| par\_14 | .000 | .001 | .001 | .001 | -.001 | -.002 | .031 | .075 | .128 | .105 | .094 | -.104 | -.091 | 1.000 |
| par\_15 | .000 | .000 | .000 | .000 | .000 | .000 | .000 | .000 | .000 | .000 | .000 | .000 | .000 | .000 | 1.000 |
| par\_16 | -.013 | .000 | .000 | .000 | .000 | .000 | .000 | .001 | .001 | .000 | .000 | .000 | .002 | .001 | .000 | 1.000 |
| par\_17 | .000 | -.005 | -.003 | -.002 | -.006 | .001 | .019 | .040 | .053 | .058 | .050 | .036 | -.022 | .021 | -.074 | .003 | 1.000 |
| par\_18 | -.010 | .002 | .001 | .001 | .002 | -.001 | .000 | .000 | .000 | .000 | .000 | .000 | .001 | .000 | .093 | .021 | .013 | 1.000 |
| par\_19 | .000 | .032 | .031 | .024 | .012 | -.002 | .000 | .000 | .000 | .000 | .000 | .000 | .000 | .000 | -.024 | .000 | -.027 | .008 | 1.000 |
| par\_20 | .002 | .000 | .000 | .000 | .000 | .000 | .000 | .000 | .000 | .000 | .000 | .000 | .000 | .000 | .029 | -.049 | -.050 | -.213 | .014 | 1.000 |
| par\_21 | .001 | .350 | .397 | .313 | .007 | -.013 | -.073 | -.108 | -.134 | -.137 | -.128 | -.119 | .000 | -.005 | -.001 | .003 | .015 | .001 | .021 | -.001 | 1.000 |
| par\_22 | -.091 | -.129 | -.155 | -.119 | -.002 | -.006 | .000 | .000 | .000 | .000 | .000 | .000 | .000 | -.001 | .002 | .022 | .001 | .014 | -.015 | -.003 | -.048 | 1.000 |
| par\_23 | -.003 | .001 | .001 | .002 | .000 | -.001 | -.015 | -.018 | -.043 | -.046 | -.043 | -.025 | .017 | -.013 | .002 | .155 | -.046 | .009 | .000 | -.007 | -.005 | .009 | 1.000 |
| par\_24 | -.017 | .000 | .000 | .000 | -.001 | .000 | .000 | .000 | .000 | -.001 | -.001 | .000 | .002 | .000 | .000 | .477 | .007 | .039 | .000 | -.078 | .007 | .029 | .178 | 1.000 |
| par\_25 | .001 | -.018 | -.020 | -.016 | -.006 | .002 | .000 | .000 | .000 | .000 | .000 | .000 | .000 | .000 | .001 | -.036 | .000 | -.010 | -.003 | .007 | -.051 | -.207 | -.041 | -.054 | 1.000 |
| par\_26 | .000 | .000 | .000 | .000 | -.001 | .000 | .399 | .205 | .263 | .261 | .248 | .219 | .007 | .057 | .000 | .001 | .022 | .000 | .000 | .000 | -.076 | .000 | -.021 | .000 | .000 | 1.000 |
| par\_27 | .000 | .000 | .001 | .000 | .001 | .000 | -.069 | .003 | -.007 | .016 | .006 | -.005 | -.050 | -.003 | .000 | -.001 | .012 | .000 | .000 | .000 | -.007 | .000 | -.008 | -.001 | .000 | -.068 | 1.000 |
| par\_28 | -.222 | .001 | .000 | .000 | .001 | .000 | .000 | .000 | .000 | .000 | .000 | .000 | .000 | .000 | .000 | .016 | .000 | -.074 | .000 | -.002 | -.001 | .143 | .007 | .022 | -.002 | .000 | .000 | 1.000 |
| par\_29 | -.218 | .000 | .001 | .000 | .001 | .001 | .000 | .000 | .000 | .000 | .000 | .000 | .000 | .000 | .000 | .007 | .000 | -.054 | .000 | .001 | -.001 | .158 | -.001 | -.006 | .000 | .000 | .000 | .557 | 1.000 |
| par\_30 | -.020 | .000 | .001 | -.001 | .001 | .002 | .000 | .000 | .000 | .000 | .000 | .000 | .000 | .000 | .000 | .006 | .000 | -.110 | .000 | .001 | -.001 | .155 | .002 | -.009 | .000 | .000 | .000 | .447 | .476 | 1.000 |
| par\_31 | .000 | .000 | .000 | .000 | .000 | .000 | .000 | .000 | .000 | .000 | .000 | .000 | .000 | .000 | .658 | .000 | -.069 | .105 | -.013 | .005 | .001 | -.001 | -.003 | .000 | -.001 | .000 | .000 | .000 | -.001 | -.001 | 1.000 |
| par\_32 | -.001 | .755 | .851 | .651 | .090 | .008 | .000 | .000 | -.001 | .000 | .000 | .000 | .000 | .000 | .000 | .000 | -.005 | .002 | .036 | .000 | .393 | -.149 | .002 | .000 | -.022 | -.001 | .000 | .001 | .000 | .000 | .000 | 1.000 |
| par\_33 | .009 | -.001 | -.001 | .000 | -.002 | .000 | -.001 | -.002 | -.001 | -.001 | -.001 | -.001 | .000 | .000 | -.002 | .371 | .006 | .027 | .000 | -.053 | .005 | -.063 | .069 | .528 | -.021 | -.001 | .001 | -.203 | -.242 | -.328 | .002 | -.001 | 1.000 |
| par\_34 | .000 | .000 | .000 | .000 | .000 | .000 | .000 | .000 | .000 | .000 | .000 | .000 | .000 | .000 | -.580 | .000 | .063 | -.085 | .015 | -.014 | .000 | .000 | .000 | .000 | .000 | .000 | .000 | .000 | .000 | .000 | -.649 | .000 | .000 | 1.000 |
| par\_35 | .013 | .000 | .000 | .000 | .000 | .000 | .000 | .000 | .000 | .001 | .001 | .000 | -.001 | .000 | .000 | -.597 | -.003 | -.018 | .000 | .055 | -.005 | -.021 | -.174 | -.675 | .055 | .000 | .000 | -.017 | .001 | .003 | .000 | .000 | -.562 | .000 | 1.000 |
| par\_36 | .000 | -.681 | -.777 | -.591 | -.068 | -.003 | .000 | .000 | .000 | .000 | .000 | .000 | .000 | .000 | .000 | .000 | .003 | -.001 | -.027 | .000 | -.359 | .138 | -.001 | .000 | .031 | .000 | .000 | .000 | .000 | .000 | .000 | -.774 | .001 | .000 | .000 | 1.000 |
| par\_37 | -.001 | .010 | .013 | .001 | .020 | .010 | -.233 | -.317 | -.384 | -.414 | -.387 | -.337 | .040 | -.075 | .000 | -.007 | -.024 | -.003 | .000 | .001 | .063 | .006 | -.030 | -.009 | .000 | -.246 | .015 | .000 | .001 | .000 | .002 | .014 | .011 | -.001 | .003 | -.009 | 1.000 |
| par\_38 | .062 | .002 | .001 | .001 | .002 | -.002 | .002 | .002 | .001 | .001 | .001 | .002 | .002 | .000 | -.001 | .037 | -.002 | .042 | .000 | .012 | -.004 | -.070 | .060 | -.101 | .001 | .001 | -.001 | -.380 | -.348 | -.404 | -.002 | .002 | -.102 | .001 | .074 | -.001 | -.017 | 1.000 |
| par\_39 | -.138 | .000 | .001 | -.001 | .002 | .002 | .000 | .000 | .000 | .000 | .000 | .000 | .000 | .000 | .000 | .014 | .000 | -.043 | .000 | .000 | -.001 | .136 | .004 | .000 | .000 | .000 | .000 | .245 | .272 | .330 | -.001 | .001 | -.124 | .000 | -.005 | -.001 | .000 | -.182 | 1.000 |
| par\_40 | -.113 | .000 | -.001 | .001 | -.001 | -.002 | .000 | .000 | -.001 | .000 | .000 | .000 | .000 | -.001 | .000 | .002 | .000 | .060 | .000 | -.002 | .001 | -.047 | .000 | .024 | -.002 | .000 | .000 | .007 | -.062 | -.367 | .001 | .001 | .137 | .000 | -.013 | .000 | .001 | -.043 | -.213 | 1.000 |
| par\_41 | .517 | .000 | -.001 | .000 | -.001 | -.001 | .000 | .000 | .000 | .000 | .000 | .000 | .000 | .000 | .000 | -.003 | .001 | -.022 | .000 | -.001 | .001 | -.084 | .004 | .010 | -.001 | .000 | .000 | -.117 | -.232 | -.051 | .001 | .000 | .044 | .000 | -.004 | .000 | -.001 | .029 | -.132 | -.027 | 1.000 |
| par\_42 | .522 | -.001 | .000 | .000 | -.001 | .000 | .000 | .000 | .000 | .000 | .000 | .000 | .000 | .000 | .000 | -.018 | .000 | .007 | .000 | .004 | .000 | -.054 | -.009 | -.037 | .003 | .000 | .000 | -.223 | -.097 | .022 | -.001 | -.001 | -.032 | .000 | .025 | .000 | .000 | .067 | -.077 | -.148 | .162 | 1.000 |
| par\_43 | .001 | -.082 | .019 | -.029 | .003 | .389 | .000 | -.001 | -.001 | -.001 | -.001 | .000 | .001 | -.001 | .000 | .000 | .004 | -.002 | -.009 | .000 | .002 | -.016 | .001 | .000 | .001 | .000 | .000 | -.001 | .000 | .000 | .000 | -.005 | .001 | .000 | .000 | .002 | -.002 | -.004 | .000 | -.001 | .000 | .001 | 1.000 |
| par\_44 | -.001 | .077 | .087 | .062 | .426 | .004 | .000 | .000 | .000 | .000 | .000 | -.001 | .000 | .001 | .000 | .000 | -.005 | .002 | .012 | .000 | .021 | -.007 | .002 | .000 | -.008 | -.001 | .001 | .001 | .001 | .000 | .000 | .099 | -.001 | .000 | .000 | -.078 | .015 | .002 | .001 | .000 | -.001 | -.001 | -.004 | 1.000 |
| par\_45 | -.001 | .042 | -.122 | .027 | -.024 | -.115 | .002 | .001 | -.001 | .001 | .000 | .002 | -.001 | -.003 | .000 | -.001 | -.006 | .003 | .013 | .000 | .019 | .008 | .000 | .000 | -.001 | .000 | -.001 | .000 | -.002 | -.004 | .000 | .058 | .001 | .000 | .000 | .002 | -.014 | .003 | -.004 | .006 | .001 | -.003 | -.131 | -.003 | 1.000 |
| par\_46 | .003 | -.051 | .005 | -.021 | -.090 | -.028 | -.001 | .001 | .003 | .000 | .001 | -.001 | .000 | .004 | .000 | .001 | .010 | -.004 | -.019 | .000 | .003 | -.011 | -.002 | .001 | .009 | .002 | .000 | -.002 | .000 | .002 | .000 | -.168 | .002 | .000 | -.001 | .048 | -.020 | -.005 | .000 | -.004 | .001 | .003 | .012 | -.077 | -.345 | 1.000 |
| par\_47 | -.001 | .008 | .036 | -.064 | .424 | .383 | -.001 | -.001 | -.001 | -.001 | -.001 | .000 | .001 | -.002 | .000 | .000 | -.003 | .001 | .006 | .000 | -.012 | .002 | -.002 | -.001 | .000 | -.001 | .001 | .001 | .001 | .002 | .000 | .037 | -.001 | .000 | .001 | -.024 | .018 | .001 | .002 | -.002 | -.001 | .000 | .079 | .097 | -.057 | -.062 | 1.000 |
| par\_48 | .000 | .000 | .000 | .000 | .000 | .000 | -.072 | .009 | .006 | .017 | .009 | -.005 | -.036 | .013 | .000 | .000 | .010 | .000 | .000 | .000 | -.006 | .000 | -.009 | .000 | .000 | -.022 | .436 | .000 | .000 | .000 | .000 | .000 | .001 | .000 | .000 | .000 | .006 | -.001 | .000 | .000 | .000 | .000 | .000 | .000 | -.002 | .001 | .000 | 1.000 |
| par\_49 | .000 | -.001 | .000 | .000 | .000 | .001 | .000 | -.114 | -.025 | -.005 | -.012 | -.008 | -.032 | -.034 | .000 | -.001 | -.006 | .000 | .000 | .000 | .007 | .000 | -.022 | -.001 | .000 | -.007 | -.013 | .000 | .000 | .000 | .000 | .000 | .001 | .000 | .000 | .000 | .022 | -.002 | .000 | .000 | .000 | .000 | .001 | .000 | -.001 | -.001 | .001 | -.013 | 1.000 |
| par\_50 | .000 | .000 | .000 | .000 | .000 | .001 | -.009 | -.036 | -.168 | -.025 | -.013 | .031 | -.044 | -.146 | .000 | -.002 | -.018 | .000 | .000 | .000 | .015 | .000 | .012 | .000 | .000 | -.038 | .006 | .000 | .000 | .000 | .000 | .001 | .000 | .000 | .000 | .000 | .023 | .000 | .000 | .001 | .000 | .000 | .000 | .000 | .003 | -.004 | .001 | -.012 | .017 | 1.000 |
| par\_51 | .000 | .000 | -.001 | .000 | -.001 | -.002 | .096 | .167 | .206 | .209 | .210 | .116 | -.126 | .103 | .000 | .001 | .033 | .000 | .000 | .000 | -.019 | .000 | .019 | -.001 | .000 | .126 | -.016 | .000 | .000 | .001 | -.001 | -.001 | -.003 | .000 | .001 | .001 | -.190 | .004 | .000 | -.001 | .000 | .000 | -.002 | -.001 | .001 | .002 | -.002 | .005 | -.059 | -.103 | 1.000 |
| par\_52 | .000 | -.001 | .000 | .000 | .000 | .001 | .020 | .001 | -.016 | -.181 | -.115 | .007 | .717 | -.088 | .000 | .002 | -.024 | .001 | .000 | .000 | -.002 | .000 | .015 | .002 | .000 | .019 | -.058 | .000 | .000 | .000 | .000 | -.001 | .000 | .000 | -.001 | .000 | .027 | .001 | .000 | .000 | .000 | .000 | .001 | -.001 | -.001 | .001 | .000 | -.043 | -.041 | -.027 | -.088 | 1.000 |
| par\_53 | .000 | .000 | .000 | .000 | .000 | .001 | -.005 | -.020 | -.017 | -.114 | -.179 | -.016 | .716 | -.064 | .000 | .001 | -.013 | .000 | .000 | .000 | .002 | .000 | .013 | .002 | .000 | -.004 | -.027 | .000 | .000 | .000 | .000 | .000 | .000 | .000 | -.001 | .000 | .037 | .001 | .000 | .000 | .000 | .000 | .001 | .000 | .000 | -.001 | .001 | -.019 | -.014 | -.044 | -.114 | .349 | 1.000 |
| par\_54 | .000 | .001 | .001 | .001 | .000 | -.001 | -.008 | .004 | .035 | .016 | .006 | -.129 | -.033 | .090 | .000 | .000 | .006 | .000 | .000 | .000 | .003 | .000 | -.018 | .000 | .000 | .004 | .003 | .000 | .000 | .000 | .000 | .000 | .001 | .000 | .000 | -.001 | .004 | -.001 | .000 | .000 | .000 | .000 | -.001 | .001 | -.003 | .003 | -.001 | .009 | -.008 | -.087 | .016 | -.043 | -.014 | 1.000 |
| par\_55 | .000 | .000 | .000 | .000 | .000 | .000 | .000 | .000 | .000 | .000 | .000 | .000 | .000 | .000 | .105 | .000 | -.007 | .032 | -.005 | -.002 | .000 | .001 | -.001 | .000 | .000 | .000 | .000 | .000 | .000 | -.001 | .206 | .000 | .001 | -.120 | .000 | .000 | .001 | -.003 | .000 | .001 | .000 | -.001 | .000 | .000 | .000 | .000 | .000 | .000 | .000 | .000 | .000 | .000 | .000 | .000 | 1.000 |
| par\_56 | .000 | .000 | .000 | .000 | .000 | .000 | .000 | .001 | .001 | .001 | .001 | .000 | .000 | .001 | .068 | .001 | -.017 | -.019 | -.018 | .042 | -.004 | .004 | .008 | -.001 | .002 | .001 | .000 | .000 | .002 | .002 | -.365 | .000 | -.007 | .098 | -.001 | .000 | -.005 | .001 | .002 | -.002 | -.002 | .002 | .000 | .000 | .000 | .000 | .000 | .000 | -.001 | .000 | .002 | .000 | -.001 | .000 | -.236 | 1.000 |
| par\_57 | .001 | .000 | .000 | .000 | .000 | .000 | .000 | -.001 | -.001 | -.001 | -.001 | .000 | .000 | .000 | -.158 | -.001 | .012 | .006 | .021 | -.039 | .003 | -.004 | -.007 | .001 | -.002 | .000 | .000 | .000 | -.002 | -.001 | .196 | .000 | .006 | -.037 | .001 | .000 | .003 | .000 | -.002 | .001 | .002 | -.001 | .000 | .000 | .000 | .000 | .000 | .000 | .001 | .000 | -.002 | .000 | .001 | .000 | .088 | -.552 | 1.000 |
| par\_58 | -.019 | .000 | .000 | .000 | -.001 | .000 | .000 | .000 | .000 | -.001 | -.001 | .000 | .002 | .000 | .000 | .266 | .007 | .034 | .000 | -.060 | .007 | .029 | .098 | .434 | -.045 | .000 | .000 | .024 | -.001 | -.004 | .000 | .000 | .321 | .000 | -.371 | .000 | -.005 | -.104 | .007 | .018 | .006 | -.036 | .000 | .000 | .000 | .001 | -.001 | .000 | .000 | -.001 | -.001 | .001 | .001 | .000 | .000 | .000 | .000 | 1.000 |
| par\_59 | .004 | .000 | .000 | .000 | .000 | .000 | -.001 | -.001 | -.002 | -.001 | -.001 | -.001 | -.001 | -.001 | .000 | -.252 | .001 | -.001 | .000 | -.008 | .001 | -.008 | -.017 | .140 | .000 | -.001 | .001 | -.003 | -.013 | -.013 | .000 | .000 | .159 | .000 | -.051 | .000 | .007 | -.165 | -.016 | .015 | .011 | -.006 | .001 | .000 | .001 | .000 | .000 | .001 | .001 | .002 | -.003 | -.002 | -.001 | .001 | .000 | -.001 | .001 | .071 | 1.000 |
| par\_60 | .013 | .000 | .000 | .000 | .001 | .000 | .000 | -.001 | .001 | .001 | .001 | .000 | -.002 | .000 | .000 | -.060 | -.006 | -.039 | .000 | .056 | -.006 | -.025 | -.091 | -.450 | .041 | -.001 | .001 | -.018 | .010 | .014 | .000 | .000 | -.206 | .000 | .211 | .000 | .012 | .085 | .007 | -.027 | -.013 | .034 | .000 | .000 | .000 | .000 | .001 | .001 | .001 | -.001 | .000 | -.001 | -.002 | .000 | .000 | .001 | -.001 | -.297 | -.194 | 1.000 |
| par\_61 | .000 | .001 | .000 | .000 | .001 | .000 | -.030 | -.004 | -.018 | .006 | .000 | -.003 | -.038 | -.018 | .000 | -.001 | .007 | .000 | .000 | .000 | -.004 | .000 | -.003 | -.001 | .000 | -.081 | .437 | .000 | .000 | .000 | .000 | .001 | .001 | .000 | .000 | .000 | .016 | -.001 | .000 | .000 | .000 | .000 | .000 | .001 | .001 | -.001 | .001 | .106 | -.005 | .023 | -.029 | -.043 | -.021 | -.005 | .000 | .000 | .000 | .000 | .001 | .001 | 1.000 |

##### Critical Ratios for Differences between Parameters (Default model)

|  | par\_1 | par\_2 | par\_3 | par\_4 | par\_5 | par\_6 | par\_7 | par\_8 | par\_9 | par\_10 | par\_11 | par\_12 | par\_13 | par\_14 | par\_15 | par\_16 | par\_17 | par\_18 | par\_19 | par\_20 | par\_21 | par\_22 | par\_23 | par\_24 | par\_25 | par\_26 | par\_27 | par\_28 | par\_29 | par\_30 | par\_31 | par\_32 | par\_33 | par\_34 | par\_35 | par\_36 | par\_37 | par\_38 | par\_39 | par\_40 | par\_41 | par\_42 | par\_43 | par\_44 | par\_45 | par\_46 | par\_47 | par\_48 | par\_49 | par\_50 | par\_51 | par\_52 | par\_53 | par\_54 | par\_55 | par\_56 | par\_57 | par\_58 | par\_59 | par\_60 | par\_61 |
| --- | --- | --- | --- | --- | --- | --- | --- | --- | --- | --- | --- | --- | --- | --- | --- | --- | --- | --- | --- | --- | --- | --- | --- | --- | --- | --- | --- | --- | --- | --- | --- | --- | --- | --- | --- | --- | --- | --- | --- | --- | --- | --- | --- | --- | --- | --- | --- | --- | --- | --- | --- | --- | --- | --- | --- | --- | --- | --- | --- | --- | --- |
| par\_1 | .000 |
| par\_2 | 15.589 | .000 |
| par\_3 | 18.082 | 3.685 | .000 |
| par\_4 | 15.962 | -3.721 | -7.100 | .000 |
| par\_5 | -1.159 | -17.414 | -20.206 | -18.238 | .000 |
| par\_6 | -2.075 | -17.388 | -20.415 | -18.399 | -1.262 | .000 |
| par\_7 | 10.045 | -8.945 | -11.316 | -7.810 | 12.741 | 13.661 | .000 |
| par\_8 | 15.431 | -4.993 | -7.287 | -3.064 | 18.638 | 19.527 | 6.175 | .000 |
| par\_9 | 17.119 | -5.444 | -7.858 | -3.542 | 21.665 | 22.787 | 6.716 | -.383 | .000 |
| par\_10 | 18.705 | -4.549 | -6.958 | -2.443 | 23.608 | 24.754 | 8.399 | 1.271 | 2.033 | .000 |
| par\_11 | 17.685 | -4.759 | -7.141 | -2.720 | 22.048 | 23.118 | 7.661 | .757 | 1.352 | -.801 | .000 |
| par\_12 | 12.328 | -7.471 | -9.826 | -6.027 | 15.365 | 16.305 | 2.477 | -4.071 | -4.286 | -6.197 | -5.449 | .000 |
| par\_13 | -2.158 | -17.989 | -20.734 | -19.524 | -1.278 | .183 | -14.661 | -20.679 | -24.777 | -25.705 | -23.795 | -17.421 | .000 |
| par\_14 | -6.051 | -19.893 | -22.660 | -21.985 | -7.026 | -5.702 | -18.293 | -24.478 | -30.194 | -32.288 | -29.786 | -20.377 | -7.872 | .000 |
| par\_15 | 19.028 | -2.272 | -4.518 | .155 | 22.524 | 23.405 | 9.268 | 3.709 | 4.378 | 3.085 | 3.386 | 7.190 | 24.748 | 27.869 | .000 |
| par\_16 | 11.047 | -4.149 | -6.079 | -2.361 | 12.493 | 13.035 | 4.175 | .095 | .324 | -.634 | -.349 | 2.624 | 13.340 | 15.374 | -2.707 | .000 |
| par\_17 | -5.720 | -18.480 | -20.877 | -19.228 | -5.548 | -4.847 | -14.509 | -19.491 | -21.316 | -22.805 | -21.779 | -16.698 | -5.234 | -2.162 | -21.533 | -14.410 | .000 |
| par\_18 | .274 | -13.799 | -16.000 | -13.439 | 1.126 | 1.767 | -7.675 | -12.169 | -12.870 | -14.064 | -13.464 | -9.499 | 1.766 | 4.361 | -15.990 | -9.729 | 4.902 | .000 |
| par\_19 | -3.810 | -17.517 | -19.914 | -17.959 | -3.397 | -2.651 | -12.538 | -17.325 | -18.796 | -20.180 | -19.309 | -14.566 | -2.925 | .182 | -20.337 | -13.081 | 1.682 | -3.346 | .000 |
| par\_20 | -2.106 | -15.250 | -17.432 | -15.102 | -1.516 | -.910 | -9.737 | -14.058 | -14.855 | -16.018 | -15.408 | -11.506 | -1.026 | 1.457 | -17.264 | -10.964 | 2.485 | -1.829 | 1.061 | .000 |
| par\_21 | 5.836 | -12.573 | -15.824 | -11.356 | 7.398 | 8.058 | -2.631 | -7.189 | -7.484 | -8.646 | -8.159 | -4.395 | 8.427 | 11.205 | -10.836 | -5.938 | 10.222 | 4.609 | 8.626 | 6.575 | .000 |
| par\_22 | -6.061 | -17.160 | -19.020 | -17.506 | -6.113 | -5.525 | -13.746 | -17.937 | -19.017 | -20.185 | -19.505 | -15.500 | -5.844 | -3.383 | -20.835 | -14.468 | -1.360 | -5.625 | -2.819 | -3.495 | -10.018 | .000 |
| par\_23 | -.223 | -14.945 | -17.301 | -14.971 | .709 | 1.461 | -9.050 | -13.904 | -14.849 | -16.164 | -15.421 | -11.015 | 1.465 | 4.553 | -17.367 | -11.458 | 4.819 | -.436 | 3.220 | 1.760 | -5.493 | 5.667 | .000 |
| par\_24 | 10.260 | -3.474 | -5.262 | -1.731 | 11.416 | 11.884 | 4.191 | .544 | .766 | -.083 | .164 | 2.808 | 12.083 | 13.840 | -1.978 | .543 | 13.397 | 9.344 | 12.189 | 10.275 | 5.818 | 13.642 | 10.833 | .000 |
| par\_25 | -3.930 | -18.236 | -20.820 | -19.463 | -3.599 | -2.587 | -14.652 | -20.193 | -23.098 | -24.909 | -23.459 | -17.127 | -3.124 | 1.697 | -23.885 | -13.722 | 2.869 | -3.153 | .802 | -.543 | -9.098 | 3.638 | -3.031 | -12.491 | .000 |
| par\_26 | 11.005 | -7.866 | -10.185 | -6.506 | 13.589 | 14.456 | 1.735 | -4.555 | -4.844 | -6.413 | -5.770 | -.865 | 15.372 | 18.873 | -7.678 | -3.125 | 15.285 | 8.593 | 13.350 | 10.578 | 3.691 | 14.472 | 9.950 | -3.259 | 15.395 | .000 |
| par\_27 | -1.155 | -17.201 | -19.879 | -18.348 | .044 | 1.310 | -12.615 | -18.952 | -22.087 | -24.315 | -22.567 | -15.600 | 1.388 | 7.560 | -22.847 | -12.565 | 5.706 | -1.116 | 3.463 | 1.554 | -7.434 | 6.206 | -.692 | -11.465 | 3.770 | -13.452 | .000 |
| par\_28 | 11.421 | -5.879 | -8.092 | -4.166 | 14.910 | 15.672 | 3.629 | -1.460 | -1.302 | -2.539 | -2.132 | 1.675 | 16.406 | 19.248 | -4.889 | -1.225 | 16.320 | 9.810 | 14.681 | 12.001 | 5.737 | 16.937 | 11.659 | -1.558 | 16.520 | 2.311 | 15.079 | .000 |
| par\_29 | 11.146 | -5.926 | -8.126 | -4.230 | 14.474 | 15.219 | 3.451 | -1.563 | -1.415 | -2.632 | -2.230 | 1.525 | 15.895 | 18.673 | -4.947 | -1.305 | 15.979 | 9.687 | 14.367 | 11.787 | 5.556 | 16.797 | 11.343 | -1.613 | 16.096 | 2.158 | 14.630 | -.172 | .000 |
| par\_30 | 15.624 | -4.136 | -6.376 | -2.082 | 18.706 | 19.536 | 6.381 | 1.034 | 1.429 | .156 | .518 | 4.354 | 20.562 | 23.570 | -2.548 | .696 | 19.322 | 12.044 | 17.625 | 14.514 | 8.243 | 19.837 | 14.482 | .174 | 20.210 | 4.925 | 18.945 | 3.188 | 3.397 | .000 |
| par\_31 | 20.077 | -1.648 | -3.895 | .903 | 23.744 | 24.637 | 10.217 | 4.599 | 5.362 | 4.066 | 4.345 | 8.127 | 26.079 | 29.222 | 1.466 | 3.375 | 22.505 | 16.967 | 21.382 | 17.875 | 11.695 | 21.630 | 18.257 | 2.577 | 25.040 | 8.585 | 24.092 | 5.723 | 5.768 | 3.398 | .000 |
| par\_32 | 17.941 | 4.197 | .693 | 7.380 | 20.023 | 20.093 | 11.366 | 7.436 | 7.989 | 7.115 | 7.293 | 9.914 | 20.459 | 22.319 | 4.733 | 6.249 | 20.677 | 15.974 | 19.807 | 17.373 | 15.771 | 18.964 | 17.215 | 5.440 | 20.574 | 10.263 | 19.647 | 8.226 | 8.257 | 6.545 | 4.124 | .000 |
| par\_33 | 8.888 | -5.409 | -7.293 | -3.821 | 10.002 | 10.513 | 2.279 | -1.595 | -1.461 | -2.380 | -2.087 | .797 | 10.717 | 12.643 | -4.266 | -1.791 | 12.176 | 7.820 | 10.893 | 9.064 | 4.106 | 11.918 | 8.807 | -2.475 | 11.321 | 1.311 | 10.059 | -.414 | -.322 | -2.033 | -4.918 | -7.442 | .000 |
| par\_34 | 6.501 | -10.715 | -13.051 | -9.917 | 8.421 | 9.243 | -2.875 | -8.094 | -8.628 | -9.998 | -9.367 | -4.961 | 9.766 | 12.976 | -9.230 | -6.139 | 11.411 | 4.783 | 9.372 | 7.006 | .078 | 10.951 | 6.029 | -5.960 | 10.465 | -4.057 | 8.536 | -6.066 | -5.864 | -8.784 | -9.751 | -13.062 | -4.211 | .000 |
| par\_35 | 5.937 | -7.789 | -9.697 | -6.484 | 6.896 | 7.401 | -.654 | -4.504 | -4.518 | -5.451 | -5.118 | -2.145 | 7.528 | 9.474 | -7.161 | -3.057 | 9.359 | 5.029 | 8.112 | 6.883 | 1.356 | 9.612 | 5.338 | -3.070 | 8.516 | -1.574 | 6.925 | -3.211 | -3.122 | -5.171 | -7.819 | -9.807 | -1.894 | 1.357 | .000 |
| par\_36 | 5.373 | -8.251 | -9.719 | -7.670 | 6.666 | 7.522 | -3.058 | -7.833 | -8.221 | -9.447 | -8.911 | -4.955 | 7.819 | 10.554 | -11.071 | -6.164 | 9.692 | 4.239 | 7.981 | 6.206 | -.286 | 10.602 | 5.090 | -6.018 | 8.846 | -4.142 | 6.890 | -6.015 | -5.833 | -8.502 | -11.912 | -9.777 | -4.339 | -.433 | -1.619 | .000 |
| par\_37 | 6.015 | -12.108 | -14.604 | -11.656 | 8.471 | 9.433 | -4.055 | -8.904 | -9.514 | -10.734 | -10.126 | -5.908 | 10.348 | 13.882 | -13.957 | -7.329 | 10.898 | 4.330 | 9.090 | 6.585 | -1.052 | 10.809 | 5.388 | -6.969 | 10.700 | -5.136 | 8.618 | -7.734 | -7.483 | -10.794 | -15.004 | -14.563 | -5.278 | -1.214 | -2.267 | -.617 | .000 |
| par\_38 | 5.067 | -10.859 | -13.076 | -10.020 | 6.326 | 7.014 | -3.487 | -8.249 | -8.667 | -9.892 | -9.346 | -5.381 | 7.285 | 10.014 | -11.462 | -6.601 | 9.252 | 3.948 | 7.669 | 5.871 | -.719 | 9.163 | 4.824 | -6.038 | 8.247 | -4.556 | 6.375 | -5.449 | -5.354 | -7.513 | -12.298 | -13.107 | -4.439 | -.847 | -2.001 | -.383 | .167 | .000 |
| par\_39 | 5.577 | -11.574 | -13.980 | -10.987 | 8.018 | 8.913 | -3.938 | -9.367 | -10.137 | -11.600 | -10.869 | -6.133 | 9.518 | 13.058 | -12.996 | -6.978 | 10.745 | 4.329 | 8.909 | 6.581 | -.710 | 11.370 | 5.484 | -6.652 | 10.196 | -5.130 | 8.142 | -8.163 | -8.034 | -12.179 | -13.961 | -13.957 | -4.687 | -.863 | -1.998 | -.331 | .324 | .092 | .000 |
| par\_40 | 3.205 | -13.736 | -16.225 | -13.722 | 5.285 | 6.273 | -7.099 | -12.720 | -14.111 | -15.723 | -14.767 | -9.441 | 6.822 | 10.905 | -16.460 | -9.105 | 8.773 | 2.359 | 6.821 | 4.620 | -3.218 | 8.692 | 3.161 | -8.605 | 7.839 | -8.208 | 5.389 | -10.050 | -9.459 | -11.378 | -17.522 | -16.143 | -7.299 | -3.637 | -3.903 | -2.781 | -2.753 | -2.280 | -2.630 | .000 |
| par\_41 | 12.808 | -8.656 | -10.934 | -7.449 | 11.200 | 11.995 | -.064 | -5.217 | -5.421 | -6.726 | -6.203 | -2.086 | 12.621 | 15.645 | -8.681 | -4.042 | 13.272 | 7.096 | 11.574 | 9.141 | 2.495 | 12.438 | 8.421 | -4.110 | 13.044 | -1.308 | 11.347 | -3.270 | -2.969 | -5.863 | -9.565 | -11.000 | -2.284 | 2.603 | .582 | 2.814 | 4.025 | 3.262 | 3.346 | 6.300 | .000 |
| par\_42 | 12.324 | -9.130 | -11.437 | -8.017 | 10.809 | 11.629 | -.646 | -5.866 | -6.150 | -7.486 | -6.931 | -2.703 | 12.267 | 15.389 | -9.372 | -4.472 | 12.940 | 6.800 | 11.220 | 8.800 | 2.015 | 12.306 | 7.962 | -4.433 | 12.723 | -1.891 | 10.959 | -3.644 | -3.683 | -6.717 | -10.264 | -11.483 | -2.625 | 2.091 | .183 | 2.344 | 3.498 | 2.845 | 2.930 | 5.520 | -.589 | .000 |
| par\_43 | 6.789 | -11.629 | -14.572 | -11.431 | 9.581 | 13.323 | -4.214 | -10.101 | -11.199 | -12.845 | -11.948 | -6.627 | 11.779 | 16.119 | -13.996 | -7.172 | 11.858 | 4.817 | 9.787 | 7.104 | -.658 | 11.327 | 6.082 | -6.818 | 11.886 | -5.484 | 9.796 | -7.562 | -7.302 | -10.725 | -15.061 | -14.396 | -5.050 | -.823 | -2.007 | -.251 | .498 | .212 | .126 | 3.389 | -3.742 | -3.193 | .000 |
| par\_44 | 7.843 | -11.555 | -14.174 | -10.940 | 13.836 | 11.857 | -3.080 | -8.966 | -9.864 | -11.481 | -10.648 | -5.467 | 13.025 | 17.259 | -12.853 | -6.413 | 12.678 | 5.665 | 10.784 | 7.899 | .277 | 12.124 | 7.022 | -6.151 | 12.914 | -4.393 | 11.018 | -6.560 | -6.318 | -9.645 | -13.901 | -14.199 | -4.333 | .208 | -1.303 | .643 | 1.674 | 1.124 | 1.215 | 4.527 | -2.724 | -2.155 | 1.225 | .000 |
| par\_45 | -.063 | -16.896 | -18.903 | -17.942 | 1.574 | 2.795 | -12.208 | -18.304 | -21.541 | -23.596 | -21.918 | -14.934 | 3.508 | 10.145 | -22.292 | -12.044 | 6.682 | -.355 | 4.458 | 2.314 | -6.765 | 7.027 | .211 | -10.997 | 5.250 | -13.084 | 1.627 | -14.445 | -13.990 | -18.318 | -23.554 | -19.457 | -9.543 | -7.741 | -6.385 | -6.171 | -7.593 | -5.653 | -7.270 | -4.374 | -10.633 | -10.209 | -8.387 | -10.104 | .000 |
| par\_46 | .622 | -16.120 | -19.018 | -17.137 | 2.375 | 3.762 | -11.433 | -17.541 | -20.562 | -22.545 | -20.963 | -14.127 | 4.526 | 10.897 | -21.528 | -11.617 | 7.220 | .124 | 4.922 | 2.757 | -6.168 | 7.395 | .770 | -10.633 | 5.985 | -12.379 | 2.554 | -13.804 | -13.385 | -17.652 | -22.772 | -18.084 | -9.149 | -7.087 | -5.996 | -5.713 | -6.771 | -5.100 | -6.566 | -3.580 | -9.991 | -9.573 | -7.999 | -8.929 | .885 | .000 |
| par\_47 | 6.506 | -12.426 | -15.121 | -11.835 | 12.278 | 13.302 | -4.820 | -10.829 | -12.124 | -13.838 | -12.856 | -7.304 | 11.889 | 16.557 | -14.787 | -7.577 | 11.685 | 4.533 | 9.666 | 6.863 | -1.073 | 11.250 | 5.790 | -7.158 | 11.844 | -6.086 | 9.708 | -8.147 | -7.867 | -11.417 | -15.885 | -15.052 | -5.404 | -1.298 | -2.333 | -.650 | -.008 | -.183 | -.355 | 2.981 | -4.264 | -3.715 | -.577 | -1.914 | 8.497 | 7.543 | .000 |
| par\_48 | 5.960 | -13.081 | -15.673 | -13.051 | 9.124 | 10.390 | -5.680 | -12.082 | -13.734 | -15.635 | -14.444 | -8.429 | 11.795 | 17.334 | -16.078 | -8.238 | 11.445 | 4.009 | 9.255 | 6.418 | -1.798 | 10.914 | 5.238 | -7.718 | 11.658 | -7.040 | 12.428 | -9.101 | -8.789 | -12.532 | -17.227 | -15.574 | -5.994 | -2.121 | -2.874 | -1.354 | -.920 | -.865 | -1.203 | 2.226 | -5.153 | -4.609 | -1.552 | -2.872 | 8.343 | 7.294 | -1.013 | .000 |
| par\_49 | 5.896 | -12.971 | -15.547 | -12.892 | 8.897 | 10.119 | -5.718 | -11.260 | -13.208 | -15.110 | -13.985 | -8.229 | 11.371 | 16.342 | -15.815 | -8.149 | 11.251 | 4.006 | 9.169 | 6.393 | -1.745 | 10.844 | 5.180 | -7.649 | 11.411 | -6.936 | 9.111 | -8.951 | -8.648 | -12.327 | -16.950 | -15.458 | -5.929 | -2.044 | -2.826 | -1.299 | -.854 | -.815 | -1.133 | 2.235 | -5.041 | -4.500 | -1.461 | -2.753 | 8.112 | 7.101 | -.928 | .064 | .000 |
| par\_50 | 1.050 | -16.333 | -19.044 | -17.356 | 3.302 | 4.808 | -11.522 | -17.637 | -20.020 | -23.117 | -21.466 | -14.505 | 5.987 | 12.847 | -21.912 | -11.573 | 7.723 | .396 | 5.449 | 3.076 | -6.069 | 7.842 | 1.121 | -10.568 | 6.907 | -12.340 | 3.459 | -13.907 | -13.459 | -17.874 | -23.203 | -18.831 | -9.068 | -7.011 | -5.878 | -5.488 | -6.848 | -4.961 | -6.484 | -3.375 | -9.996 | -9.563 | -8.011 | -9.341 | 1.801 | .611 | -7.837 | -7.361 | -7.226 | .000 |
| par\_51 | -.556 | -17.198 | -19.930 | -18.503 | 1.059 | 2.569 | -13.561 | -20.349 | -25.003 | -27.379 | -25.227 | -16.575 | 3.076 | 12.065 | -23.444 | -12.492 | 6.604 | -.693 | 4.204 | 2.053 | -7.235 | 6.858 | -.176 | -11.346 | 5.088 | -14.503 | 1.068 | -15.220 | -14.737 | -19.310 | -24.754 | -19.680 | -9.912 | -8.434 | -6.723 | -6.684 | -8.070 | -6.155 | -8.052 | -5.133 | -11.363 | -10.972 | -9.968 | -11.269 | -.817 | -1.947 | -9.943 | -9.803 | -9.220 | -2.901 | .000 |
| par\_52 | .326 | -16.726 | -19.449 | -17.877 | 2.304 | 3.816 | -12.367 | -18.549 | -22.012 | -22.870 | -21.632 | -15.140 | 9.093 | 12.226 | -22.610 | -11.996 | 7.166 | -.097 | 4.887 | 2.613 | -6.596 | 7.398 | .535 | -10.926 | 6.096 | -13.242 | 2.334 | -14.506 | -14.041 | -18.528 | -23.913 | -19.216 | -9.454 | -7.657 | -6.260 | -6.031 | -7.665 | -5.502 | -7.195 | -4.169 | -10.617 | -10.202 | -8.899 | -10.213 | .641 | -.522 | -8.789 | -8.321 | -8.054 | -1.322 | 1.646 | .000 |
| par\_53 | 1.673 | -15.928 | -18.624 | -16.804 | 4.074 | 5.534 | -10.776 | -16.938 | -20.008 | -21.268 | -19.295 | -13.485 | 12.310 | 13.552 | -21.128 | -11.168 | 8.157 | .834 | 5.903 | 3.474 | -5.515 | 8.198 | 1.634 | -10.224 | 7.488 | -11.760 | 4.195 | -13.286 | -12.858 | -17.171 | -22.399 | -18.423 | -8.692 | -6.381 | -5.513 | -4.972 | -6.091 | -4.452 | -5.795 | -2.635 | -9.371 | -8.921 | -7.124 | -8.451 | 2.717 | 1.555 | -6.882 | -6.278 | -6.121 | 1.084 | 3.807 | 2.952 | .000 |
| par\_54 | 4.067 | -14.400 | -17.042 | -14.777 | 6.987 | 8.334 | -8.045 | -14.322 | -16.815 | -18.602 | -17.174 | -10.208 | 9.756 | 16.497 | -18.364 | -9.587 | 9.987 | 2.566 | 7.752 | 5.079 | -3.488 | 9.679 | 3.601 | -8.877 | 9.859 | -9.225 | 7.263 | -11.002 | -10.641 | -14.636 | -19.567 | -16.894 | -7.240 | -4.037 | -4.090 | -2.996 | -3.169 | -2.492 | -3.253 | .087 | -7.060 | -6.555 | -3.990 | -5.325 | 6.004 | 4.904 | -3.559 | -2.720 | -2.678 | 4.655 | 7.502 | 5.891 | 3.707 | .000 |
| par\_55 | 2.466 | -15.453 | -18.132 | -16.174 | 5.087 | 6.520 | -9.930 | -16.190 | -19.016 | -21.040 | -19.487 | -12.672 | 8.003 | 14.730 | -21.046 | -10.670 | 8.762 | 1.408 | 6.505 | 3.994 | -4.865 | 8.688 | 2.279 | -9.800 | 8.313 | -10.966 | 5.304 | -12.570 | -12.161 | -16.377 | -23.232 | -17.948 | -8.236 | -5.406 | -5.063 | -4.341 | -5.087 | -3.822 | -4.977 | -1.744 | -8.639 | -8.168 | -6.107 | -7.443 | 3.874 | 2.715 | -5.797 | -5.138 | -5.021 | 2.427 | 5.260 | 3.685 | 1.301 | -2.476 | .000 |
| par\_56 | -2.548 | -18.106 | -20.835 | -19.622 | -1.846 | -.472 | -14.753 | -20.791 | -24.799 | -26.933 | -24.996 | -17.529 | -.851 | 6.570 | -25.255 | -13.484 | 4.797 | -2.036 | 2.508 | .737 | -8.634 | 5.506 | -1.797 | -12.222 | 2.472 | -15.464 | -2.008 | -16.522 | -16.029 | -20.629 | -23.597 | -20.562 | -10.863 | -10.292 | -7.707 | -8.038 | -10.394 | -7.512 | -9.740 | -7.102 | -12.777 | -12.443 | -11.928 | -13.149 | -4.001 | -4.949 | -12.021 | -12.059 | -11.636 | -6.433 | -3.819 | -5.289 | -7.152 | -10.039 | -7.402 | .000 |
| par\_57 | .197 | -16.668 | -19.369 | -17.747 | 2.003 | 3.411 | -12.112 | -18.268 | -21.608 | -23.679 | -21.959 | -14.866 | 4.161 | 11.164 | -21.235 | -11.956 | 7.004 | -.179 | 4.727 | 2.474 | -6.593 | 7.219 | .424 | -10.913 | 5.681 | -13.003 | 2.064 | -14.368 | -13.912 | -18.298 | -25.240 | -19.145 | -9.457 | -7.512 | -6.277 | -6.029 | -7.499 | -5.505 | -7.129 | -4.166 | -10.530 | -10.106 | -8.722 | -10.011 | .407 | -.669 | -8.584 | -8.219 | -7.978 | -1.429 | 1.327 | -.212 | -2.400 | -5.822 | -3.776 | 3.712 | .000 |
| par\_58 | 7.598 | -8.853 | -11.051 | -7.674 | 9.333 | 10.035 | -.757 | -5.553 | -5.737 | -6.939 | -6.461 | -2.637 | 10.438 | 13.127 | -8.805 | -5.118 | 11.783 | 6.291 | 10.165 | 7.854 | 1.727 | 11.867 | 7.575 | -5.710 | 10.926 | -1.899 | 9.426 | -3.940 | -3.736 | -6.287 | -9.629 | -11.119 | -3.192 | 1.760 | .043 | 2.031 | 2.980 | 2.293 | 2.612 | 5.184 | -.658 | -.157 | 2.686 | 1.762 | 8.743 | 8.191 | 3.135 | 3.898 | 3.814 | 8.120 | 9.314 | 8.667 | 7.591 | 5.578 | 6.951 | 10.622 | 8.627 | .000 |
| par\_59 | 7.539 | -9.485 | -11.748 | -8.428 | 9.362 | 10.123 | -1.364 | -6.374 | -6.671 | -7.955 | -7.418 | -3.342 | 10.598 | 13.523 | -9.760 | -4.467 | 11.795 | 5.943 | 10.127 | 7.891 | 1.288 | 11.620 | 6.956 | -5.256 | 11.251 | -2.541 | 9.476 | -4.571 | -4.377 | -7.071 | -10.631 | -11.797 | -3.395 | 1.302 | -.345 | 1.615 | 2.576 | 1.863 | 2.159 | 4.886 | -1.223 | -.703 | 2.241 | 1.262 | 8.748 | 8.142 | 2.714 | 3.523 | 3.437 | 8.090 | 9.379 | 8.673 | 7.501 | 5.326 | 6.811 | 10.789 | 8.627 | -.517 | .000 |
| par\_60 | 7.157 | -9.448 | -11.675 | -8.372 | 8.793 | 9.514 | -1.473 | -6.339 | -6.609 | -7.846 | -7.335 | -3.391 | 9.908 | 12.693 | -9.637 | -4.856 | 11.272 | 5.568 | 9.695 | 7.841 | 1.116 | 11.141 | 6.393 | -4.181 | 10.825 | -2.614 | 8.885 | -4.557 | -4.453 | -7.118 | -10.476 | -11.728 | -2.928 | 1.120 | -.510 | 1.440 | 2.334 | 1.909 | 1.963 | 4.467 | -1.316 | -.843 | 1.996 | 1.056 | 8.174 | 7.605 | 2.446 | 3.209 | 3.132 | 7.525 | 8.758 | 8.083 | 6.978 | 4.922 | 6.326 | 10.112 | 8.048 | -.544 | -.127 | .000 |
| par\_61 | 7.109 | -12.050 | -14.587 | -11.705 | 10.194 | 11.351 | -4.200 | -10.277 | -11.406 | -13.255 | -12.260 | -6.731 | 12.555 | 17.298 | -14.267 | -7.210 | 12.257 | 4.978 | 10.154 | 7.295 | -.603 | 11.671 | 6.300 | -6.837 | 12.523 | -5.357 | 13.613 | -7.660 | -7.388 | -10.903 | -15.363 | -14.532 | -5.058 | -.770 | -1.981 | -.189 | .608 | .284 | .211 | 3.604 | -3.760 | -3.198 | .092 | -1.185 | 9.504 | 8.525 | .673 | 1.828 | 1.623 | 8.743 | 10.677 | 9.463 | 7.617 | 4.307 | 6.592 | 12.835 | 9.390 | -2.674 | -2.223 | -1.972 | .000 |

##### Model Fit Summary

##### CMIN

| Model | NPAR | CMIN | DF | P | CMIN/DF |
| --- | --- | --- | --- | --- | --- |
| Default model | 61 | 637.638 | 215 | .000 | 2.966 |
| Saturated model | 276 | .000 | 0 |
| Independence model | 23 | 10682.199 | 253 | .000 | 42.222 |

##### RMR, GFI

| Model | RMR | GFI | AGFI | PGFI |
| --- | --- | --- | --- | --- |
| Default model | .057 | .924 | .902 | .720 |
| Saturated model | .000 | 1.000 |  |  |
| Independence model | .307 | .293 | .229 | .269 |

##### Baseline Comparisons

| Model | NFI Delta1 | RFI rho1 | IFI Delta2 | TLI rho2 | CFI |
| --- | --- | --- | --- | --- | --- |
| Default model | .940 | .930 | .960 | .952 | .959 |
| Saturated model | 1.000 |  | 1.000 |  | 1.000 |
| Independence model | .000 | .000 | .000 | .000 | .000 |

##### Parsimony-Adjusted Measures

| Model | PRATIO | PNFI | PCFI |
| --- | --- | --- | --- |
| Default model | .850 | .799 | .815 |
| Saturated model | .000 | .000 | .000 |
| Independence model | 1.000 | .000 | .000 |

##### NCP

| Model | NCP | LO 90 | HI 90 |
| --- | --- | --- | --- |
| Default model | 422.638 | 350.671 | 502.233 |
| Saturated model | .000 | .000 | .000 |
| Independence model | 10429.199 | 10094.384 | 10770.342 |

##### FMIN

| Model | FMIN | F0 | LO 90 | HI 90 |
| --- | --- | --- | --- | --- |
| Default model | .946 | .627 | .520 | .745 |
| Saturated model | .000 | .000 | .000 | .000 |
| Independence model | 15.849 | 15.474 | 14.977 | 15.980 |

##### RMSEA

| Model | RMSEA | LO 90 | HI 90 | PCLOSE |
| --- | --- | --- | --- | --- |
| Default model | .054 | .049 | .059 | .085 |
| Independence model | .247 | .243 | .251 | .000 |

##### AIC

| Model | AIC | BCC | BIC | CAIC |
| --- | --- | --- | --- | --- |
| Default model | 759.638 | 764.142 | 1035.035 | 1096.035 |
| Saturated model | 552.000 | 572.382 | 1798.061 | 2074.061 |
| Independence model | 10728.199 | 10729.898 | 10832.038 | 10855.038 |

##### ECVI

| Model | ECVI | LO 90 | HI 90 | MECVI |
| --- | --- | --- | --- | --- |
| Default model | 1.127 | 1.020 | 1.245 | 1.134 |
| Saturated model | .819 | .819 | .819 | .849 |
| Independence model | 15.917 | 15.420 | 16.423 | 15.920 |

##### HOELTER

| Model | HOELTER .05 | HOELTER .01 |
| --- | --- | --- |
| Default model | 265 | 282 |
| Independence model | 19 | 20 |

##### Execution time summary

|  |  |
| --- | --- |
| Minimization: | .033 |
| Miscellaneous: | .493 |
| Bootstrap: | .000 |
| Total: | .526 |
