# Supplementary figures and images for: Analysis of factors influencing changes in medical behavior under the context of DRG payment method reform: a structural equation modeling approach
Source: Front Public Health. 2025 Sep 12;13:1524215. doi: 10.3389/fpubh.2025.1524215 (PMC12463830; doi:10.3389/fpubh.2025.1524215)

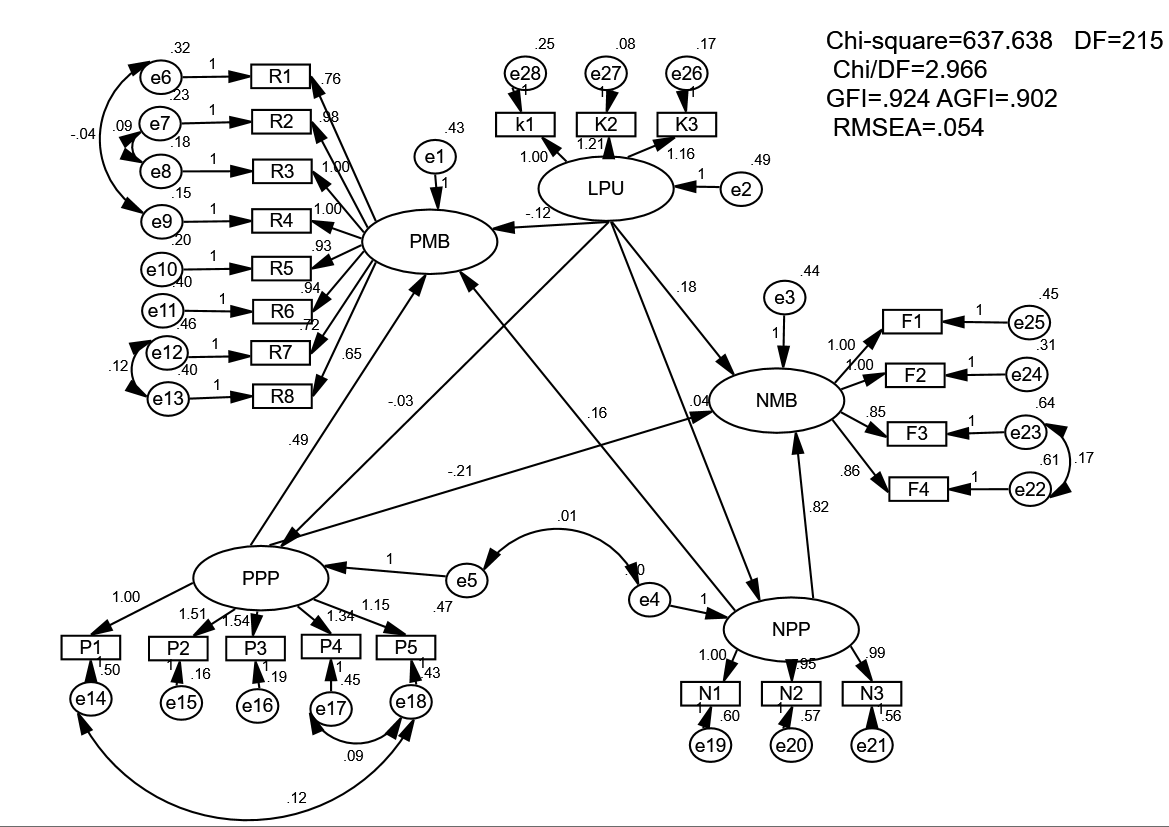

Supplement: Supplementary file 1 [file Data_Sheet_1.ZIP › SEM模型数据/S2Model data/SEMpicture1.png]
